# Supplementary material for: Copper(I) Complexes with Terphenyl-Substituted NPN Ligands Bearing Pyridyl Groups: Synthesis, Characterization, and Catalytic Studies in the S-Arylation of Thiols
Source: Molecules. 2025 Jul 29;30(15):3167. doi: 10.3390/molecules30153167 (PMC12348607; doi:10.3390/molecules30153167)
Supplement: Supplementary file 1 [file molecules-30-03167-s001.zip › molecules-3759414-supplementary/CheckCIF PLATON_report_global_CIF_with_five_structures - 15072025.pdf]

## checkCIF (basic structural check) running

*Checking for embedded fcf data in CIF ...*
*Found embedded fcf data in CIF. Extracting fcf data from uploaded CIF, please wait* . . . . . . . . . . . . . . . . . . . .

## checkCIF/PLATON (basic structural check)

Structure factors have been supplied for datablock(s) 1, 2b, 2c, 3b, 4

THIS REPORT IS FOR GUIDANCE ONLY. IF USED AS PART OF A REVIEW PROCEDURE FOR PUBLICATION, IT SHOULD NOT REPLACE THE EXPERTISE OF AN EXPERIENCED CRYSTALLOGRAPHIC REFEREE.

No syntax errors found. CIF dictionary
Please wait while processing .... Interpreting this report

Structure factor report

# Datablock: 1

| | | | |
|---|---|---|---|
| Bond precision: | C-C = 0.0045 A | | Wavelength=0.71073 |
| Cell: | a=13.1751(3) | b=86.924(2) | c=8.3448(3) |
| | alpha=90 | beta=90 | gamma=90 |
| Temperature: | 173 K | | |

| | Calculated | Reported |
|---|---|---|
| Volume | 9556.7(5) | 9556.7(5) |
| Space group | F d d 2 | F d d 2 |
| Hall group | F 2 -2d | F 2 -2d |
| Moiety formula | C22 H23 O2 P, C5 H5 N O | C22 H23 O2 P, C5 H5 N O |
| Sum formula | C27 H28 N O3 P | C27 H28 N O3 P |
| Mr | 445.47 | 445.47 |
| Dx,g cm-3 | 1.238 | 1.238 |
| Z | 16 | 16 |
| Mu (mm-1) | 0.143 | 0.143 |
| F000 | 3776.0 | 3776.0 |
| F000' | 3779.18 | |
| h,k,lmax | 18,124,11 | 18,124,11 |
| Nref | 7422[ 3944] | 7316 |
| Tmin,Tmax | 0.953,0.965 | 0.693,0.746 |
| Tmin' | 0.953 | |

Correction method= # Reported T Limits: Tmin=0.693 Tmax=0.746
AbsCorr = MULTI-SCAN

Data completeness= 1.85/0.99 Theta(max)= 30.687

R(reflections)= 0.0473( 6888) wR2(reflections)= 0.1271( 7316)

S = 1.134 Npar= 305

The following ALERTS were generated. Each ALERT has the format
**test-name_ALERT_alert-type_alert-level**.
Click on the hyperlinks for more details of the test.

### Alert level B

PLAT772_ALERT_2_B Suspect O-H Bond in CIF: O3 --H2 .. 1.49 Ang.

**Author Response: The suspected O-H...O hydrogen bond involving atoms O3 and H2 corresponds to a well-defined intermolecular hydrogen bond between the phosphinic acid moiety (P(=O)(OH)) and the 2-pyridone molecule, as shown in Figure 2 of the manuscript. The hydrogen atom H2 was located in the difference Fourier map and refined isotropically. This interaction is also supported by the short O...O distance of 2.446(3) and the O_H...O angle consistent with a hydrogen bond. The presence of this hydrogen bond confirms the formation of a hydrogen-bonded adduct between the terphenylphosphinic acid and 2-pyridone, which is discussed and illustrated in Section 2.1 of the manuscript. Therefore, this alert is not indicative of an error but rather reflects a chemically meaningful hydrogen bond that has been addressed and discussed in the article.**

## Alert level C

PLAT340_ALERT_3_C Low Bond Precision on C-C Bonds .............. 0.00454 Ang.

**Author Response: The bond precision value reported (0.00454 Ang) is slightly higher than the ideal target (\\sim 0.002-0.003 Ang) for C-C bonds in light-atom structures. However, this value remains within acceptable limits for a structure of this complexity, particularly considering the presence of heavy atoms (P) and flexible aromatic terphenyl groups. The refinement was carried out using high-quality data collected at 173 K with a completeness of 99% and multi-scan absorption correction. The displacement parameters and residual electron density maps indicate a well-refined model. Furthermore, the geometrical parameters, including C-C bond lengths and angles, are chemically reasonable and comparable to those found in related structures deposited in the CSD. Therefore, no additional refinement was deemed necessary, and the current model reliably reflects the molecular structure.**

## Alert level G

PLAT083_ALERT_2_G SHELXL Second Parameter in WGHT Unusually Large 10.35 Why ?
PLAT380_ALERT_4_G Incorrectly? Oriented X(sp2)-Methyl Moiety ..... C21 Check
PLAT910_ALERT_3_G Missing FCF Reflection(s) Below Theta(Min)[Deg]= 2.81 Note
0 4 0, 0 8 0,
PLAT912_ALERT_4_G Missing # of FCF Reflections Above STh/L= 0.600 17 Note
PLAT933_ALERT_2_G Number of HKL-OMIT Records in Embedded .res File 1 Note
0 8 0,
PLAT969_ALERT_5_G The 'Henn et al.' R-Factor-gap value ........... 7.047 Note
Predicted wR2: Based on SigI**2 1.80 or SHELX Weight 11.20
PLAT978_ALERT_2_G Number C-C Bonds with Positive Residual Density. 14 Info

0 **ALERT level A** = Most likely a serious problem - resolve or explain
1 **ALERT level B** = A potentially serious problem, consider carefully
1 **ALERT level C** = Check. Ensure it is not caused by an omission or oversight
7 **ALERT level G** = General information/check it is not something unexpected

0 ALERT type 1 CIF construction/syntax error, inconsistent or missing data
4 ALERT type 2 Indicator that the structure model may be wrong or deficient
2 ALERT type 3 Indicator that the structure quality may be low
2 ALERT type 4 Improvement, methodology, query or suggestion
1 ALERT type 5 Informative message, check

# Datablock: 2b

Bond precision: C-C = 0.0033 A Wavelength=0.71073

Cell: a=11.8730(4) b=14.6922(5) c=18.6907(6)
alpha=90 beta=106.4303(13) gamma=90

Temperature: 193 K

| | Calculated | Reported |
|---|---|---|
| Volume | 3127.28(18) | 3127.27(18) |
| Space group | P 21/n | P 1 21/n 1 |
| Hall group | -P 2yn | -P 2yn |
| Moiety formula | C34 H33 Br Cu N2 O2 P | C34 H33 Br Cu N2 O2 P |
| Sum formula | C34 H33 Br Cu N2 O2 P | C34 H33 Br Cu N2 O2 P |
| Mr | 676.04 | 676.04 |
| Dx,g cm-3 | 1.436 | 1.436 |
| Z | 4 | 4 |
| Mu (mm-1) | 2.060 | 2.060 |
| F000 | 1384.0 | 1384.0 |
| F000' | 1384.95 | |
| h,k,lmax | 15,19,24 | 15,19,24 |
| Nref | 7783 | 7765 |
| Tmin,Tmax | 0.586,0.719 | 0.649,0.746 |
| Tmin' | 0.556 | |

Correction method= # Reported T Limits: Tmin=0.649 Tmax=0.746
AbsCorr = MULTI-SCAN

Data completeness= 0.998 Theta(max)= 28.298

R(reflections)= 0.0274( 6397) wR2(reflections)= 0.0743( 7765)

S = 1.102 Npar= 376

The following ALERTS were generated. Each ALERT has the format
**test-name_ALERT_alert-type_alert-level**.
Click on the hyperlinks for more details of the test.

## Alert level C

PLAT905_ALERT_3_C Negative K value in the Analysis of Variance ... -0.577 Report
PLAT911_ALERT_3_C Missing FCF Refl Between Thmin & STh/L= 0.600 14 Report
0 2 0, 1 0 1, -1 1 1, 1 1 1, 1 4 1, -2 0 2,
0 1 2, -1 2 2, 0 3 2, -1 0 3, -1 1 3, 2 1 3,
0 0 4, 0 1 5,
PLAT913_ALERT_3_C Missing # of Very Strong Reflections in FCF .... 4 Note
1 1 1, 0 1 2, 2 1 3, 0 1 5,

## Alert level G

PLAT232_ALERT_2_G Hirshfeld Test Diff (M-X) Cu1 --P1 . 13.0 s.u.
PLAT232_ALERT_2_G Hirshfeld Test Diff (M-X) Cu1 --N2 . 9.3 s.u.
PLAT883_ALERT_1_G Absent Datum for _atom_sites_solution_primary .. Please Do !
PLAT910_ALERT_3_G Missing FCF Reflection(s) Below Theta(Min)[Deg]= 2.26 Note
-1 0 1, 0 1 1,
PLAT912_ALERT_4_G Missing # of FCF Reflections Above STh/L= 0.600 2 Note
PLAT969_ALERT_5_G The 'Henn et al.' R-Factor-gap value ........... 5.188 Note
Predicted wR2: Based on SigI**2 1.43 or SHELX Weight 6.74
PLAT978_ALERT_2_G Number C-C Bonds with Positive Residual Density. 11 Info

0 **ALERT level A** = Most likely a serious problem - resolve or explain
0 **ALERT level B** = A potentially serious problem, consider carefully
3 **ALERT level C** = Check. Ensure it is not caused by an omission or oversight
7 **ALERT level G** = General information/check it is not something unexpected

1 ALERT type 1 CIF construction/syntax error, inconsistent or missing data
3 ALERT type 2 Indicator that the structure model may be wrong or deficient
4 ALERT type 3 Indicator that the structure quality may be low
1 ALERT type 4 Improvement, methodology, query or suggestion
1 ALERT type 5 Informative message, check

# Datablock: 2c

Bond precision: C-C = 0.0056 A Wavelength=0.71073

| | | | |
|---|---|---|---|
| Cell: | a=8.5332(4) | b=11.4089(6) | c=16.0520(9) |
| | alpha=84.663(3) | beta=85.321(3) | gamma=72.587(3) |

Temperature: 193 K

| | Calculated | Reported |
|---|---|---|
| Volume | 1482.28(14) | 1482.28(14) |
| Space group | P -1 | P -1 |
| Hall group | -P 1 | -P 1 |
| Moiety formula | C32 H31 Br Cu N4 P | C32 H31 Br Cu N4 P |
| Sum formula | C32 H31 Br Cu N4 P | C32 H31 Br Cu N4 P |
| Mr | 646.03 | 646.03 |
| Dx,g cm-3 | 1.447 | 1.447 |
| Z | 2 | 2 |
| Mu (mm-1) | 2.166 | 2.166 |
| F000 | 660.0 | 660.0 |
| F000' | 660.43 | |
| h,k,lmax | 10,13,19 | 10,13,19 |
| Nref | 5375 | 5341 |
| Tmin,Tmax | 0.771,0.897 | 0.591,0.745 |
| Tmin' | 0.648 | |

Correction method= # Reported T Limits: Tmin=0.591 Tmax=0.745
AbsCorr = MULTI-SCAN

Data completeness= 0.994 Theta(max)= 25.249

R(reflections)= 0.0418( 3963) wR2(reflections)= 0.1096( 5341)

S = 0.957 Npar= 362

The following ALERTS were generated. Each ALERT has the format
**test-name_ALERT_alert-type_alert-level**.
Click on the hyperlinks for more details of the test.

## Alert level C

PLAT420_ALERT_2_C D-H Bond Without Acceptor N2 --H2N . Please Check
PLAT906_ALERT_3_C Large K Value in the Analysis of Variance ...... 3.916 Check
PLAT911_ALERT_3_C Missing FCF Refl Between Thmin & STh/L= 0.600 31 Report
1 1 0, -4 2 0, -5 3 0, 5 -3 1, -1 -1 1, 1 1 1,
-6 3 1, -5 3 1, -2 7 1, 0 -1 2, 0 0 2, 0 1 2,

```
-5  3  2,  -2  6  2,  -2  7  2,   0  0  3,  -2  8  4,  -8  4  5,
-7  4  5,   5 -8  6,  -7  4  6,  -7  5  6,  -7  5  7,  -5  3  8,
-7  3  9,  -6  3  9,  -5  3  9,  -7  3 10,  -7  2 11,  -6  2 11,
  (   1 More Missing: see the .ckf listing file)
```

## Alert level G

PLAT002_ALERT_2_G Number of Distance or Angle Restraints on AtSite 4 Note
PLAT154_ALERT_1_G The s.u.'s on the Cell Angles are Equal ..(Note) 0.003 Degree
PLAT172_ALERT_4_G The CIF-Embedded .res File Contains DFIX Records 1 Report
PLAT860_ALERT_3_G Number of Least-Squares Restraints ............ 2 Note
PLAT883_ALERT_1_G Absent Datum for _atom_sites_solution_primary .. Please Do !
PLAT909_ALERT_3_G Percentage of I>2sig(I) Data at Theta(Max) Still 53% Note
PLAT910_ALERT_3_G Missing FCF Reflection(s) Below Theta(Min)[Deg]= 2.34 Note
0 1 0, 0 0 1, 0 1 1,
PLAT933_ALERT_2_G Number of HKL-OMIT Records in Embedded .res File 2 Note
0 0 1, 0 0 3,
PLAT941_ALERT_3_G Average HKL Measurement Multiplicity .......... 2.3 Low
PLAT961_ALERT_5_G Dataset Contains no Negative Intensities ....... Please Check
PLAT967_ALERT_5_G Note: Two-Theta Cutoff Value in Embedded .res .. 50.5 Degree
PLAT969_ALERT_5_G The 'Henn et al.' R-Factor-gap value ........... 2.635 Note
Predicted wR2: Based on SigI**2 4.16 or SHELX Weight 11.46
PLAT978_ALERT_2_G Number C-C Bonds with Positive Residual Density. 3 Info

0 **ALERT level A** = Most likely a serious problem - resolve or explain
0 **ALERT level B** = A potentially serious problem, consider carefully
3 **ALERT level C** = Check. Ensure it is not caused by an omission or oversight
13 **ALERT level G** = General information/check it is not something unexpected

2 ALERT type 1 CIF construction/syntax error, inconsistent or missing data
4 ALERT type 2 Indicator that the structure model may be wrong or deficient
6 ALERT type 3 Indicator that the structure quality may be low
1 ALERT type 4 Improvement, methodology, query or suggestion
3 ALERT type 5 Informative message, check

# Datablock: 3b

Bond precision: C-C = 0.0170 A Wavelength=0.71073

Cell: a=13.9213(14) b=15.2466(13) c=37.608(3)
alpha=90 beta=98.088(5) gamma=90

Temperature: 193 K

| | Calculated | Reported |
|---|---|---|
| Volume | 7903.0(12) | 7903.1(12) |
| Space group | P 21/n | P 1 21/n 1 |
| Hall group | -P 2yn | -P 2yn |
| Moiety formula | 4(C36 H36 Cu N3 O2 P), 4(F6 P), 2(C H Cl3), C H2 Cl2 | 4(C36 H36 Cu N3 O2 P), 4(F6 P), 2(C H Cl3), C H2 Cl2 |
| Sum formula | C147 H148 Cl8 Cu4 F24 N12 O8 P8 | C147 H148 Cl8 Cu4 F24 N12 O8 P8 |
| Mr | 3452.33 | 3452.29 |
| Dx,g cm-3 | 1.451 | 1.451 |
| Z | 2 | 2 |
| Mu (mm-1) | 0.834 | 0.834 |
| F000 | 3532.0 | 3532.0 |
| F000' | 3540.17 | |
| h,k,lmax | 17,19,46 | 17,18,46 |
| Nref | 16056 | 14503 |
| Tmin,Tmax | 0.951,0.975 | 0.620,0.745 |
| Tmin' | 0.920 | |

Correction method= # Reported T Limits: Tmin=0.620 Tmax=0.745
AbsCorr = MULTI-SCAN

Data completeness= 0.903 Theta(max)= 26.311

R(reflections)= 0.1179( 5983) wR2(reflections)= 0.3508( 14503)

S = 1.078 Npar= 979

The following ALERTS were generated. Each ALERT has the format
**test-name_ALERT_alert-type_alert-level**.
Click on the hyperlinks for more details of the test.

## Alert level A

PLAT029_ALERT_3_A _diffrn_measured_fraction_theta_full value Low . 0.933 Why?

**Author Response: The crystal was of limited quality, even though it was the best specimen selected from the supplied sample, which consisted only of very small crystals. As a result, high-angle theta diffraction is either not observed or extremely weak.**

## Alert level B

PLAT341_ALERT_3_B Low Bond Precision on C-C Bonds .............. 0.01703 Ang.

**Author Response: Please see our response to Alert _vrf_PLAT029.**

PLAT911_ALERT_3_B Missing FCF Refl Between Thmin & STh/L= 0.600 954 Report
1 1 0, 0 2 0, 5 14 0, 7 14 0, 8 14 0, 5 15 0,
7 15 0, 8 15 0, 9 15 0, 6 16 0, 7 16 0, 8 16 0,
0 18 0, 1 0 1, 1 1 1, -12 12 1, -10 12 1, -11 13 1,
-10 14 1, -8 14 1, -7 14 1, 4 14 1, 8 14 1, -9 15 1,
-7 15 1, -6 15 1, 5 15 1, 6 15 1, 7 15 1, 9 15 1,
( 924 More Missing: see the .ckf listing file)

**Author Response: Please see our response to Alert _vrf_PLAT029.**

## Alert level C

RINTA01_ALERT_3_C The value of Rint is greater than 0.12
Rint given 0.175
PLAT020_ALERT_3_C The Value of Rint is Greater Than 0.12 ......... 0.175 Report
PLAT026_ALERT_3_C Ratio Observed / Unique Reflections (too) Low .. 41% Check
PLAT082_ALERT_2_C High R1 Value ................................ 0.12 Report
PLAT084_ALERT_3_C High wR2 Value (i.e. > 0.25) .................. 0.35 Report
PLAT234_ALERT_4_C Large Hirshfeld Difference N2 --C29 . 0.18 Ang.
**And 14 other PLAT234 Alerts**
More ...
PLAT241_ALERT_2_C High 'MainMol' Ueq as Compared to Neighbors of C40 Check
PLAT242_ALERT_2_C Low 'MainMol' Ueq as Compared to Neighbors of C71 Check
PLAT243_ALERT_4_C High 'Solvent' Ueq as Compared to Neighbors of C73 Check
PLAT260_ALERT_2_C Large Average Ueq of Residue Including P4 0.177 Check
**And 2 other PLAT260 Alerts**
More ...
PLAT369_ALERT_2_C Long C(sp2)-C(sp2) Bond C38 - C51 . 1.53 Ang.
PLAT369_ALERT_2_C Long C(sp2)-C(sp2) Bond C42 - C43 . 1.53 Ang.
PLAT906_ALERT_3_C Large K Value in the Analysis of Variance ...... 15.521 Check
**And 2 other PLAT906 Alerts**
More ...

## Alert level G

PLAT002_ALERT_2_G Number of Distance or Angle Restraints on AtSite 7 Note
PLAT003_ALERT_2_G Number of Uiso or U(i,j) Restrained non-H-Atoms 29 Report
PLAT072_ALERT_2_G SHELXL First Parameter in WGHT Unusually Large 0.17 Report
PLAT172_ALERT_4_G The CIF-Embedded .res File Contains DFIX Records 4 Report
PLAT177_ALERT_4_G The CIF-Embedded .res File Contains DELU Records 5 Report
PLAT178_ALERT_4_G The CIF-Embedded .res File Contains SIMU Records 5 Report
PLAT186_ALERT_4_G The CIF-Embedded .res File Contains ISOR Records 5 Report
PLAT188_ALERT_3_G A Non-default SIMU Restraint Value has been used 0.0200 Report
**And 4 other PLAT188 Alerts**
More ...
PLAT192_ALERT_3_G A Non-default DELU Restraint Value for First Par 0.0200 Report
**And 4 other PLAT192 Alerts**
More ...
PLAT244_ALERT_4_G Low 'Solvent' Ueq as Compared to Neighbors of P3 Check
PLAT244_ALERT_4_G Low 'Solvent' Ueq as Compared to Neighbors of P4 Check
PLAT299_ALERT_4_G Atom Site Occupancy Constrained at ............ 0.5 Check
Cl4 Cl5 C74 H74A H74B
PLAT302_ALERT_4_G Anion/Solvent/Minor-Residue Disorder (Resd 6) 100% Note
PLAT304_ALERT_4_G Non-Integer Number of Atoms in ..... (Resd 6) 2.50 Check
PLAT380_ALERT_4_G Incorrectly? Oriented X(sp2)-Methyl Moiety ..... C13 Check
PLAT380_ALERT_4_G Incorrectly? Oriented X(sp2)-Methyl Moiety ..... C21 Check
PLAT432_ALERT_2_G Short Inter X...Y Contact Cl5 ..C72 . 3.12 Ang.
x,-1+y,z = 1_545 Check
PLAT432_ALERT_2_G Short Inter X...Y Contact Cl5 ..C71 . 3.18 Ang.
x,-1+y,z = 1_545 Check
PLAT434_ALERT_2_G Short Inter HL..HL Contact Cl3 ..Cl5 . 3.22 Ang.
x,y,z = 1_555 Check
PLAT789_ALERT_4_G Atoms with Negative _atom_site_disorder_group # 5 Check
PLAT822_ALERT_4_G CIF-embedded .res Contains Negative PART Numbers 1 Check
PLAT860_ALERT_3_G Number of Least-Squares Restraints ............ 183 Note
PLAT883_ALERT_1_G Absent Datum for _atom_sites_solution_primary .. Please Do !

PLAT910_ALERT_3_G Missing FCF Reflection(s) Below Theta(Min)[Deg]= 1.50 Note
0 1 1, 0 0 2,
PLAT912_ALERT_4_G Missing # of FCF Reflections Above STh/L= 0.600 596 Note
PLAT933_ALERT_2_G Number of HKL-OMIT Records in Embedded .res File 5 Note
1 0 1, 0 1 1, 0 1 2, 0 0 2, 1 0 3,
PLAT941_ALERT_3_G Average HKL Measurement Multiplicity ........... 4.7 Low
PLAT969_ALERT_5_G The 'Henn et al.' R-Factor-gap value ........... 5.004 Note
Predicted wR2: Based on SigI**2 7.01 or SHELX Weight 32.55
PLAT978_ALERT_2_G Number C-C Bonds with Positive Residual Density. 0 Info

---

1 **ALERT level A** = Most likely a serious problem - resolve or explain
2 **ALERT level B** = A potentially serious problem, consider carefully
31 **ALERT level C** = Check. Ensure it is not caused by an omission or oversight
37 **ALERT level G** = General information/check it is not something unexpected

1 ALERT type 1 CIF construction/syntax error, inconsistent or missing data
16 ALERT type 2 Indicator that the structure model may be wrong or deficient
23 ALERT type 3 Indicator that the structure quality may be low
30 ALERT type 4 Improvement, methodology, query or suggestion
1 ALERT type 5 Informative message, check

---

# Datablock: 4

| | | | |
|---|---|---|---|
| Bond precision: | C-C = 0.0041 A | | Wavelength=0.71073 |
| Cell: | a=17.7749(10) | b=15.9796(9) | c=22.5680(14) |
| | alpha=90 | beta=96.691(2) | gamma=90 |
| Temperature: | 193 K | | |

| | Calculated | Reported |
|---|---|---|
| Volume | 6366.5(6) | 6366.5(6) |
| Space group | P 21/c | P 1 21/c 1 |
| Hall group | -P 2ybc | -P 2ybc |
| Moiety formula | C64 H62 Cl Cu2 N8 P2, B F4, C H Cl3 | C64 H62 Cl Cu2 N8 P2, B F4, C H Cl3 |
| Sum formula | C65 H63 B Cl4 Cu2 F4 N8 P2 | C65 H63 B Cl4 Cu2 F4 N8 P2 |
| Mr | 1373.89 | 1373.86 |
| Dx,g cm-3 | 1.433 | 1.433 |
| Z | 4 | 4 |
| Mu (mm-1) | 0.945 | 0.945 |
| F000 | 2824.0 | 2824.0 |
| F000' | 2830.60 | |
| h,k,lmax | 21,19,27 | 21,19,27 |
| Nref | 11531 | 11515 |
| Tmin,Tmax | 0.753,0.828 | 0.603,0.746 |
| Tmin' | 0.654 | |

Correction method= # Reported T Limits: Tmin=0.603 Tmax=0.746
AbsCorr = MULTI-SCAN

Data completeness= 0.999 Theta(max)= 25.249

R(reflections)= 0.0359( 9283) wR2(reflections)= 0.0949( 11515)

S = 1.039 Npar= 820

---

The following ALERTS were generated. Each ALERT has the format
**test-name_ALERT_alert-type_alert-level**.
Click on the hyperlinks for more details of the test.

---

## Alert level C

PLAT244_ALERT_4_C Low 'Solvent' Ueq as Compared to Neighbors of B1 Check
PLAT244_ALERT_4_C Low 'Solvent' Ueq as Compared to Neighbors of C65 Check
PLAT420_ALERT_2_C D-H Bond Without Acceptor N4 --H4N . Please Check
PLAT420_ALERT_2_C D-H Bond Without Acceptor N8 --H8N . Please Check
PLAT910_ALERT_3_C Missing FCF Reflection(s) Below Theta(Min)[Deg]= 2.40 Note
1 0 0, 1 1 0, 2 0 0, -1 1 1, 0 1 1, 1 1 1,
-1 0 2, 0 0 2, 0 1 2, 1 0 2,
PLAT911_ALERT_3_C Missing FCF Refl Between Thmin & STh/L= 0.600 7 Report
1 2 1, -4 0 4, 0 0 4, 1 0 6, 5 0 14, 7 0 16,
10 0 18,

---

## Alert level G

PLAT002_ALERT_2_G Number of Distance or Angle Restraints on AtSite 9 Note
PLAT003_ALERT_2_G Number of Uiso or U(i,j) Restrained non-H-Atoms 12 Report

PLAT007_ALERT_5_G Number of Unrefined Donor-H Atoms ............. 4 Report
H2N H4N H6N H8N
PLAT172_ALERT_4_G The CIF-Embedded .res File Contains DFIX Records 2 Report
PLAT176_ALERT_4_G The CIF-Embedded .res File Contains SADI Records 2 Report
PLAT177_ALERT_4_G The CIF-Embedded .res File Contains DELU Records 2 Report
PLAT178_ALERT_4_G The CIF-Embedded .res File Contains SIMU Records 2 Report
PLAT186_ALERT_4_G The CIF-Embedded .res File Contains ISOR Records 2 Report
PLAT188_ALERT_3_G A Non-default SIMU Restraint Value has been used 0.0090 Report
PLAT188_ALERT_3_G A Non-default SIMU Restraint Value has been used 0.0090 Report
PLAT191_ALERT_3_G A Non-default SADI Restraint Value has been used 0.0090 Report
PLAT191_ALERT_3_G A Non-default SADI Restraint Value has been used 0.0090 Report
PLAT192_ALERT_3_G A Non-default DELU Restraint Value for First Par 0.0090 Report
PLAT192_ALERT_3_G A Non-default DELU Restraint Value for First Par 0.0090 Report
PLAT231_ALERT_4_G Hirshfeld Test (Solvent) F4A --B1 . 5.5 s.u.
PLAT232_ALERT_2_G Hirshfeld Test Diff (M-X) Cu1 --P1 . 9.3 s.u.
PLAT232_ALERT_2_G Hirshfeld Test Diff (M-X) Cu2 --P2 . 6.0 s.u.
PLAT300_ALERT_4_G Atom Site Occupancy of F1A Constrained at 0.8 Check
**And 7 other PLAT300 Alerts**
More ...
PLAT302_ALERT_4_G Anion/Solvent/Minor-Residue Disorder (Resd 2) 80% Note
PLAT432_ALERT_2_G Short Inter X...Y Contact F4B ..C62 . 2.95 Ang.
2-x,1/2+y,1/2-z = 2_755 Check
PLAT793_ALERT_4_G Model has Chirality at N2 (Centro SpGr) R Verify
PLAT860_ALERT_3_G Number of Least-Squares Restraints ............ 152 Note
PLAT883_ALERT_1_G Absent Datum for _atom_sites_solution_primary .. Please Do !
PLAT909_ALERT_3_G Percentage of I>2sig(I) Data at Theta(Max) Still 64% Note
PLAT913_ALERT_3_G Missing # of Very Strong Reflections in FCF .... 3 Note
-1 0 2, 1 0 2, -4 0 4,
PLAT933_ALERT_2_G Number of HKL-OMIT Records in Embedded .res File 5 Note
1 0 0, 1 1 0, 0 1 1, -1 1 1, 1 0 2,
PLAT961_ALERT_5_G Dataset Contains no Negative Intensities ....... Please Check
PLAT967_ALERT_5_G Note: Two-Theta Cutoff Value in Embedded .res .. 50.5 Degree
PLAT969_ALERT_5_G The 'Henn et al.' R-Factor-gap value ........... 3.609 Note
Predicted wR2: Based on SigI**2 2.63 or SHELX Weight 9.14
PLAT978_ALERT_2_G Number C-C Bonds with Positive Residual Density. 2 Info

---

0 **ALERT level A** = Most likely a serious problem - resolve or explain
0 **ALERT level B** = A potentially serious problem, consider carefully
6 **ALERT level C** = Check. Ensure it is not caused by an omission or oversight
37 **ALERT level G** = General information/check it is not something unexpected

1 ALERT type 1 CIF construction/syntax error, inconsistent or missing data
9 ALERT type 2 Indicator that the structure model may be wrong or deficient
11 ALERT type 3 Indicator that the structure quality may be low
18 ALERT type 4 Improvement, methodology, query or suggestion
4 ALERT type 5 Informative message, check

---

It is advisable to attempt to resolve as many as possible of the alerts in all categories. Often the minor alerts point to easily fixed oversights, errors and omissions in your CIF or refinement strategy, so attention to these fine details can be worthwhile. In order to resolve some of the more serious problems it may be necessary to carry out additional measurements or structure refinements. However, the purpose of your study may justify the reported deviations and the more serious of these should normally be commented upon in the discussion or experimental section of a paper or in the "special_details" fields of the CIF. checkCIF was carefully designed to identify outliers and unusual parameters, but every test has its limitations and alerts that are not important in a particular case may appear. Conversely, the absence of alerts does not guarantee there are no aspects of the results needing attention. It is up to the individual to critically assess their own results and, if necessary, seek expert advice.

**Publication of your CIF in IUCr journals**

A basic structural check has been run on your CIF. These basic checks will be run on all CIFs submitted for publication in IUCr journals (*Acta Crystallographica*, *Journal of Applied Crystallography*, *Journal of Synchrotron Radiation*); however, if you intend to submit to *Acta Crystallographica Section C* or *E* or *IUCrData*, you should make sure that full publication checks are run on the final version of your CIF prior to submission.

**Publication of your CIF in other journals**

Please refer to the *Notes for Authors* of the relevant journal for any special instructions relating to CIF submission.

---

**PLATON version of 04/06/2025; check.def file version of 30/05/2025**

# **Datablock 1** - ellipsoid plot

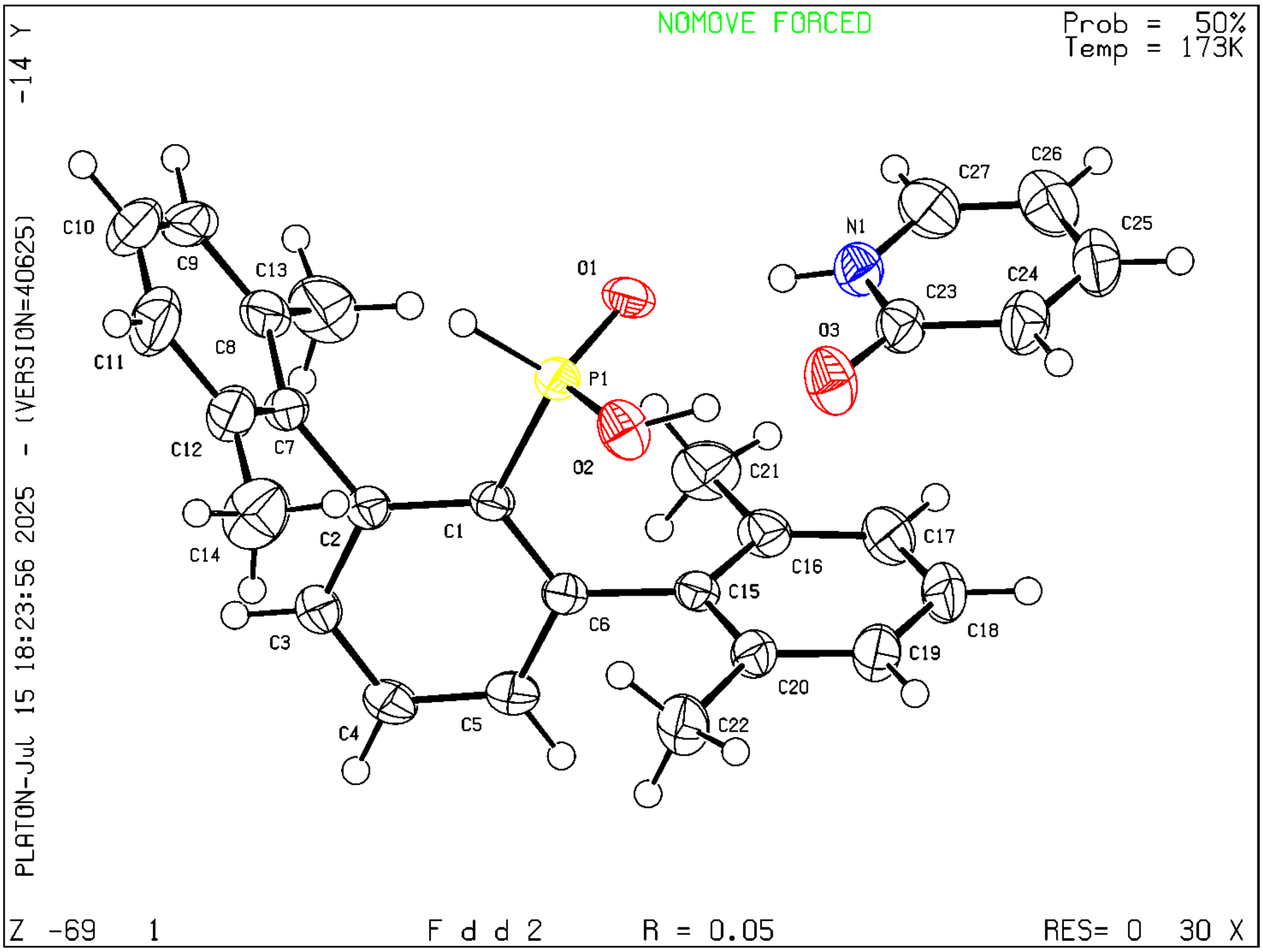


# **Datablock 2b** - ellipsoid plot

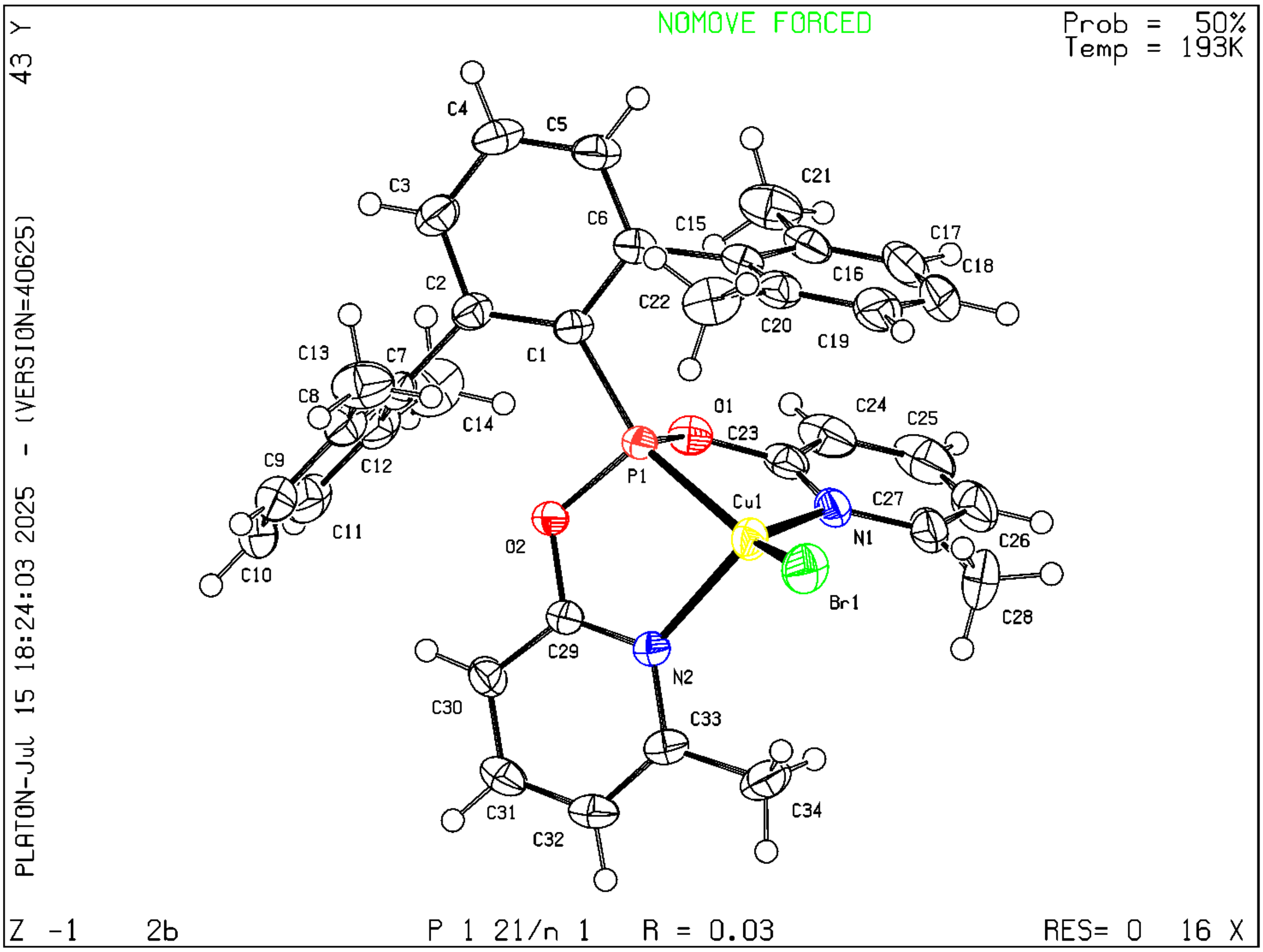


# **Datablock 2c** - ellipsoid plot

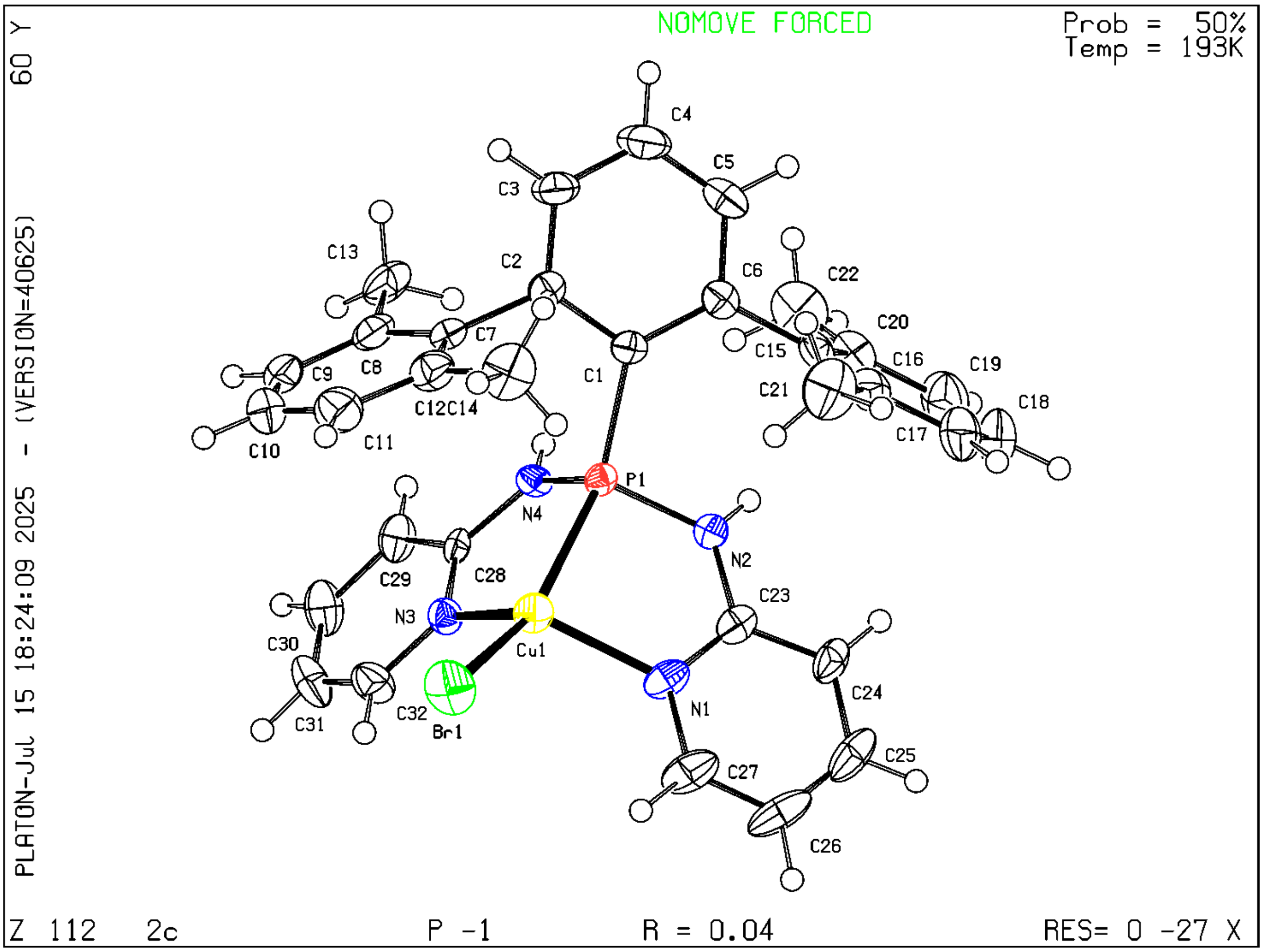


# **Datablock 3b** - ellipsoid plot

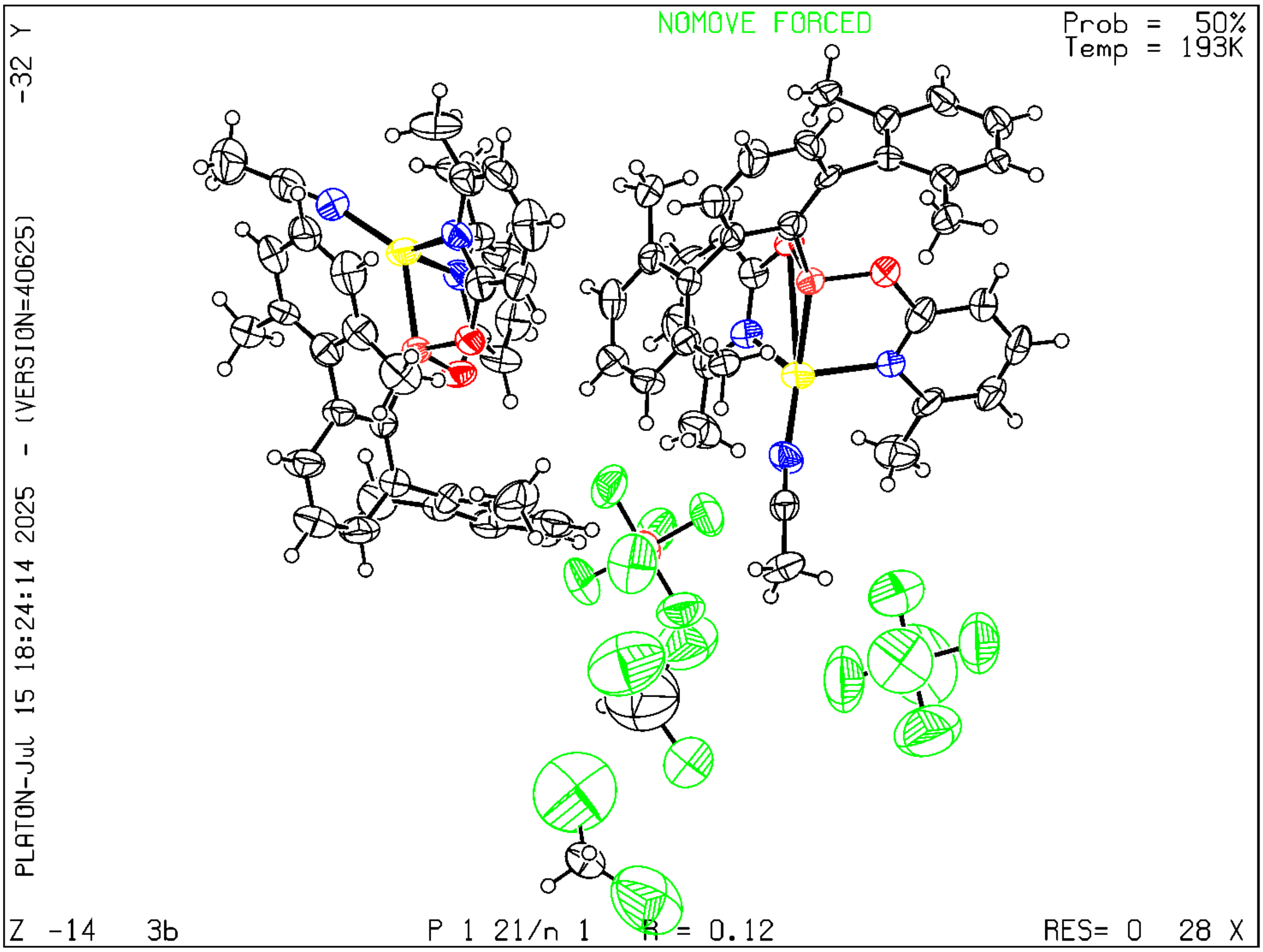


## **Datablock 4** - ellipsoid plot

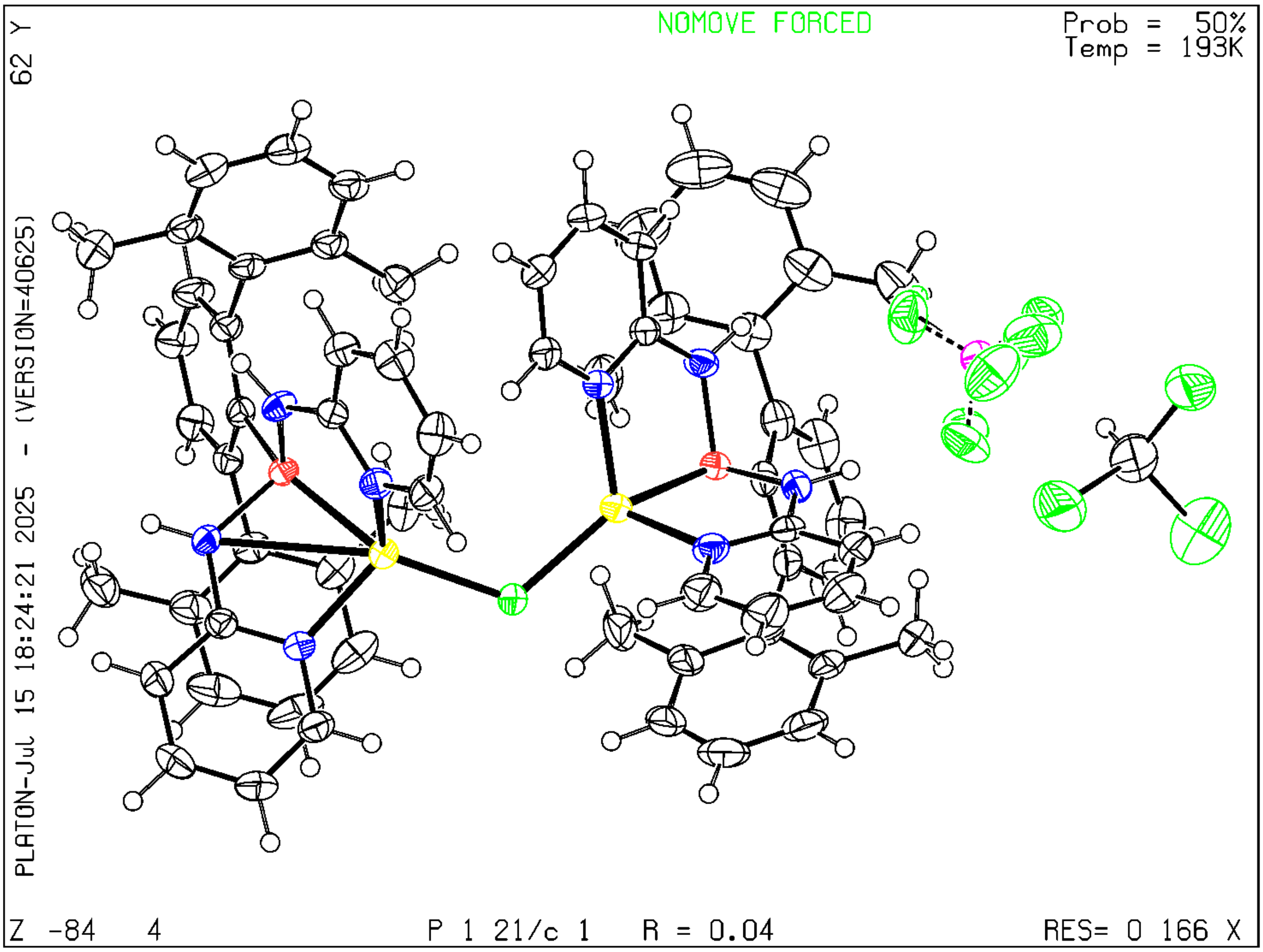


---

Download CIF editor (publCIF) from the IUCr
Download CIF editor (enCIFer) from the CCDC
Test a new CIF entry
