# Supplementary material for: Copper(I) Complexes with Terphenyl-Substituted NPN Ligands Bearing Pyridyl Groups: Synthesis, Characterization, and Catalytic Studies in the S-Arylation of Thiols
Source: Molecules. 2025 Jul 29;30(15):3167. doi: 10.3390/molecules30153167 (PMC12348607; doi:10.3390/molecules30153167)
Supplement: Supplementary file 1 [file molecules-30-03167-s001.zip › molecules-3759414-supplementary/Supplementary Materials.pdf]

Supplementary Information

for

Copper(I) complexes with terphenyl-substituted NPN ligands bearing pyridyl groups: synthesis, characterization, and catalytic studies in the S-arylation of thiols.

M. Trinidad Martín,[1,*] Ana Gálvez del Postigo,[1] Práxedes Sánchez,[1] Eleuterio Álvarez,[1] Celia Maya,[1] M. Carmen Nicasio,[2,*] and Riccardo Peloso[1,*]

1 Instituto de Investigaciones Químicas (IIQ), Departamento de Química Inorgánica and Centro de Innovación en Química Avanzada (ORFEO−CINQA), Consejo Superior de Investigaciones Científicas (CSIC) and Universidad de Sevilla, 41092 Sevilla, Spain.

2 Departamento de Química Inorgánica, Universidad de Sevilla, 41071, Sevilla, Spain.

* Correspondence: rpeloso@us.es

1. **Synthesis of ligands and metal complexes** **p. 1**

2. **General catalytic procedure for the S-arylation of thiols with aryl iodides** **p. 11**

3. **Characterization data for thioethers** **p. 11**

4. **NMR spectra** **p. 17**

5. **Single Crystal X-ray diffraction analyses** **p. 48**

1. Synthesis of ligands and metal complexes

NOPON^{Xyl_2}, di(pyridin-2-yl)(2,6-bis(2,6-dimethylphenyl)phenyl)phosphonite

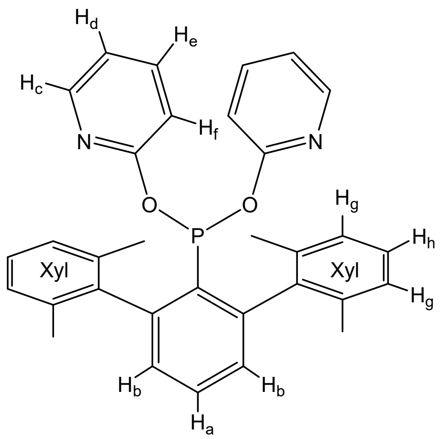

A solution of 2-hydroxypyridine (0.66 g, 6.9 mmol) in 5 mL of THF was added to a solution of $PX_2Ar^{Xyl_2}$ [1] (1.50 g, 3.6 mmol) in 5 mL of diethylether. The resulting mixture was cooled to 0 °C and triethylamine (2.10 mL, 15.4 mmol) was added. The suspension was stirred for 24 h at room temperature and filtered. The resulting pale yellow solution was taken to dryness under reduced pressure to give a solid residue which was washed with pentane, filtered, dried under vacuum, and identified as **NOPON^{Xyl_2}** (1.40 g, 80%). In some preparations the ^{1}H NMR analyses of the product revealed the presence of variable amounts of 2-hydroxypyridine, which could easily be removed by washing with ether at -20 °C.

^{1}H RMN ($CDCl_3$, 300 MHz, 298 K): δ 7.87 (dd, 2H, $^4J_{HP}$ = 2.1 Hz, $^3J_{HH}$ = 7.5 Hz, H_c), 7.56 (t, 1H, $^3J_{HH}$ = 7.4 Hz, H_a), 7.38 (ddd, 2H, $^4J_{HP}$ = 2.1 Hz, $^3J_{HH}$ = 7.4 Hz, H_e), 7.09 (dd, 2H, $^4J_{HP}$ = 2.4 Hz, $^3J_{HH}$ = 7.6 Hz, H_b), 7.03 (t, 2H, $^3J_{HH}$ = 7.4 Hz, H_p), 6.95 (d, 4H, $^3J_{HH}$ = 7.6 Hz, H_m), 6.76 (ddd, 2H, $^5J_{HP}$ = 0.8 Hz, $^3J_{HH}$ = 6.5 Hz, H_d), 6.23 (d, 2H, $^3J_{HH}$ = 8.2 Hz, H_f), 2.11 (s, 12 H, Xyl) ppm.

^{31}P{^{1}H} RMN ($CDCl_3$, 121 MHz, 298 K): δ 149.3 (s) ppm.

^{13}C RMN ($CDCl_3$, 75 MHz, 298 K): δ 161.4 (d, $^2J_{CP}$ = 9 Hz, C-O), 146.9 (d, $^3J_{CP}$ = 2 Hz, C-H_c), 145.7 (d, $^2J_{CP}$ = 22 Hz, C_{orto}-central ring), 141.3 (d, $^3J_{CP}$ = 5 Hz, C_{ipso} -Xyl), 138.4 (C-H_e), 136.4 (d, $^4J_{CP}$ = 2 Hz, *C*-CH_3), 135.7 (d, $^1J_{CP}$ = 28 Hz, C-P), 131.1 (C-H_a), 129.4 (d, $^3J_{CP}$ = 2 Hz , C-H_b), 127.0 (C-H_p), 126.9 (C-H_m), 117.8 (C-H_d), 112 (d, $^3J_{CP}$ = 2 Hz, C-CH_f), 21.2 (CH_3) ppm.

Elemental analysis calculated (found) for $C_{32}H_{29}N_2O_2P$: C, 76.2 (76.3); H, 5.8 (5.8); N, 5.6 (5.4).

---

[1] PX_2Ar^{Xyl} is a mixture of the three diahalophopshines containing Br and Cl, namely PCl_2 Ar^{Xyl2}, PBr_2 Ar^{Xyl}, and $PBrClAr^{Xyl}$, and is prepared as described in ref. 33 (see Article).

NOPON$^{Xyl_2\text{-}Me_2}$, bis(6-methylpyridin-2-yl) (2,6-bis(2,6-dimethylphenyl)phenyl) phosphonite

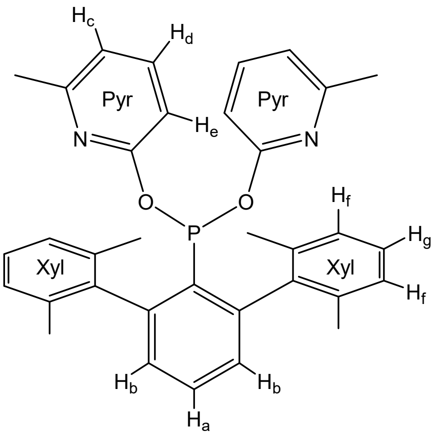

The compound was prepared following the same procedure described for **NOPON^{Xyl_2}** using hydroxy-6-methylpyridine instead of 2-hydroxypyridine. Yield: 70-80%.

^{1}H RMN ($CDCl_3$, 300 MHz, 298 K): δ 7.56 (t, 1H, $^3J_{HH}$ = 7.6 Hz, H$_a$), 7.26 (t, 2H, $^3J_{HH}$ = 7.7 Hz, H$_e$), 7.11 (dd, 2H, $^3J_{HH}$ = 7.9 Hz, $^4J_{HP}$ = 2.1Hz, H$_b$), 7.06 (d, 2H, $^3J_{HH}$ = 6.8 Hz, H$_p$), 6.98 (d, 4H, $^3J_{HH}$ = 7.3 Hz, H$_m$), 6.62 (dd, 2H, $^3J_{HH}$ = 7.5 Hz, $^5J_{HP}$ =0.9Hz, H$_d$), 6.04 (d, 2H, $^3J_{HH}$ = 7.6 Hz, H$_f$), 2.15 (s, 12H, CH_3 Xyl), 2.11 (s, 6H, CH_3 Pyr) ppm.

^{31}P{^{1}H} RMN ($CDCl_3$, 121 MHz, 298 K): δ 150.0 (s) ppm.

^{13}C RMN ($CDCl_3$, 75 MHz, 298 K): δ 160.0 (d, $^2J_{CP}$ = 9 Hz, C-O), 155.0 (d, $^4J_{CP}$ = 1 Hz, C-CH_3 Pyr), 144.5 (d, $^2J_{CP}$ = 22 Hz, C$_{orto}$-central ring), 140.6 (d, $^3J_{CP}$ = 5 Hz, C$_{ipso}$-Xyl), 137.3 (C-H$_e$), 135.4 (d, $^4J_{CP}$ = 2Hz, C-CH_3 Xyl), 134.1 (d, $^1J_{CP}$ = 27Hz, C-P), 129.8 (C-H$_a$), 128.4 (d, $^3J_{CP}$ = 2Hz ,C-H$_b$), 126.2 (C-H$_p$), 125.7 (C-H$_m$), 116.0 (C-H$_d$), 107.6 (d, $^3J_{CP}$ = 4Hz,C-H$_f$), 22.4 (CH_3 Pyr), 20.2 (CH_3 Xyl) ppm.

Elemental analysis calculated (found) for $C_{34}H_{33}N_2O_2P$: C, 76.7 (76.6); H, 6.3 (6.4); N, 5.3 (5.2).

Hydrolisis of ligands NOPON^{Xyl_2} and NOPON$^{Xyl_2\text{-}Me_2}$.

In Solution. Approximately 0.1 mL of water were added to a THF solution (5 mL) of **NOPON^{Xyl_2}** (0.1 mmol). The reaction mixture was stirred for *ca.* 24 h at room temperature, dried over $MgSO_4$, and filtered. The resulting solution was taken to dryness under reduced pressure yielding a solid material which was washed with pentane, dried under vacuum, and identified as a 2 : 1 mixture of 2-hydroxypyridine and (O=)PH(OH)Ar^{Xyl_2} (**1**). The same procedure was employed for **NOPON$^{Xyl_2\text{-}Me_2}$**, although full conversion into **1** and 2-hydroxy-6-methylpyridine was achieved after *ca.* 48 h.

In the solid state. Solid samples of **NOPON^{Xyl_2}** exposed to the air at room temperature converted into the same 2 : 1 mixture of 2-hydroxypyridine and **1** within 4 days. Complete hydrolysis of **NOPON$^{Xyl_2\text{-}Me_2}$** under the same conditions was achieved in approximately one week.

NMR characterization of **1** was performed using the samples obtained as described above.

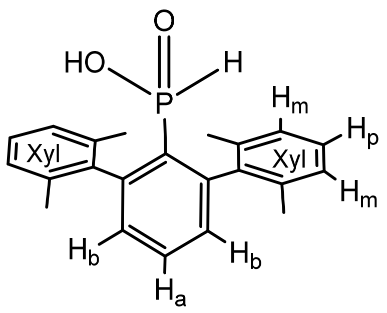

^{1}H RMN ($CDCl_3$, 300 MHz, 298 K): δ 7.79 (s, 1H, P-OH), 7.50 (t, 1H, $^3J_{HH}$ = 7.5 Hz, H_a), 6.99 (dd, 2H, $^3J_{HH}$ = 7.6 Hz, $^4J_{HP}$ = 3.4Hz, H_b), 6.90-6.80 (m, 6H, H_m, H_p), 5.89 (s, 1H, P-H), 2.02 (s, 12H, CH_3 Xyl) ppm. ^{13}C{^{1}H} RMN ($CDCl_3$, 75 MHz, 298K): δ 143.0 (d, $^2J_{CP}$ = 12 Hz, C_{orto} central ring), 138.5 (d, $^3J_{CP}$ = 5 Hz, C_{ipso} Xyl), 135.7 (C-CH_3), 130.7 (C-H_a), 128.0 (d, $^3J_{CP}$ = 8 Hz, C-H_b), 126.3 (C-H_p), 125.7 (C-H_m), 20.1 (CH_3) ppm. ^{31}P{^{1}H} RMN ($CDCl_3$, 121 MHz, 298 K): δ 23.6 (s) ppm.

$N_2PN_2^{Xyl_2}$, N,N'-di(pyridine-2-yl)-1-(2,6-bis(2,6-dimethylphenyl)phenyl) phosphane diamine

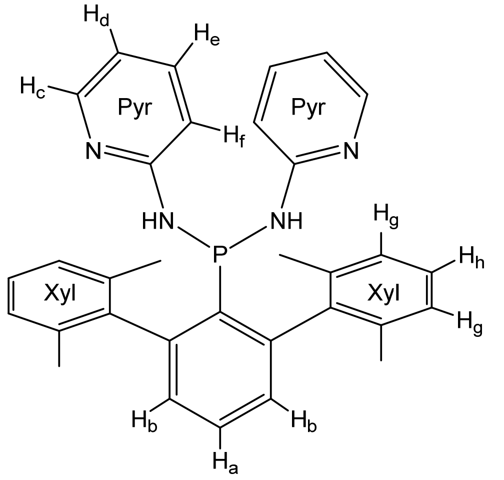

The compound was prepared following the same procedure described for **$NOPON^{Xyl_2}$** using 2-aminopyridine instead of 2-hydroxypyridine. Yield: 70-80%.

^1H RMN ($CDCl_3$, 300 MHz, 298 K): δ 7.87 (d, 2H, $^3J_{HH}$ = 6.4 Hz, H_c), 7.43 (t, 1H, $^3J_{HH}$ = 7.6 Hz, H_a), 7.19-7.11 (m, 4H, H_e, H_b), 7.04-7.01 (m, 4H, $^3J_{HH}$ = 7.4 Hz, H_m), 6.99 (m, 2H, H_p), 6.58 (t, 2H, $^3J_{HH}$ = 7.3 Hz, H_d), 6.25 (d, 2H, $^3J_{HH}$ = 7.6 Hz, H_f), 5.04 (bs, 2H, NH), 1.99 (s, 12 H, CH_3 Xyl) ppm.

$^{31}P\{^1H\}$ RMN ($CDCl_3$, 121 MHz, 298 K): δ 37.8 (s) ppm.

$^{13}C\{^1H\}$ RMN ($CDCl_3$, 75 MHz, 298 K): δ 157.4 (d, $^2J_{CP}$ = 9 Hz, C-O), 147.5 (d, $^3J_{CP}$ = 1Hz, C-H_c), 145.0 (d, $^2J_{CP}$ = 21 Hz, C_{orto}- central ring), 144.8 (d, $^3J_{CP}$ = 5Hz, C_{ipso} -Xyl), 141.2 (C-H_e), 137.1 (d, $^4J_{CP}$ = 1Hz, *C*-CH_3), 136.2 (d, $^1J_{CP}$ = 27Hz, C-P), 130.1 (C-H_a), 129.6 (d, $^3J_{CP}$ = 2.1Hz, C-H_b), 127.7 (C-H_p), 127.6 (C-H_m), 114.6 (C-H_d), 109.3 (d, $^3J_{CP}$ = 2Hz, C-H_f), 21.1 (CH_3) ppm.

Elemental analysis calculated (found) for $C_{32}H_{31}N_4P$: C, 76.5 (76.6); H, 6.2 (6.5); N, 11.2 (10.8).

CuBr(κ^3-*N,P,N*-NOPON^{Xyl2}), 2a

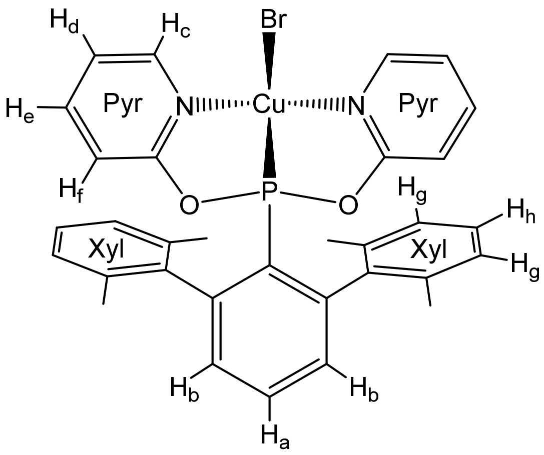

Solid NOPON^{Xyl2} (90 mg, 0.20 mmol) and CuBr (26 mg, 0.18 mmol) were mixed in 5 mL of dichloromethane at room temperature and stirred overnight. The volatiles were removed by evaporation under reduced pressure and the resulting colorless solid was washed with pentane (2 x 3 mL) and dried under vacuum (105 mg, 91%).

^{1}H NMR (CDCl$_3$, 400 MHz, 25 °C): δ 2.15 (s, 12 H, CH$_3$ Xyl), 6.23 (d, 2H, H$_f$), 6.98 (td, 2H, $^6J_{HP}$ = 0.9 Hz, H$_d$), 7.07 (d, 4H, H$_g$), 7.20 (d, 2H, H$_h$), 7.29 (d, 2H, H$_b$), 7.59 (t, 2H, H$_e$), 7.69 (t, 1H, H$_a$), 8.24 (dd, 2H, $^4J_{HP}$ = 2.3 Hz, H$_c$) ppm. $^3J_{HH}$ values are *ca.* 7-8 Hz.

^{31}P{^{1}H} NMR (CDCl$_3$, 162 MHz, 25 °C): δ 120.5 (s) ppm.

^{13}C{^{1}H} NMR (CDCl$_3$, 125 MHz, 25 °C): δ 21.0 (s, CH$_3$ Xyl), 112.2 (s, CH$_f$), 119.4 (s, CH$_d$), 127.4 (s, CH$_g$), 127.6 (s, CH$_h$), 127.6 (s, CH$_b$), 131.1 (s, CH$_a$), 132.3 (d, $^1J_{CP}$ = 27 Hz, C-P), 136.1 (d, $^4J_{CP}$ = 1 Hz, *C*-CH$_3$), 140.1 (s, CH$_e$), 140.3 (d, $^3J_{CP}$ = 5 Hz, C$_{ipso}$ Xyl), 145.6 (d, $^2J_{CP}$ = 21 Hz, *C*-CH$_b$), 146.9 (d, $^4J_{CP}$ = 2 Hz, CH$_c$), 158.1 (d, $^2J_{CP}$ = 9 Hz, C-O) ppm.

Elemental analysis calculated (found) for $C_{32}H_{29}BrCuN_2O_2P$: C, 59.3 (59.3), H, 4.5 (4.7), N 4.3 (4.5).

CuBr(κ^3-*N,P,N*-NOPON^{Xyl2}-Me$_2$), 2b

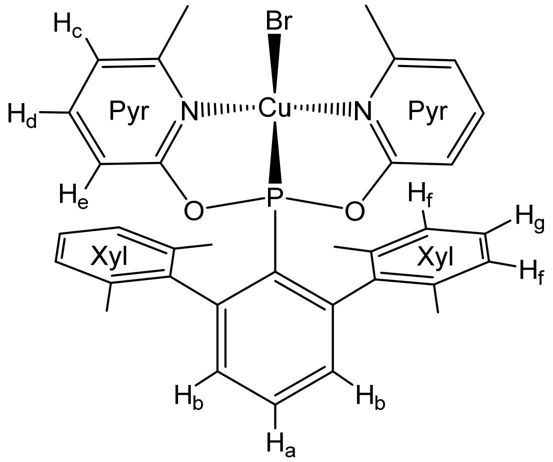

The compound was synthesized as described for CuBr(NOPON^{Xyl2}) using NOPON^{Xyl2}-Me$_2$ instead of NOPON^{Xyl2} (106 mg, 93%).

^{1}H NMR (CDCl$_3$, 400 MHz, 25 °C): δ 2.15 (s, 12H, CH$_3$ Xyl), 2.18 (s, 6H, CH$_3$ Pyr), 6.01 (d, 2H, H$_e$), 6.8 (dd, 2H, $^6J_{HP}$ =0.9 Hz, H$_c$), 6.99 (d, 4H, H$_f$), 7.06 (d, 2H, H$_g$), 7.19 (dd, 2H, $^4J_{HP}$ = 2.1 Hz, H$_b$), 7.43 (td, 2H, $^5J_{HP}$ = 2.4 Hz, H$_d$), 7.68 (t, 1H, H$_a$). $^3J_{HH}$ values are *ca.* 7-8 Hz.

^{31}P{^{1}H} NMR (CDCl$_3$, 162 MHz, 25 °C): δ 116.1 (s) ppm.

^{13}C{^{1}H} NMR (CDCl$_3$, 125 MHz, 25 °C): δ 21.4 (s, CH$_3$ Xyl), 25.6 (s, CH$_3$ Pyr), 109.3 (dd, $^3J_{CP}$ = 4Hz*,* CH$_e$), 118.8 (s, CH$_c$), 126.8 (s, CH$_f$), 127.1 (s, CH$_g$), 127.5 (dd, $^3J_{CP}$ = 2 Hz, CH$_b$), 130.1 (s, CH$_a$), 132.2 (dd, $^1J_{CP}$ = 27 Hz, C-P), 139.9 (dd, $^4J_{CP}$ = 2 Hz, *C*-CH$_3$ Xyl), 140.6 (d, $^4J_{CP}$ = 1 Hz, CH$_d$), 145.6 (d, $^3J_{CP}$ = 5 Hz, C$_{ipso}$ Xyl), 145.8 (d, $^2J_{CP}$ = 22 Hz, *C*-CH$_b$), 158.4 (d, $^4J_{CP}$ = 1 Hz, *C*-CH$_3$ Pyr), 161.1 (d, $^2J_{CP}$ = 9 Hz, C-O)ppm.

Elemental analysis calculated (found) for $C_{34}H_{33}BrCuN_2O_2P$: C, 60.4 (60.5), H, 4.9 (5.0), N 4.1 (4.1).

CuBr(κ^3-*N,P,N*-$N_2PN_2^{Xyl2}$), 2c

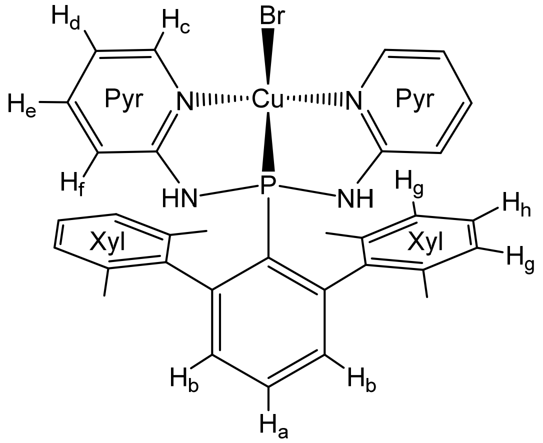

The compound was synthesized as described for CuBr(NOPON^{Xyl2}) using $N_2P_2N^{Xyl2}$ instead of NOPON^{Xyl2} (133 mg, 95%).

^{1}H NMR ($CDCl_3$, 400 MHz, 25 °C): δ 2.15 (s, 12H, CH_3 Xyl), 5.23 (s, 2H, NH), 5.99 (d, 2H, H_f), 6.71 (td, 2H, $^6J_{HP}$ = 0.9 Hz, H_d), 7.09 (d, 4H, H_g), 7.17 (t, 2H, H_h), 7.39 (m, 4H, H_b y H_e), 7.64 (t, 1H, H_a), 8.31 (dd, 2H, $^5J_{HP}$ = 2.3 Hz, H_c) ppm. $^3J_{HH}$ values are *ca.* 7-8 Hz.

$^{31}P\{^1H\}$ NMR ($CDCl_3$, 162 MHz, 25 °C): δ 21.8 (s) ppm.

$^{13}C\{^1H\}$ NMR ($CDCl_3$, 125 MHz, 25 °C): δ 21.28 (s, CH_3), 110.6 (d, $^3J_{CP}$ = 2 Hz, CH_f), 115.8 (s, CH_d), 127.9 (s, CH_g), 128.3 (s, CH_h), 130.3 (d, $^3J_{CP}$ = 2 Hz , CH_b), 131.2 (s, C-H_a), 135.9 (d, $^1J_{CP}$ = 27 Hz, C-P), 137.8 (d, $^4J_{CP}$ = 1 Hz, *C*-CH_3), 139.7 (s, CH_e), 144.8 (d, $^3J_{CP}$ = 5 Hz, C_{ipso} Xyl), 147.9 (d, $^2J_{CP}$ = 21 Hz, *C*-CH_b), 148.5 (d, $^4J_{CP}$ = 1 Hz, CH_c), 153.5 (d, $^2J_{CP}$ = 9 Hz, C-NH) ppm.

Elemental analysis calculated (found) for $C_{34}H_{35}BrCuN_4P$: C, 59.5 (59.6), H 4.8 (5.0), N 8.7 (8.6).

[Cu(NCMe)(κ³-*N,P,N*-NOPON^{Xyl2})]PF_6, 3a

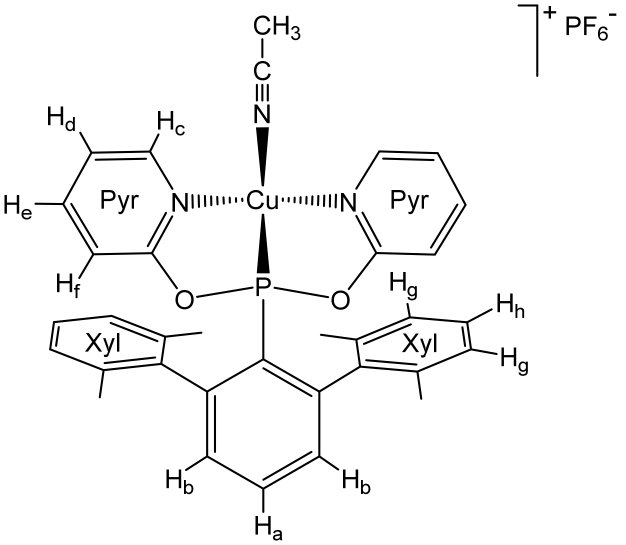

Equimolar amounts of NOPON^{Xyl2} (90 mg, 0.20 mmol) and [Cu(NCMe)$_4$]PF_6 (72 mg, 0.20 mmol) were dissolved in 5 mL of dichloromethane at room temperature and stirred overnight. The volatiles were removed by evaporation under reduced pressure and the resulting colorless solid was washed with pentane (2 x 3 mL) and dried under vacuum (125 mg, 89%).

^{1}H NMR ($CDCl_3$, 400 MHz, 25 °C): δ 2.1 (s, 12H, CH_3 Xyl), 2.45 (s, 3H, CH_3CN), 5.31 (s, 2H, NH), 6.35 (d, 2H, H_f), 6.98 (td, 2H, $^{6}J_{HP}$ = 0.8 Hz, H_d), 7.14 (d, 4H, H_g), 7.25 (t, 2H, H_h), 7.68 (m, 4H, H_b and H_e), 7.77 (t, 1H, H_a), 8.20 (dd, 2H, $^{5}J_{HP}$ = 2.1 Hz, H_c) ppm. $^{3}J_{HH}$ values are *ca.* 7-8 Hz.

^{31}P{^{1}H} NMR ($CDCl_3$, 162 MHz, 25 °C): δ 119.3 (s), -144.3 (sept, $^{1}J_{PF}$ = 707 Hz) ppm.

^{13}C{^{1}H} NMR ($CDCl_3$, 125 MHz, 25 °C): δ 2.5 (s, *C*H_3CN), 21.0 (s, CH_3 Xyl), 112.5 (s, CH_f), 120.5 (s, CH_d), 127.5 (s, CH_g), 127.8 (s, CH_h), 129.5 (d, $^{3}J_{CP}$ = 2 Hz , CH_b), 133.2 (s, CH_a), 136.1 (d, $^{1}J_{CP}$ = 28 Hz, C-P), 139.9 (d, $^{4}J_{CP}$ = 2 Hz, *C*-CH_3), 141.1 (s, CH_e), 145.9 (d, $^{3}J_{CP}$ = 5 Hz, C_{ipso} Xyl), 146.1 (d, $^{2}J_{CP}$ = 21 Hz, *C*-CH_b), 147.1 (d, $^{3}J_{CP}$ = 2 Hz, CH_c), 158.1 (d, $^{2}J_{CP}$ = 9 Hz, C-O) ppm.

Elemental analysis calculated (found) for $C_{34}H_{32}CuF_6N_3O_2P_2$: C, 54.2 (54.2); H, 4.3 (4.6); N, 5.6 (5.5).

[Cu(NCMe)(κ^3-*N,P,N*-NOPON^{Xyl}-Me$_2$)]PF$_6$, 3b

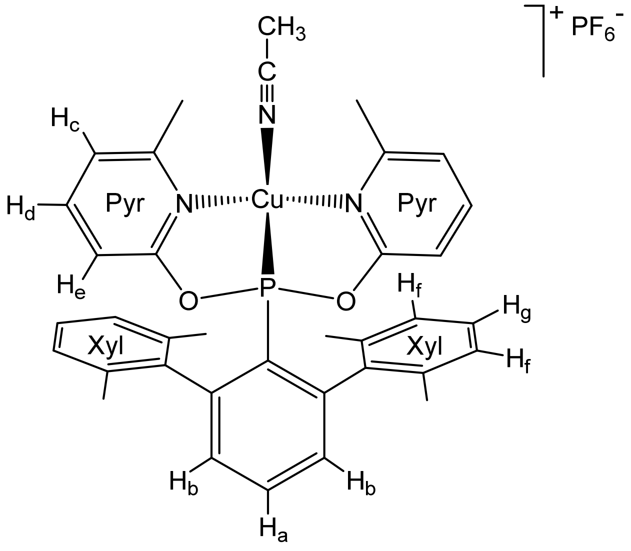

The compound was synthesized as described for [Cu(NCMe)(NOPON^{Xyl2})]PF$_6$ using NOPON^{Xyl2}-Me$_2$ instead of NOPON^{Xyl2} (125 mg, 91%).

^{1}H NMR (CDCl$_3$, 400 MHz, 25 °C): δ 2.10 (s, 12H, CH$_3$ Xyl), 2.48 (s, 3H, CH$_3$CN), 2.54 (s, 6H, CH$_3$ Pyr), 6.14 (d, 2H, H$_e$), 6.78 (dd, 2H, $^5J_{HP}$ =0.9 Hz, H$_c$), 7.00 (d, 4H, H$_f$), 7.12 (d, 2H, H$_g$), 7.27 (dd, 2H, $^4J_{HP}$ = 2.1 Hz, H$_b$), 7.49 (td, 2H, $^4J_{HP}$ =2.4 Hz, H$_d$), 7.77 (t, 1H, H$_a$). $^3J_{HH}$ values are *ca.* 7-8 Hz.

^{31}P{^{1}H} NMR (CDCl$_3$, 162 MHz, 25 °C): δ 118.2 (s), -145.7 (sept, $^1J_{PF}$ = 707 Hz) ppm.

^{13}C{^{1}H} NMR (CDCl$_3$, 125 MHz, 25 °C): δ 2.74 (s, *C*H$_3$CN); 21.3 (s, CH$_3$ Xyl), 25.5 (s, CH$_3$ Pyr), 109.9 (d, $^3J_{CP}$ = 4 Hz, CH$_e$), 119.9 (s, CH$_d$), 127.4 (s, CH$_f$), 127.8 (s, CH$_g$), 130.2 (d, $^3J_{CP}$ = 2Hz , CH$_b$), 133.3 (s, CH$_a$), 136.1 (d, $^1J_{CP}$ = 27 Hz, C-P), 140.0 (d, $^4J_{CP}$ = 2Hz, *C*-CH$_3$ Xyl), 141.3 (d, $^4J_{CP}$ = 1Hz, CH$_d$), 145.9 (d, $^3J_{CP}$ = 6 Hz, C$_{ipso}$ Xyl), 148.5 (d, $^2J_{CP}$ = 21 Hz, *C*-CH$_b$), 156.1 (d, $^4J_{CP}$ = 1 Hz, *C*-CH$_3$ Pyr), 158.3 (d, $^2J_{CP}$ = 8 Hz, C-O) ppm.

Elemental analysis calculated (found) for $C_{34}H_{36}CuF_6N_3O_2P_2$: C, 55.3 (55.4); H, 4.6 (4.8); N, 5.4 (5.1).

[Cu(NCMe)(κ^3-*N,P,N*-$N_2PN_2^{Xyl2}$)]PF_6, 3c

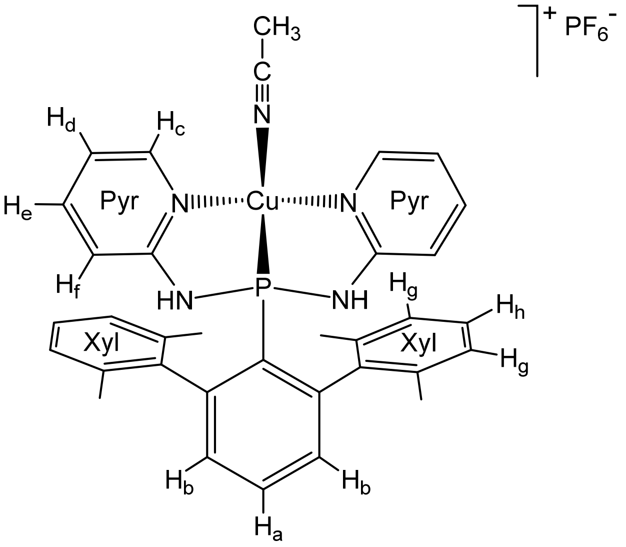

The compound was synthesized as described for [Cu(NCMe)($NOPON^{Xyl2}$)]PF_6 using $N_2P_2N^{Xyl2}$ instead of $NOPON^{Xyl2}$ (129 mg, 92%).

^{1}H NMR (CD_2Cl_2, 400 MHz, 25 °C): δ 2.12 (s, 12H, CH_3 Xyl), 2.38 (s, 3H, CH_3CN), 5.39 (s, 2H, NH), 6.21 (d, 2H, H_f), 6.80 (dd, 2H, $^6J_{HP}$ =0.8 Hz, H_d), 7.11 (d, 4H, H_g), 7.26 (d, 2H, H_h), 7.52 (dd, 2H, $^4J_{HP}$ = 2.4 Hz, H_b), 7.74 (td, 2H, $^5J_{HP}$ =2.6 Hz, H_e), 7.96 (t, 1H, H_a) ppm. $^3J_{HH}$ values are *ca.* 7-8 Hz.

$^{31}P\{^1H\}$ NMR (CD_2Cl_2, 162 MHz, 25 °C): δ 20.4 (s), -144.4 (sept, $^1J_{PF}$ = 707 Hz) ppm.

$^{13}C\{^1H\}$ NMR (CD_2Cl_2, 125 MHz, 25 °C): δ 2.4 (s, *C*H_3CN), 20.9 (s, CH_3 Xyl), 111.7 (d, $^3J_{CP}$ = 2 Hz*,* CH_f), 116.2 (s, CH_d), 127.8 (s, CH_g), 128.2 (s, CH_h), 130.5 (d, $^3J_{CP}$= 2 Hz, CH_b), 132.1 (s, CH_a), 136.1 (d, $^1J_{CP}$ = 34 Hz, C-P), 138.9 (d, $^4J_{CP}$ = 1 Hz, *C*- CH_3), 139.2 (s, CH_e), 139.7 (d, $^3J_{CP}$ = 5 Hz, C_{ipso} Xyl), 142.9 (d, $^2J_{CP}$ = 21 Hz, *C*-CH_b), 146.7 (d, $^4J_{CP}$ = 1 Hz, CH_c), 154.1 (d, $^2J_{CP}$ = 9 Hz, C-NH) ppm.

Elemental analysis calculated (found) for $C_{34}H_{34}CuF_6N_5P_2$: C, 54.3 (54.2); H, 4.6 (4.5); N, 9.3 (9.0).

2. General Catalytic Procedure for the S-Arylation of Thiols with Aryl Iodides

The catalyst **2a** or **2b** (0.02 mmol) was dissolved in dioxane (1 mL) in an ampule. The aryl iodide (1.2 mmol), the thiol (1.0 mmol), and the base, K_3PO_4 (2.0 mmol), were added under a nitrogen atmosphere. The reaction was stirred at 110 °C for 24 h in an oil bath. The reaction mixture was allowed to cool to room temperature, diluted with ethyl acetate (10 mL) and filtered through Celite plug. The conversion was determined by GC analysis. Pure products were obtained after purification by flash chromatography on silica gel with petroleum ether (otherwise indicated).

3. Characterization data for thioethers.

Diphenylsulfane[2] (Scheme 5, **a**)

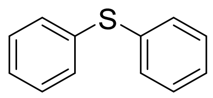

Colorless oil. Yield with catalyst **2a**: 169 mg, 91%. Yield with catalyst **2b**: 167 mg, 90%. ^{1}H NMR (300 MHz, $CDCl_3$): δ 7.51-7.32 (m, 10 H) ppm.

(3,5-Dimethylphenyl)(phenyl)sulfane[2] (Scheme 5, **b**)

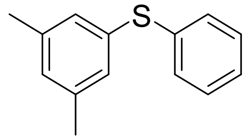

Colorless oil. Yield with catalyst **2a**: 206 mg, 96%. Yield with catalyst **2b**: 201 mg, 94%. ^{1}H NMR (300 MHz, $CDCl_3$): δ 7.43-7.39 (m, 2H), 7.28-7.09 (m, 4H), 6.92 (bs, 1H), 6,80 (bs, 1H), 2.18 (s, 6H) ppm.

---

[2] C. G. Bates, R. K. Gujadhur, D. Venkataraman, *Org. Lett.* **2002**, *4*, 2803–2806.

Phenyl(4-methylphenyl)sulfane[2] (Scheme 5, **c**)

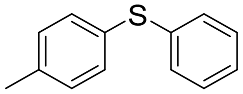

Colorless oil. Yield with catalyst **2a**: 190 mg, 95%. Yield with catalyst **2b**: 186 mg, 93%. ^{1}H NMR (300 MHz, $CDCl_3$): δ 7.42-7.39 (m, 1H), 7.23-7.03 (m, 8H), 2.25 (s, 3H) ppm.

(4-Methoxyphenyl)(phenyl)sulfane[2] (Scheme 5, **d**)

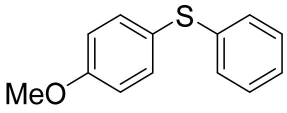

Colorless oil. Yield with catalyst **2a**: 164 mg, 76%. Yield with catalyst **2b**: 162 mg, 75%. ^{1}H NMR (300 MHz, CDCl3): δ 7.34-7.31 (m, 2H,), 7.17-7.01 (m, 5H), 6.83-6.79 (m, 2H), 3.72 (s, 3H) ppm.

Phenyl(2-methylphenyl)sulfane[2] (Scheme 5, **e**)

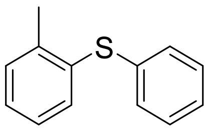

Colorless oil. Yield with catalyst **2a**: 176 mg, 88%. Yield with catalyst **2b**: 174 mg, 87%. ^{1}H NMR (300 MHz, $CDCl_3$): δ 7.23-7.01 (m, 9H), 2.29 (s, 3H) ppm.

o-tolyl(p-tolyl)sulfane[3] (Scheme 5, **f**)

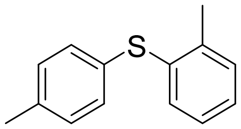

Colorless oil. Yield with catalyst **2a**: 176 mg, 89%. Yield with catalyst **2b**: 172 mg, 89%. ^{1}H NMR (300 MHz, $CDCl_3$): δ 7.29-7.16 (m, 8H), 2.46 (s, 3H), 2.40 (s, 3H) ppm.

[3] N. Taniguchi, T. Onami, *J. Org. Chem*. **2004**, *69*, 915–920.

(4-Methoxyphenyl)(*p*-tolyl)sulfane[4] (Scheme 5, **g**)

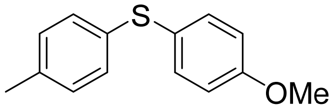

Colorless solid. Purification by column chromatography in silica gel with a mixture 1:50 ethyl acetate/petroleum ether. Yield with catalyst **2a**: 200 mg, 87%. Yield with catalyst **2b**: 198 mg, 86%. ^{1}H NMR (300 MHz, $CDCl_3$): δ 7.47 (d, *J* = 8.8 Hz, 2H), 7.25 (d, *J* = 8.0 Hz, 2H), 7.16 (d, *J* = 8.0 Hz, 2H), 6.96 (d, *J* = 8.8 Hz, 2H), 3.87 (s, 3H), 2.40 (s, 3H).

(4-Chlorophenyl)(phenyl)sulfane[5] (Scheme 5, **h**)

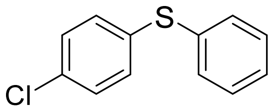

Colorless oil. Yield with catalyst **2a**: 196 mg, 89%. Yield with catalyst **2b**: 193 mg, 88%. ^{1}H NMR (300 MHz, $CDCl_3$): δ 7.27-7.18 (m, 9H) ppm.

(4-Bromophenyl)(phenyl)sulfane[6] (Scheme 5, **i**)

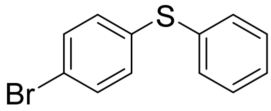

Colorless oil. Yield with catalyst **2a**: 242.9 mg, 92%. Yield with catalyst **2b**: 245.5 mg, 93%. ^{1}H NMR (300 MHz, $CDCl_3$): δ 7.48 (d, *J* = 8.3 Hz, 2H), 7.41-7.22 (m, 5H), 6.92 (d, *J* = 8.3 Hz, 2H) ppm.

---

[4] I. W. J. Still, F. D. Toste, *J. Org. Chem*. **1996**, *61*, 7677–7680

[5] W. Deng, Y. Zou, Y. F. Wang, L. Liu, Q. X. Guo, *Synlett* **2004**, 1254–1258.

[6] L. Rout, T. K. Sen, T. Punniyamurthy, *Angew. Chemie - Int. Ed*. **2007**, *46*, 5583– 5586.

4-(phenylthiol)pyridine[7] (Scheme 5, **j**)

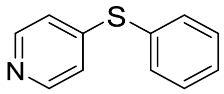

Colorless oil. Yield with catalyst **2a**: 175.9 mg, 94%. Yield with catalyst **2b**: 172.3 mg, 92%. ^{1}H NMR (300 MHz, $CDCl_3$): δ 8.26 (d, *J* = 6.0 Hz, 2H), 7.49-7.43 (m, 2H), 7.39-7.34 (m, 3H), 6.86 (d, *J* = 6.0 Hz, 2H) ppm.

2-(4-Methylphenylthio)naphthalene[8] (Scheme 5, **k**)

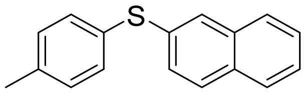

White solid. Yield with catalyst **2a**: 190.1 mg, 76%. Yield with catalyst **2b**: 195.2 mg, 78%. ^{1}H-NMR (300 MHz, $CDCl_3$): δ 7.72-7.57 (m, 4H), 7.36-7.29 (m, 2H), 7.26-7.22 (m, 3H), 7.06 (d, *J* = 7.7 Hz, 2H), 2.28 (s, 3H) ppm.

(4-fluorophenyl)(4-tolyl)sulfane[9] (Scheme 5, **l**)

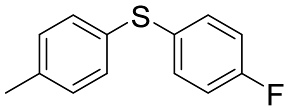

Colorless oil. Yield with catalyst **2a**: 200.6 mg, 92%. Yield with catalyst **2b**: 196.3 mg, 90%. ^{1}H NMR (300 MHz, $CDCl_3$): δ 7.23–7.17 (m, 2H), 7.15 (d, 2H, *J* = 7.4 Hz), 7.01 (d, 2H, *J* = 7.4 Hz), 6.93–6.86 (m, 2H), 2.23 (s, 3H, CH_3) ppm.

[7] F. Trécourt, G. Breton, V. Bonnet, F. Mongin, F. Marsais, G. Quéguiner, *Tetrahedron* **2001**, *56*, 1349–1360.
[8] T. Nakazawa, N. Hirose, K. Itabashi, *Synthesis* **1989**, 955.
[9] P. Buranaprasertsuk, J. W. W. Chang, W. Chavasiri, P. W. H. Chan, *Tetrahedron Lett.* **2008**, *49*, 2023.

4-(4-Methylphenylthio)aniline [10] (Scheme 5, **m**)

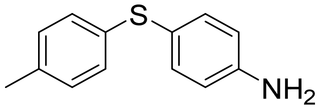

Colorless oil. Purification by column chromatography in silica gel with a mixture 1:20 ethyl acetate/petroleum ether. Yield with catalyst **2a**: 194 mg, 90%. Yield with catalyst **2b**: 191 mg, 89%. ^{1}H NMR (300 MHz, $CDCl_3$): δ 7.21-7.14 (m, 2H), 7.06-6.94 (m, 4H), 6.59-6.54 (m, 2H), 3.67 (bs, 2H), 2.21 (s, 3H) ppm.

4-(p-tolylthio)phenol [11] (Scheme 5, **n**)

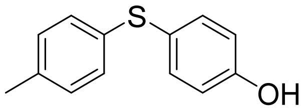

White solid. Purification by column chromatography in silica gel with a mixture 1:20 ethyl acetate/petroleum ether. Yield with catalyst **2a**: 190 mg, 88%. Yield with catalyst **2b**: 186 mg, 86%. ^{1}H NMR (300 MHz, $CDCl_3$): δ 7.30 (d, *J* = 8.4 Hz, 2H), 7.14 (d, *J* = 8.0 Hz, 2H), 7.06 (d, *J* =8.0 Hz, 2H), 6.78 (d, *J* =8.8 Hz, 2H), 4.96 (brs, 1H), 2.30 (s, 3H) ppm.

Cyclohexyl(2-methylphenyl)sulfane [12] (Scheme 5, **o**)

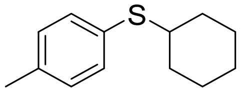

Colorless oil. Purification by column chromatography in silica gel with a mixture 1:50 ethyl acetate/petroleum ether. Yield with catalyst **2a**: 190 mg, 92%. Yield with catalyst **2b**: 188 mg, 91%. ^{1}H NMR (300 MHz, $CDCl_3$): δ 7.24 (d, *J* = 7.5 Hz, 2H), 7.02 (d, *J* = 7.5 Hz, 2H), 2.99- 2.89 (m, 1H), 2.25 (s, 3H), 1.99-1.82 (m, 3H), 1.75-1.65 (m, 2H), 1.56-1.50 (m, 2H), 1.30-1.17(m, 2H) ppm.

[10] W. Zhang, M. Huang, Z. Zou, Z. Wu, S. Ni, L. Kong, Y. Zheng, Y. Wang, Y. Pan, *Chem. Sci.* **2021**, *12*, 2509–2514.
[11] Y. C. Wong, T. T. Jayanth, C. H. Cheng, *Org. Lett*. **2006**, *8*, 5613-5616.
[12] H. L. Kao, C. K. Chen, Y. J. Wang, C. F. Lee, *European J. Org. Chem*. **2011**, 1776–1781.

4-(Cyclohexylthio)pyridine [13] (Scheme 5, **p**)

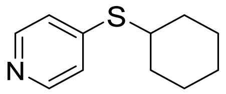

Colorless oil. Purification by column chromatography in silica gel with a mixture 1:20 ethyl acetate/petroleum ether. Yield with catalyst **2a**: 182 mg, 94%. Yield with catalyst **2b**: 180 mg, 93%. ^{1} H NMR (300 MHz, $CDCl_3$): δ 8.30 (d, *J* = 5.3 Hz, 2H), 7.04 (d, *J* = 5.3 Hz, 2H), 3.31- 3.23 (m, 1H), 2.03-1.96 (m, 2H), 1.78-1.71 (m, 2H), 1.62-1.55 (m, 1H), 1.46-1-15 (m, 4H) ppm.

tert-Butyl(p-tolyl)sulfane [14] (Scheme 5, **q**)

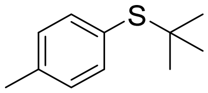

Colorless oil. Yield with catalyst **2a**: 151 mg, 84%. Yield with catalyst **2b**: 153 mg, 85%. ^{1}H NMR (300 MHz, $CDCl_3$): δ 7.39-7.31 (m, 1H), 7.18-7.01 (m, 3H), 2.00 (s, 3H), 1.20 (s, 9H) ppm.

4-(Butylthio)pyridine [15] (Scheme 5, **r**)

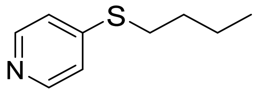

Colorless oil. Purification by column chromatography in silica gel with a mixture 1:20 ethyl acetate/petroleum ether. Yield with catalyst **2a**: 139 mg, 83%. Yield with catalyst **2b**: 135 mg, 81%. ^{1}H NMR (300 MHz, $CDCl_3$): δ 8.31-8.30 (m, 2H), 7.05-6.99 (m, 2H), 2.89 (t, *J* = 7.4 Hz, 2H), 1.65 (q, *J* = 7.4 Hz, 2H), 1.40 (s, *J* = 7.4 Hz, 2H), 0.88 (t, *J* = 7.4 Hz, 3H) ppm.

---

[13] B. Du, B. Jin, P. Sun, *Org. Lett.* **2014**, *16*, 3032–3035.
[14] T. C. Pijper, J. Robertus, W. R. Browne, B. L. Feringa, *Org. Biomol. Chem*. **2015**, *13*, 265–268.
[15] Y. Zhang, M. Q. Hu, H. M. Wen, Y. T. Si, C. B. Ma, C. N. Chen, Q. T. Liu, J. *Organomet. Chem*. **2009**, *694*, 2576–2580.

4. NMR spectra

^{1}H NMR spectrum of NOPON^{Xyl_2}

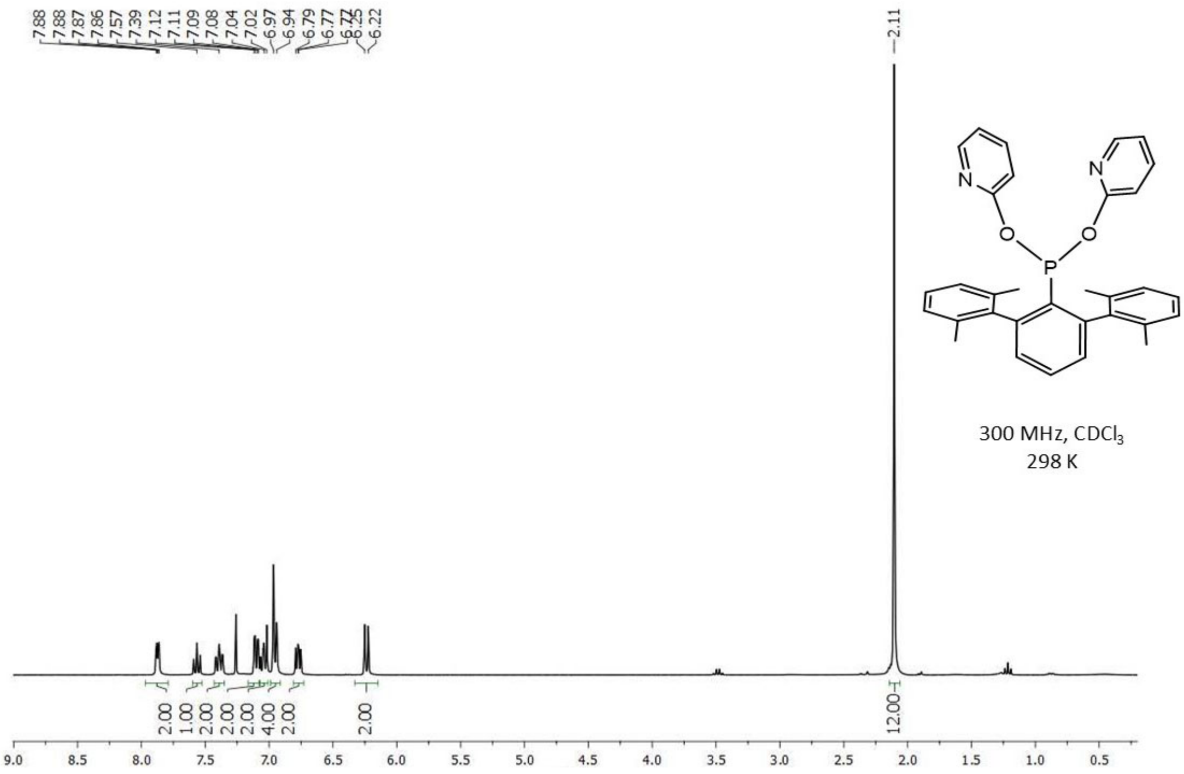

$^{13}C\{^{1}H\}$ NMR spectrum of NOPON^{Xyl_2}

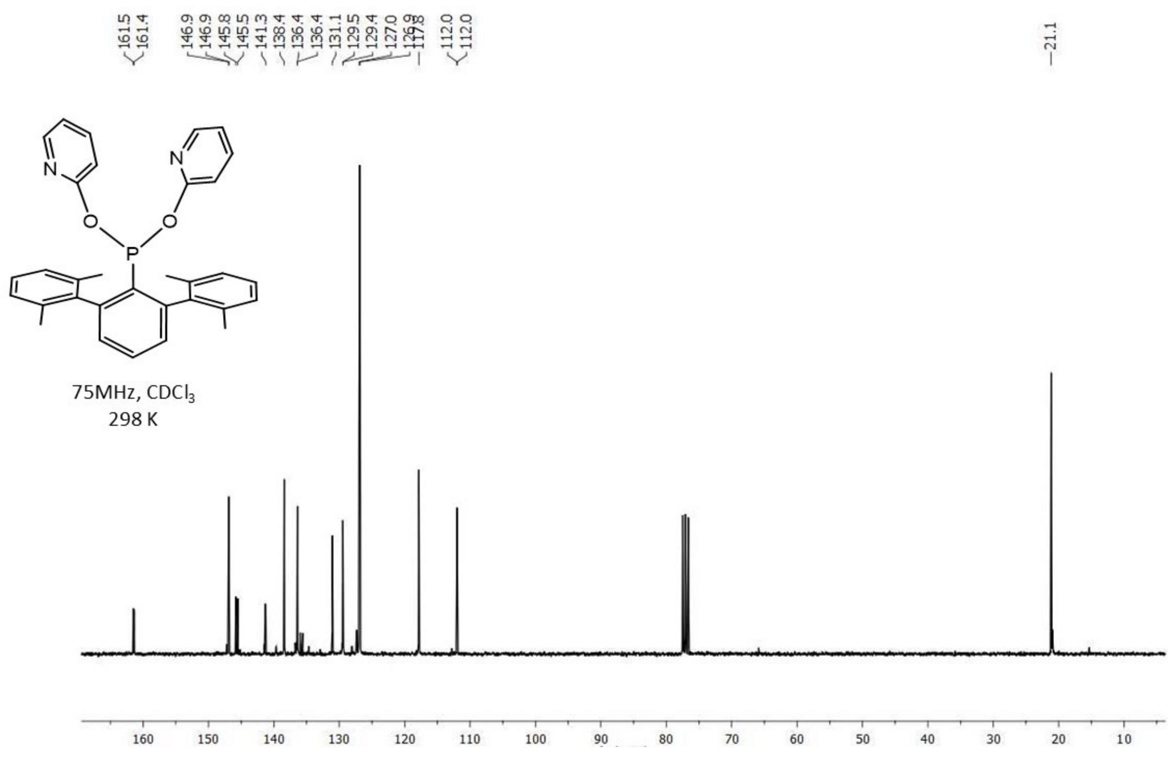

$^{31}P\{^1H\}$ NMR spectrum of NOPON^{Xyl_2}

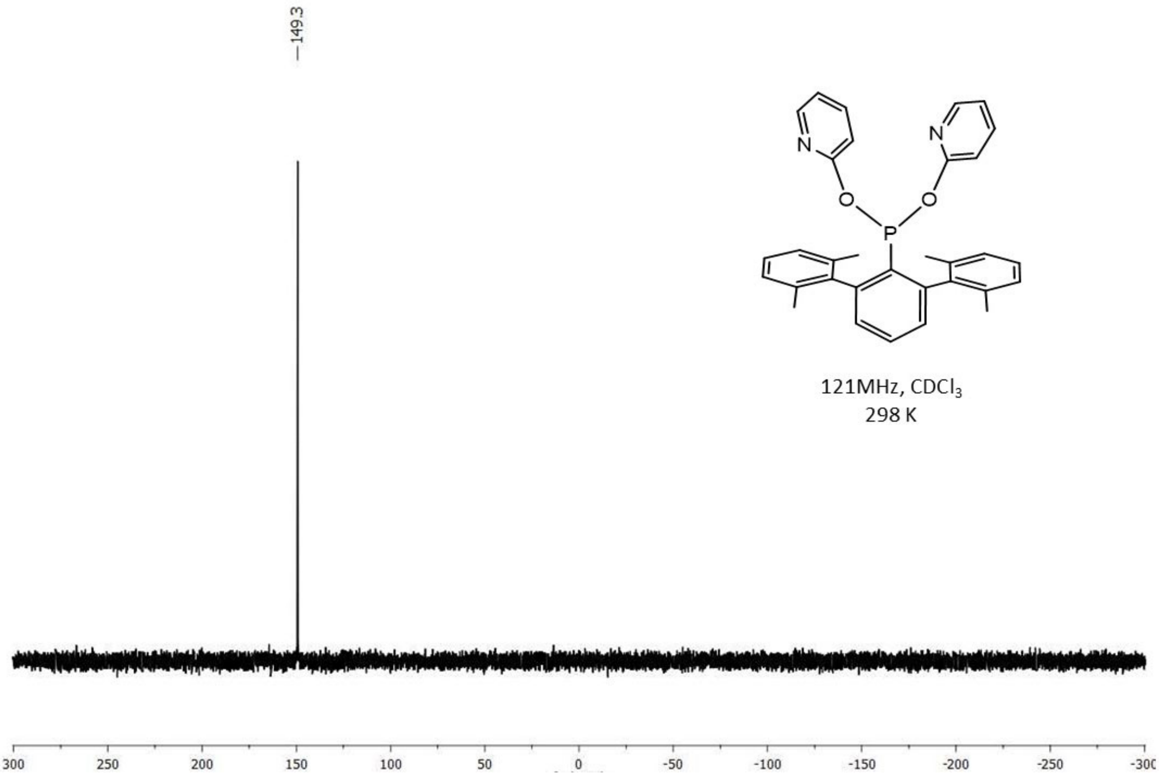

^{1}H NMR spectrum of NOPON$^{Xyl_2\text{-}Me_2}$

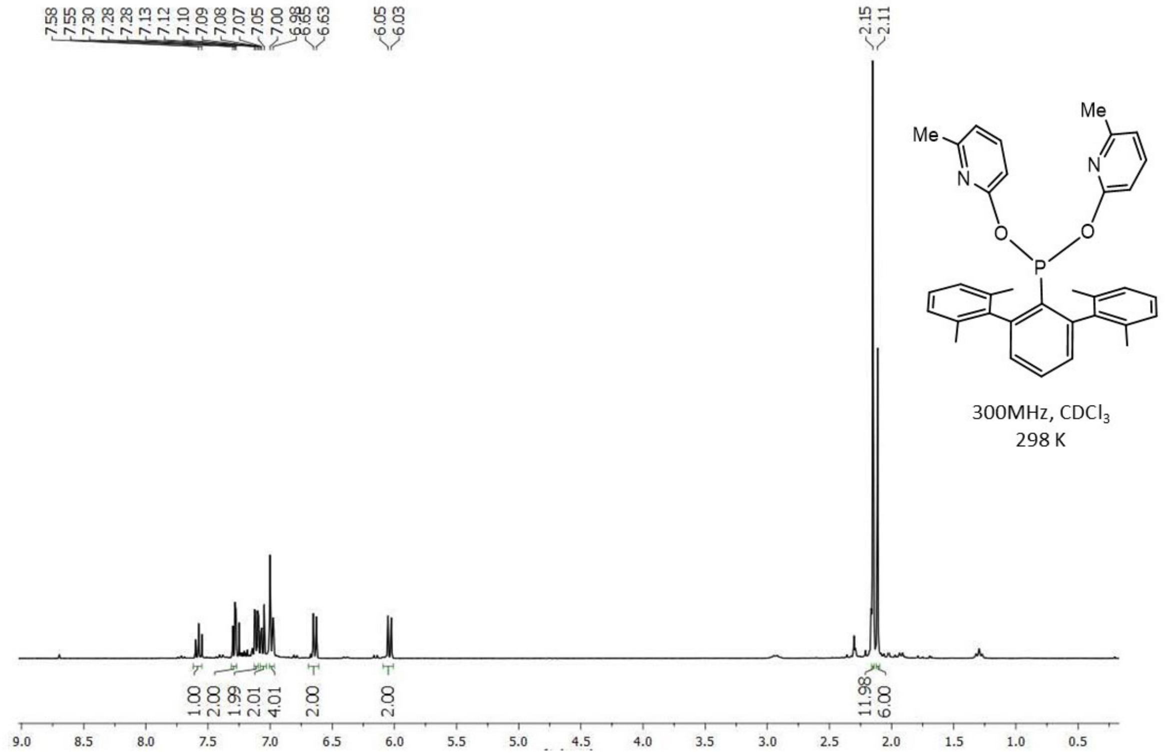

$^{13}C\{^{1}H\}$ NMR spectrum of NOPON$^{Xyl_2\text{-}Me_2}$

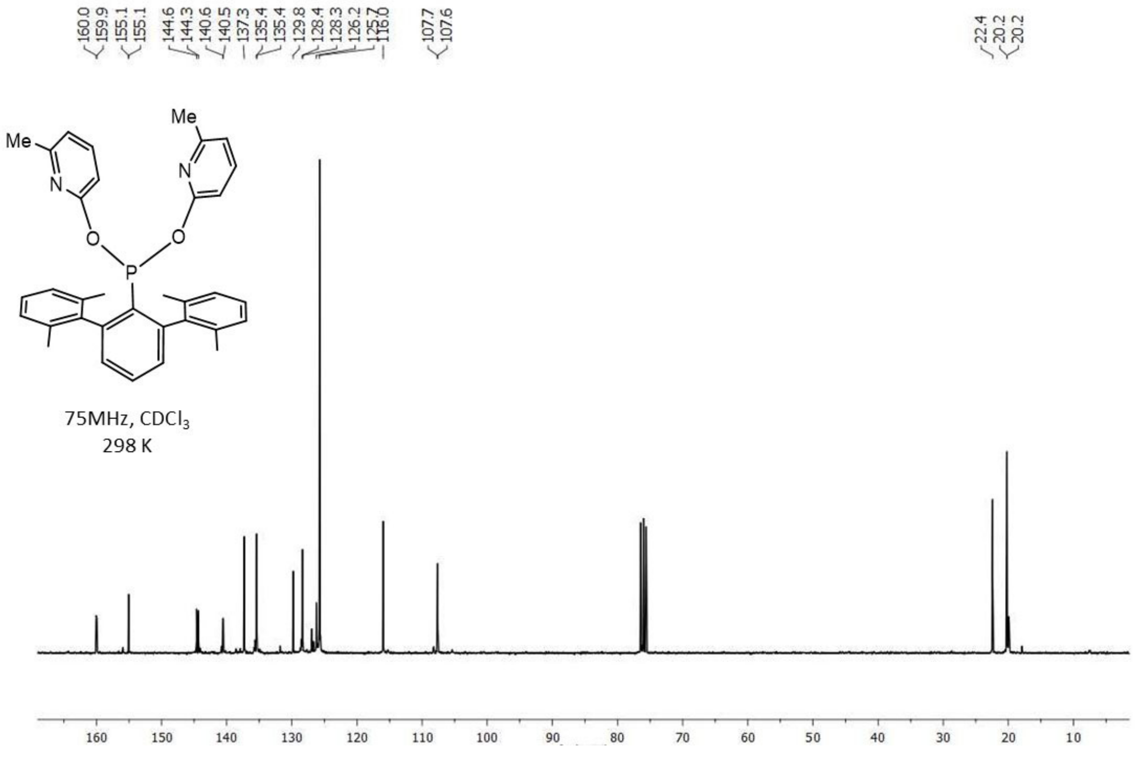

$^{31}P\{^{1}H\}$ NMR spectrum of NOPON$^{Xyl_2\text{-}Me_2}$

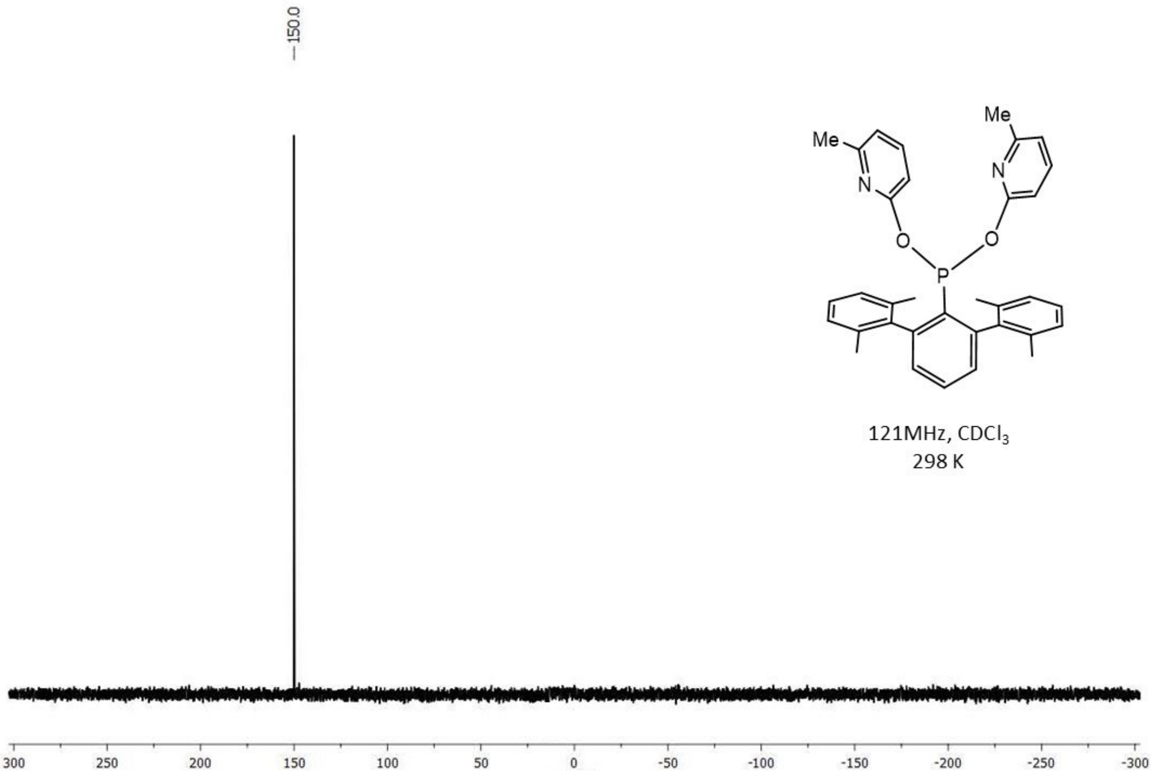

^{1}H NMR spectrum of $N_2PN_2^{Xyl_2}$

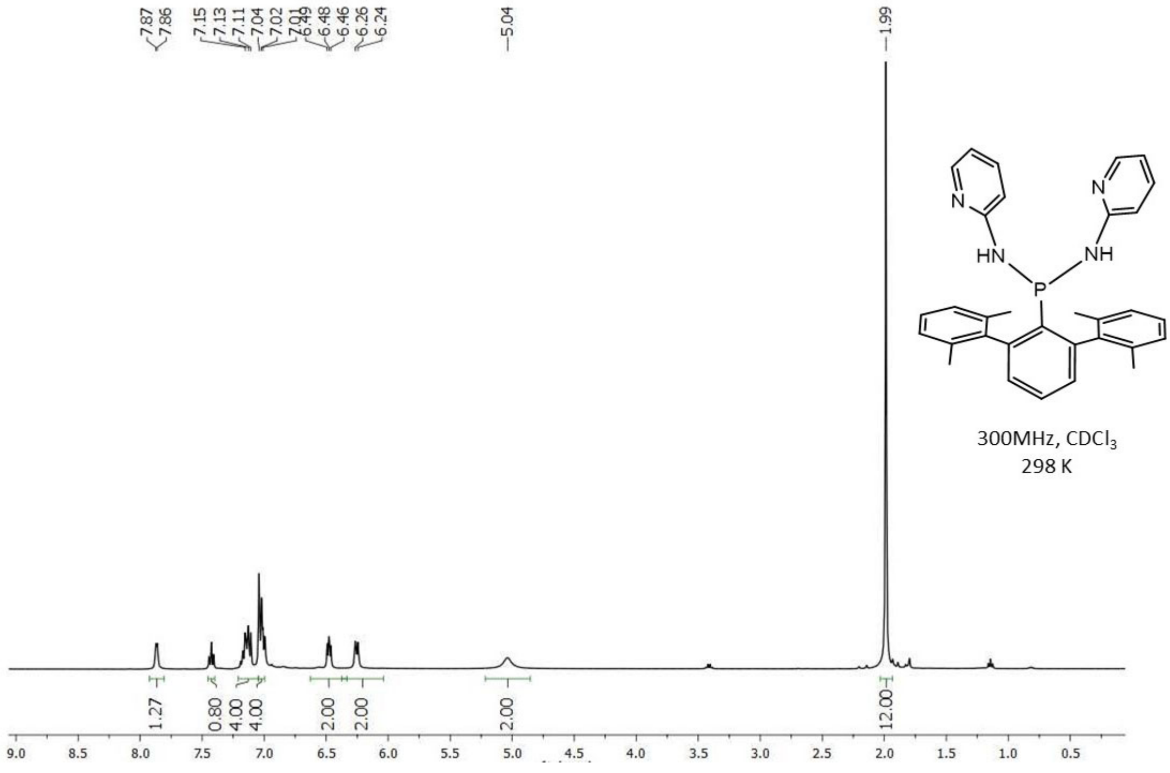

$^{13}C\{^{1}H\}$ NMR spectrum of $N_2PN_2^{Xyl_2}$

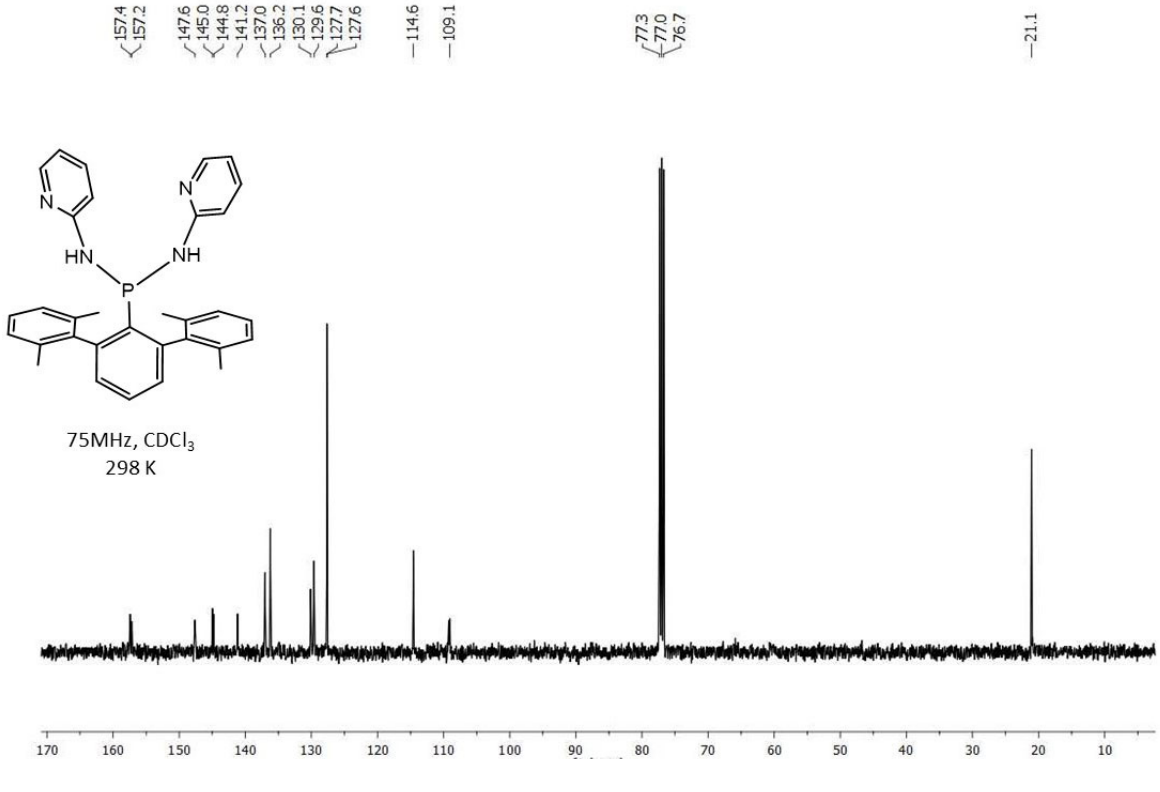

$^{31}P\{^{1}H\}$ NMR spectrum of $N_2PN_2^{Xyl_2}$

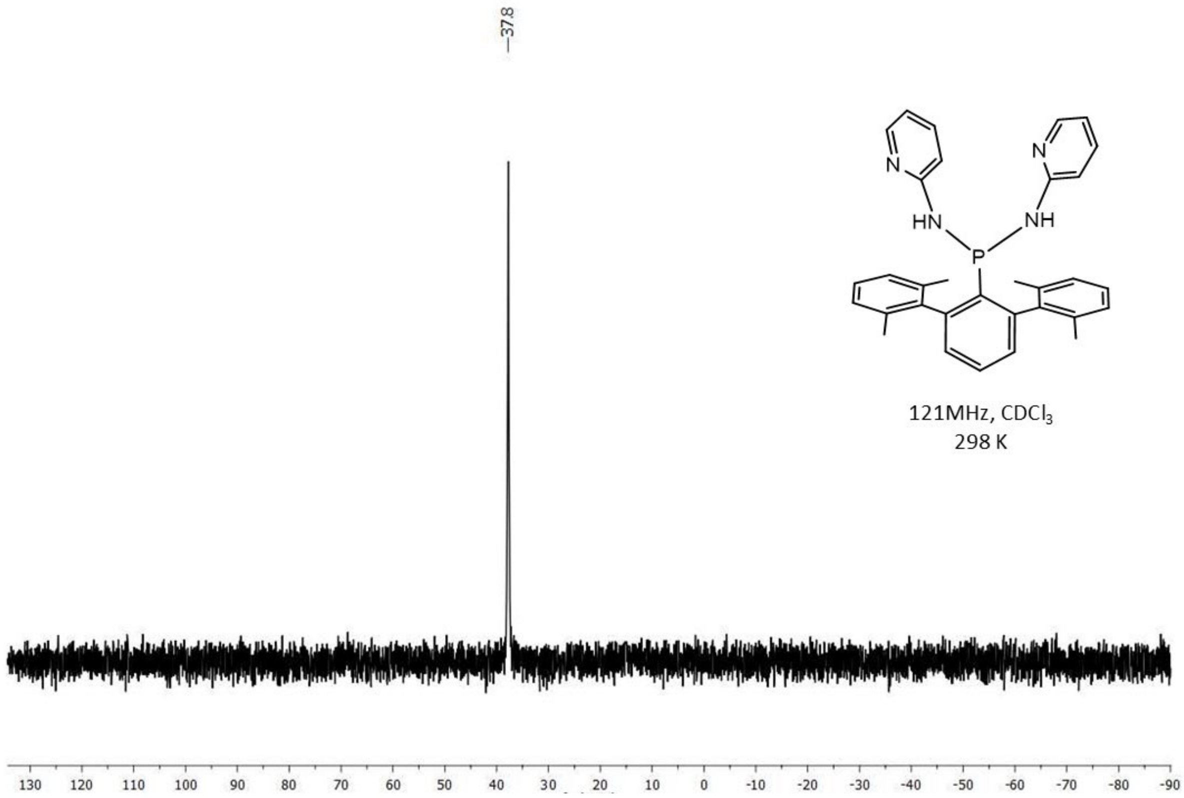

Hydrolysis of NOPON^{Xyl_2} and NOPON$^{Xyl_2\text{-}Me_2}$

^{1}H NMR spectrum of a 1:2 mixture of **1** and 2-hydroxypyridine.

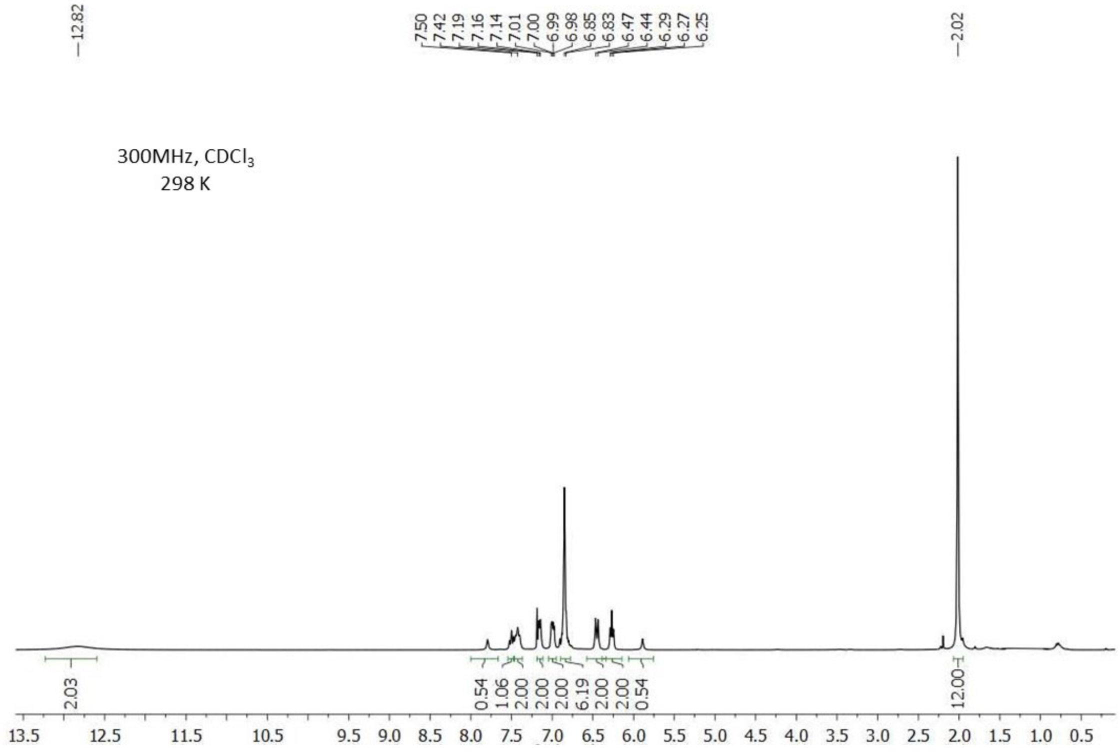

^{13}C{^{1}H} NMR spectrum of a 1:2 mixture of **1** and 2-hydroxypyridine.

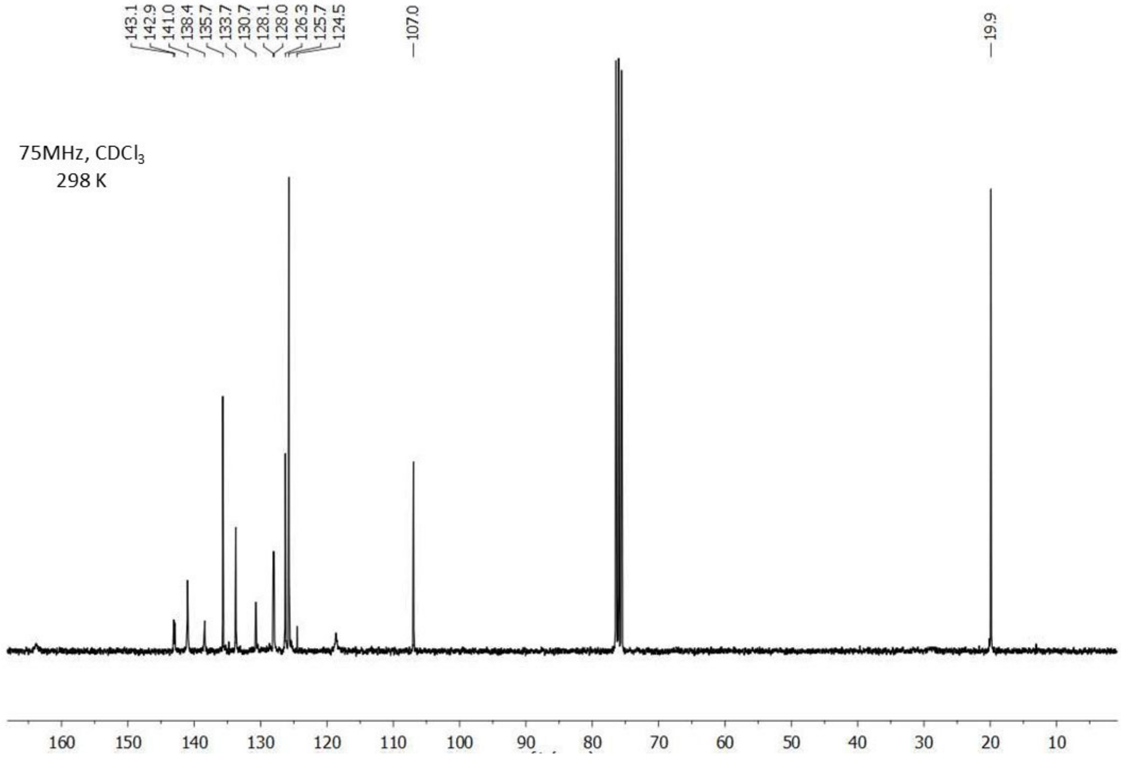

$^{31}P\{^1H\}$ NMR spectrum of a 1:2 mixture of **1** and 2-hydroxypyridine.

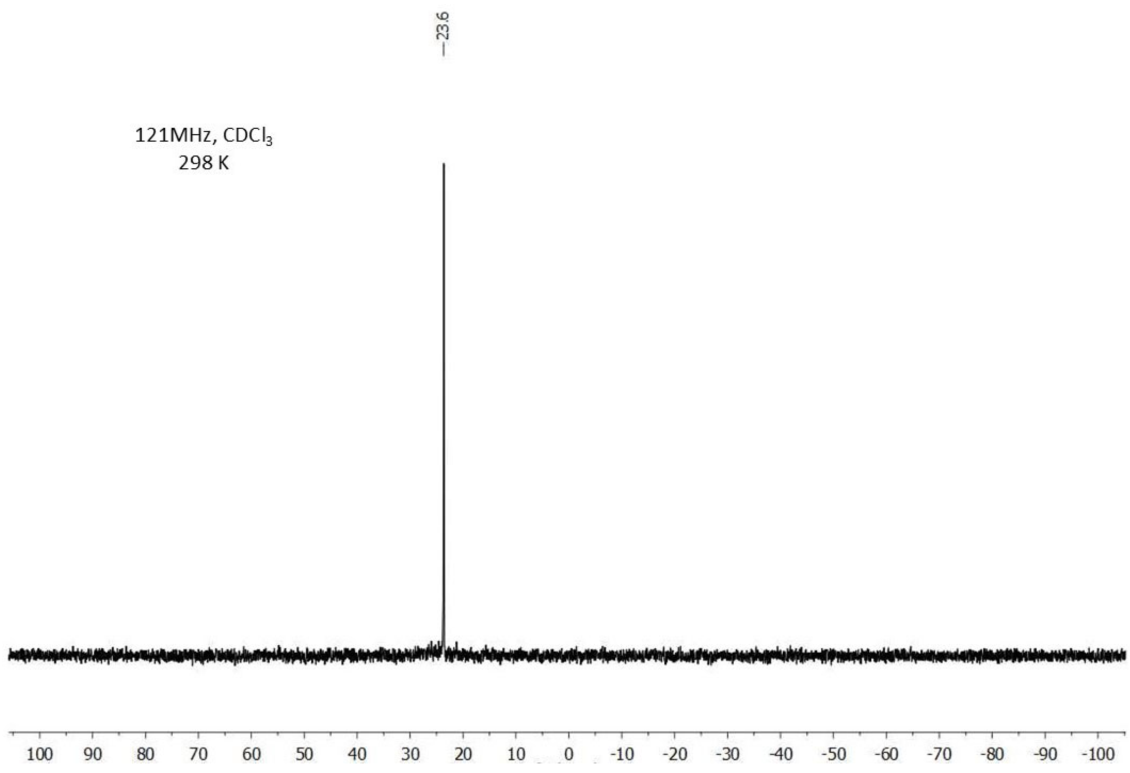

^{1}H NMR spectrum of a 1:2 mixture of **1** and 6-methyl-2-hydroxypyridine.

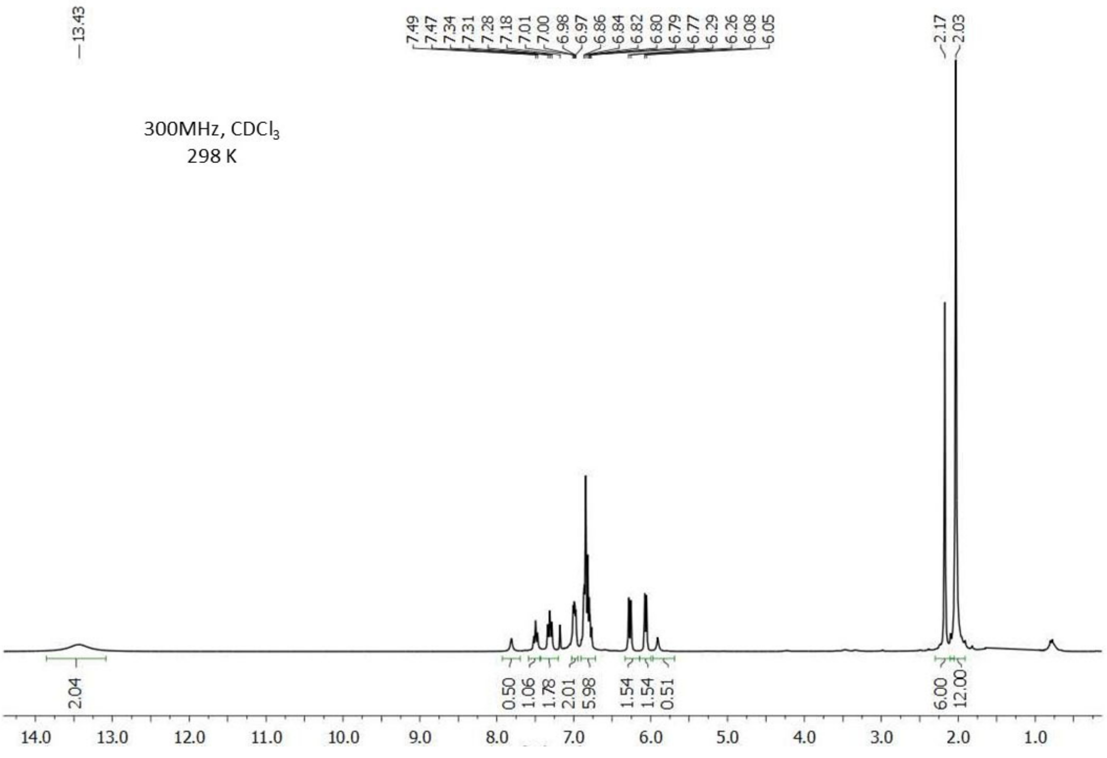

$^{13}C\{^{1}H\}$ NMR spectrum of a 1:2 mixture of **1** and 6-methyl-2-hydroxypyridine.

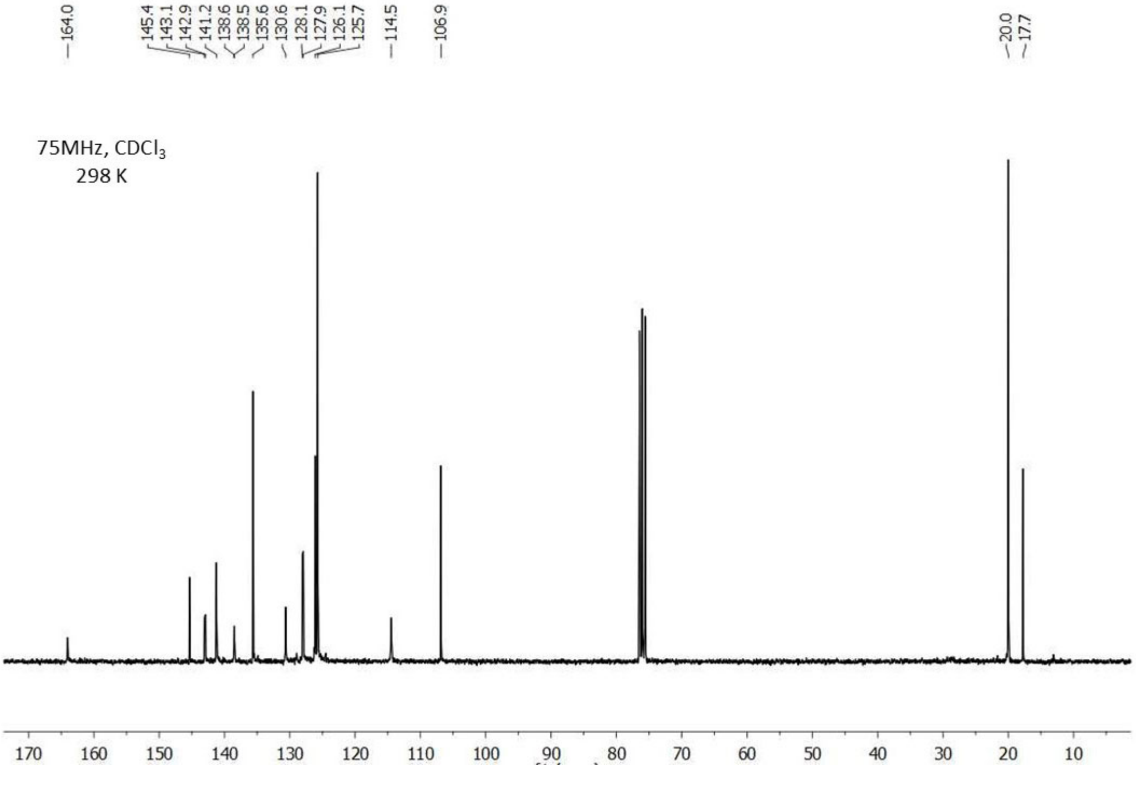

$^{31}P\{^1H\}$ NMR spectrum of a 1:2 mixture of **1** and 6-methyl-2-hydroxypyridine.

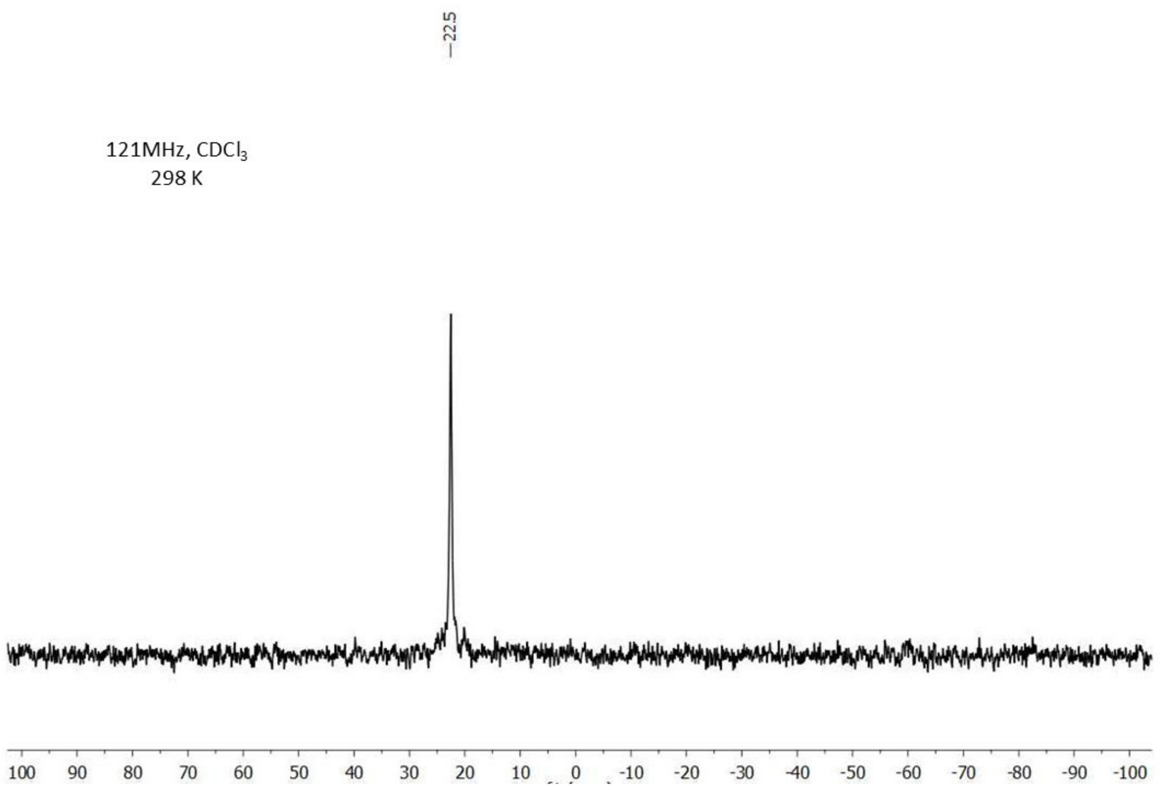

^{1}H NMR spectrum of 2a

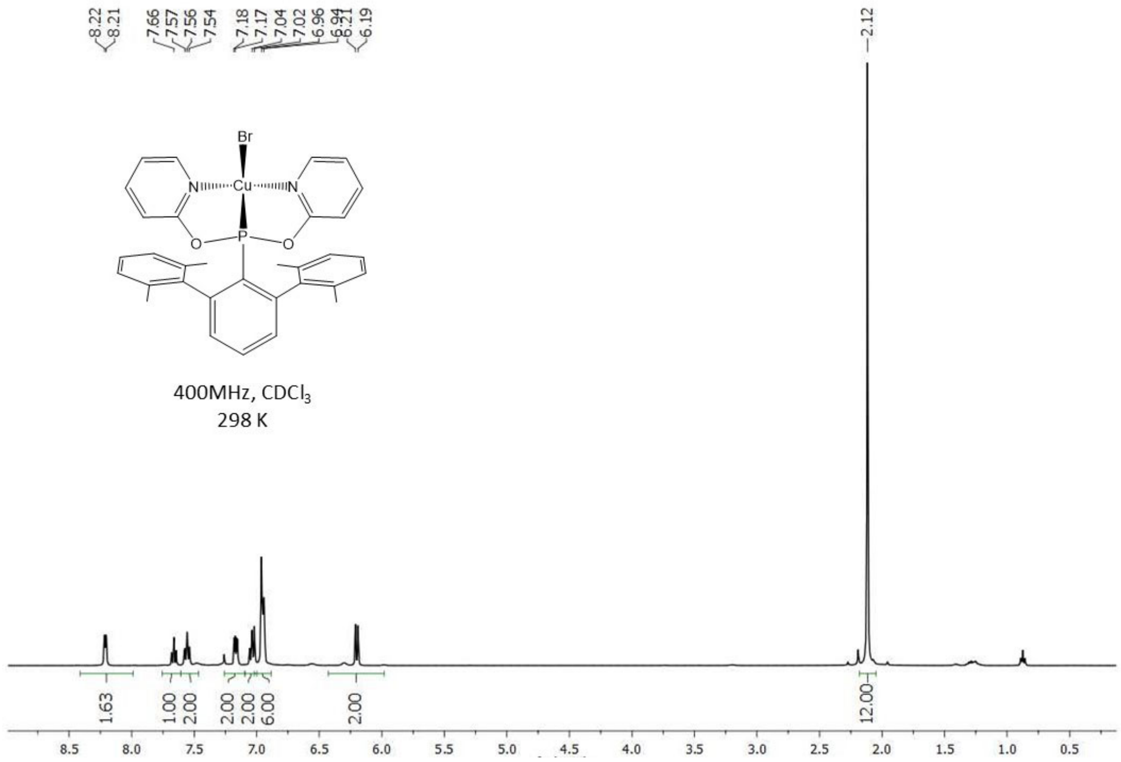

^{13}C{^{1}H} NMR spectrum of 2a

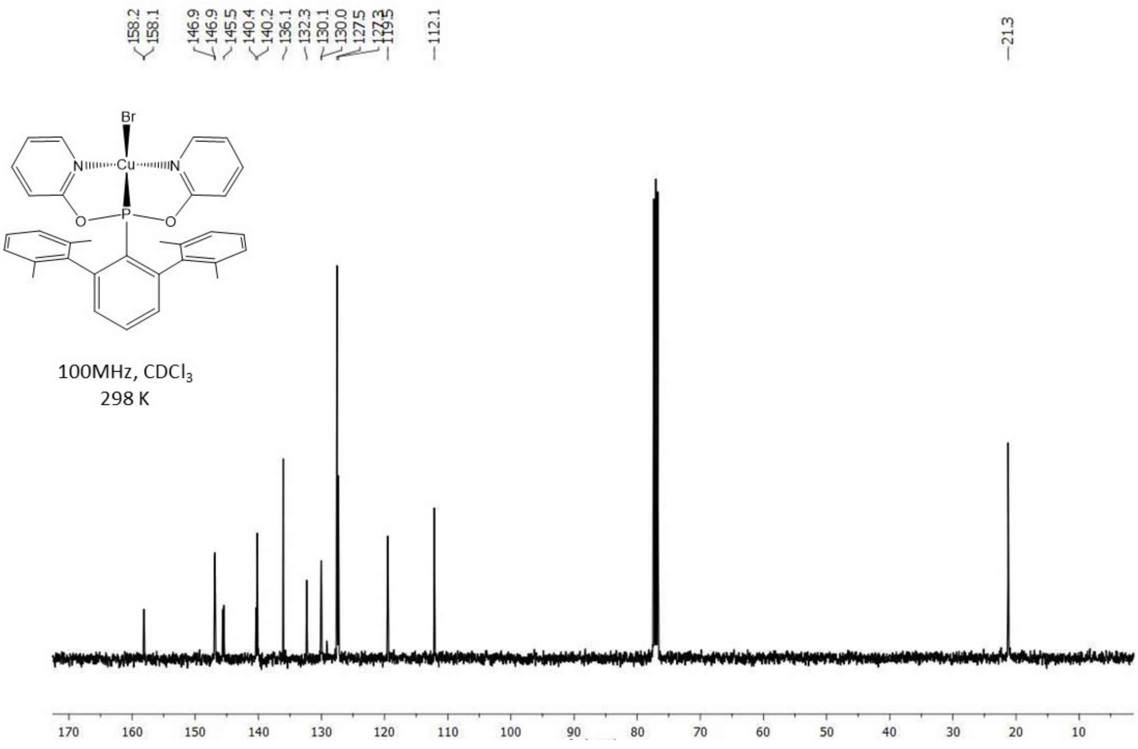

$^{31}P\{^{1}H\}$ NMR spectrum of 2a

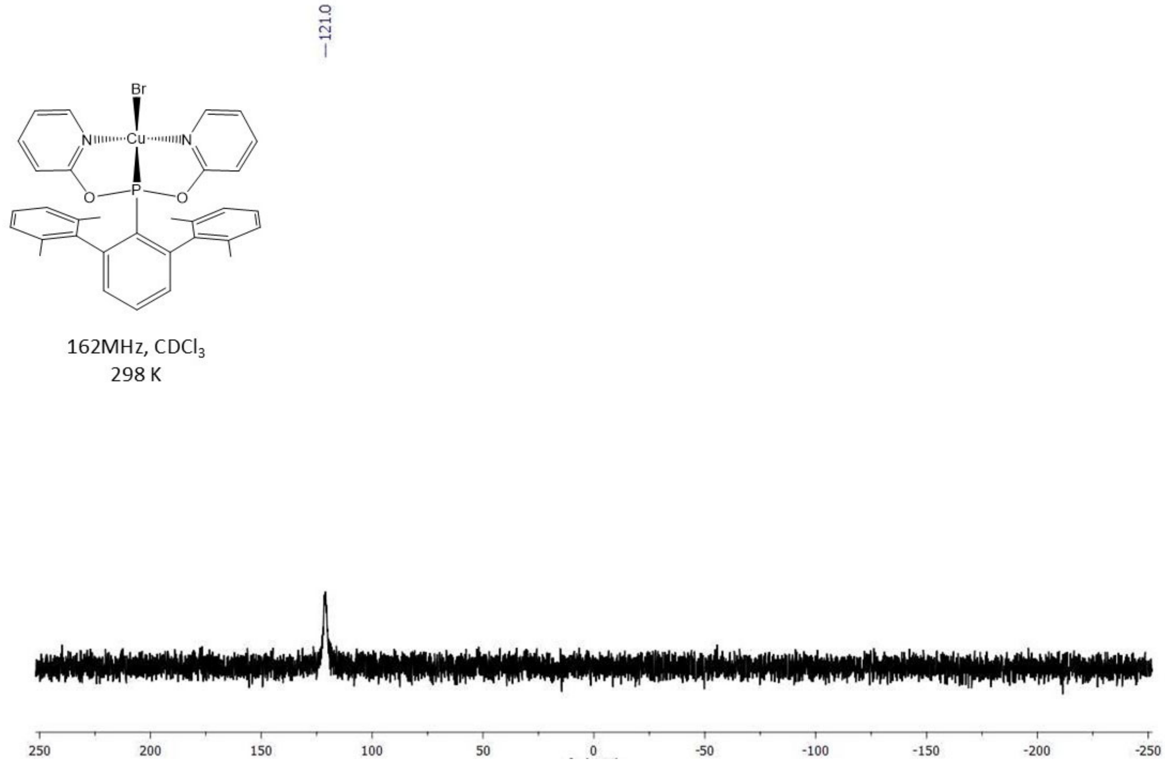

^{1}H NMR spectrum of 2b

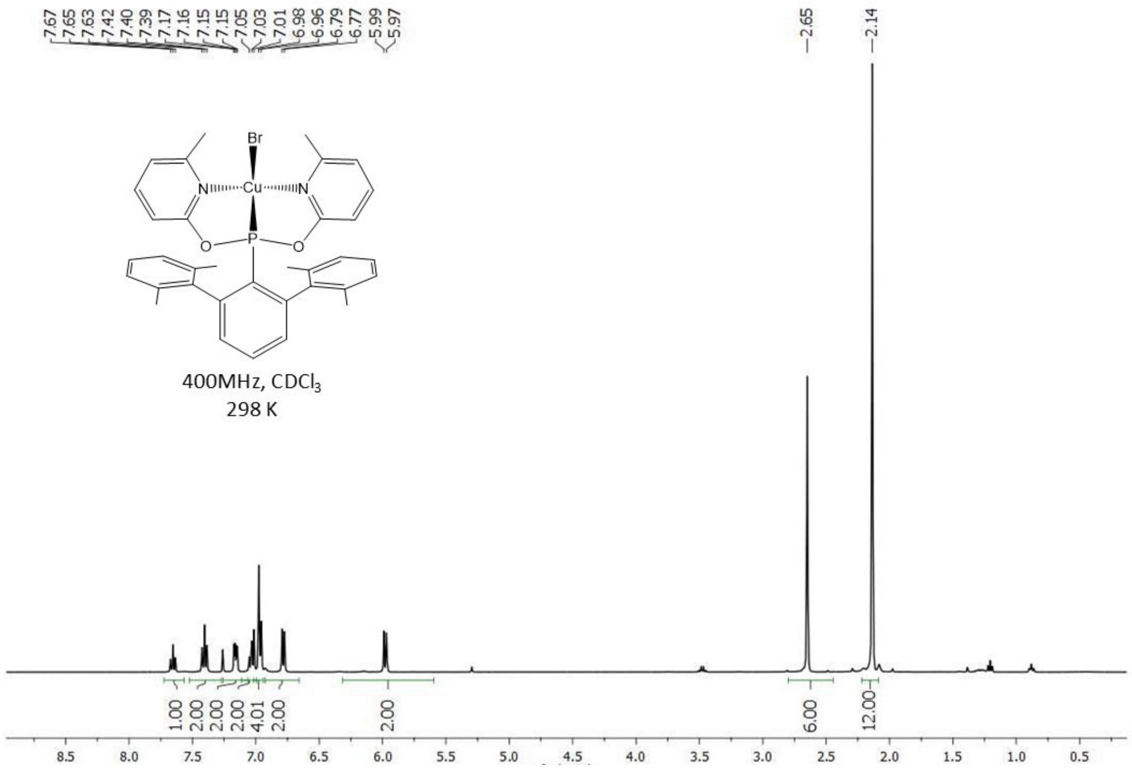

^{13}C{^{1}H} NMR spectrum of 2b

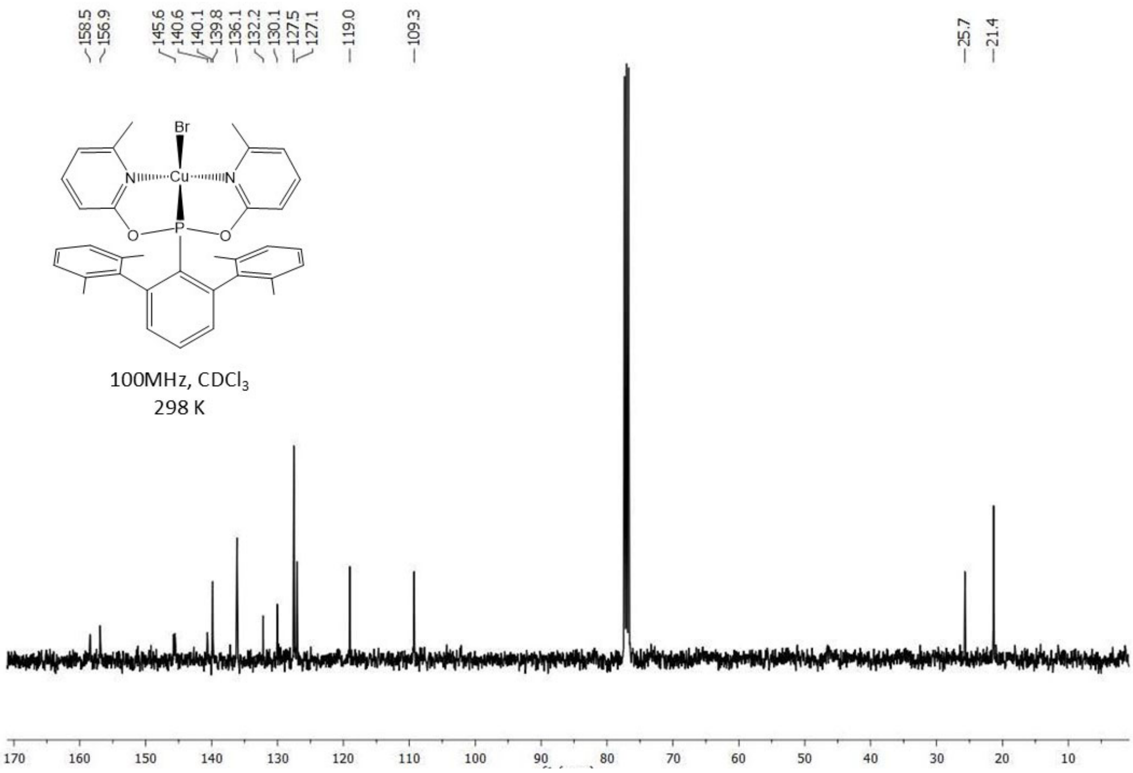

$^{31}P\{^{1}H\}$ NMR spectrum of 2b

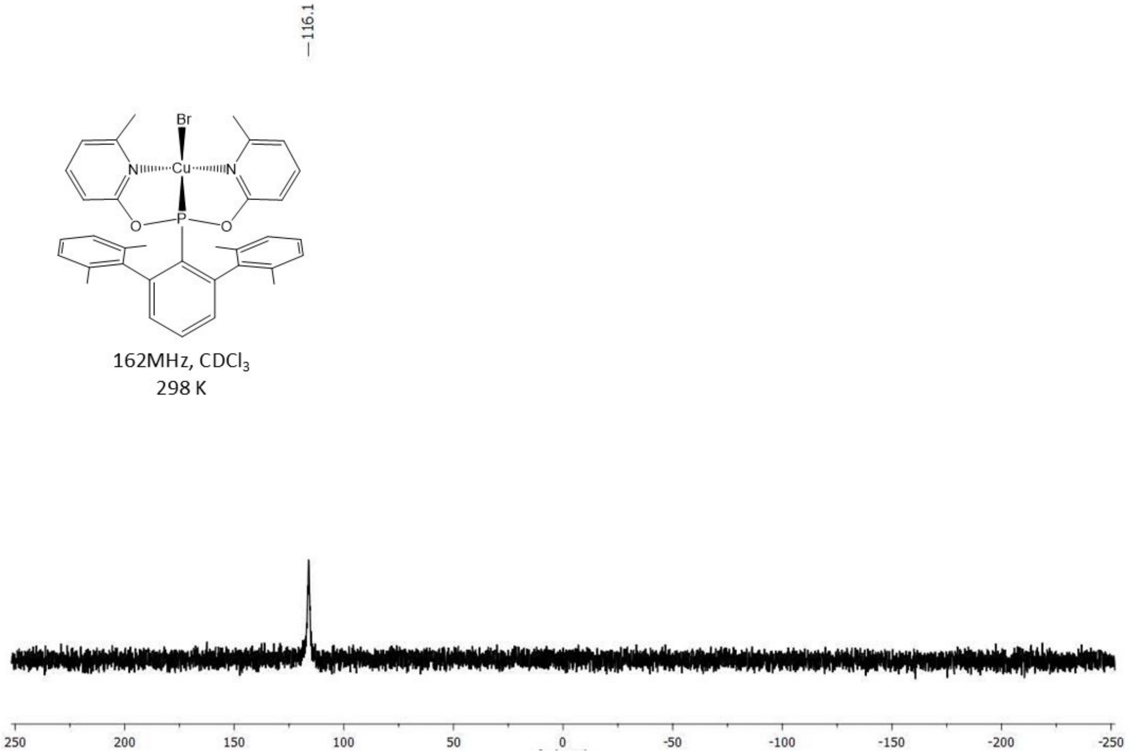

^{1}H NMR spectrum 2c

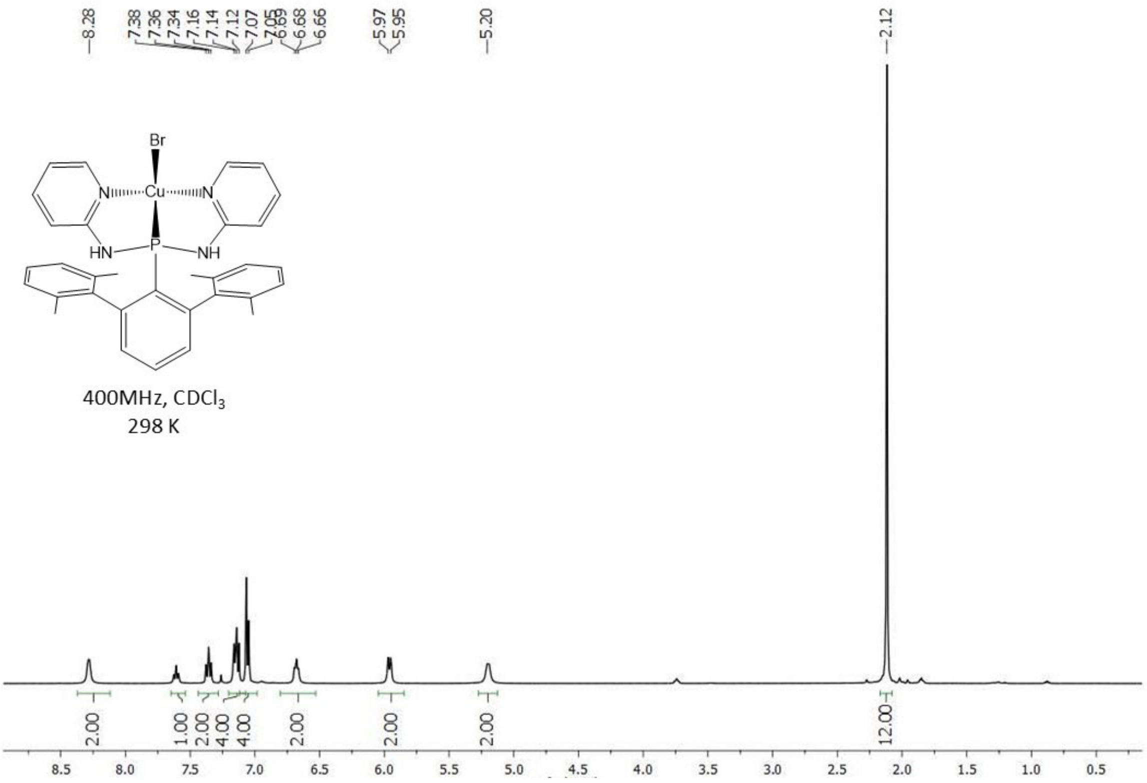

^{13}C{^{1}H} NMR spectrum of 2c

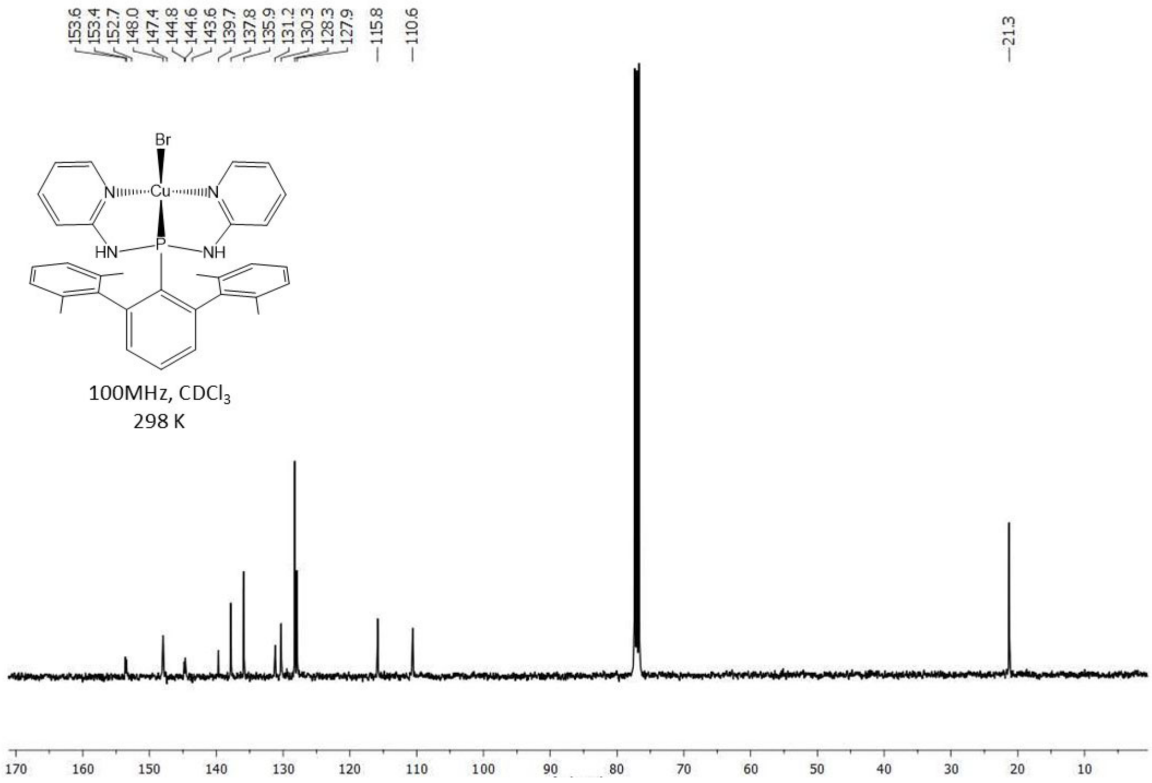

$^{31}P\{^1H\}$ NMR spectrum of 2c

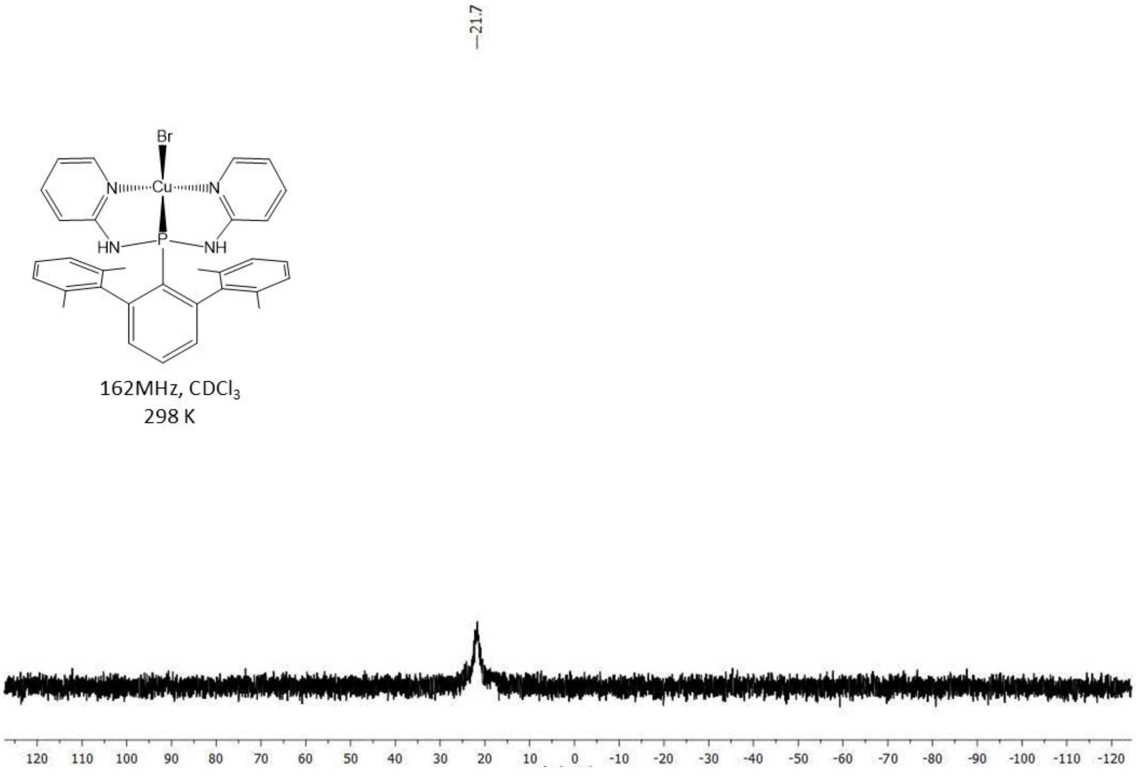

^{1}H NMR spectrum of 3a

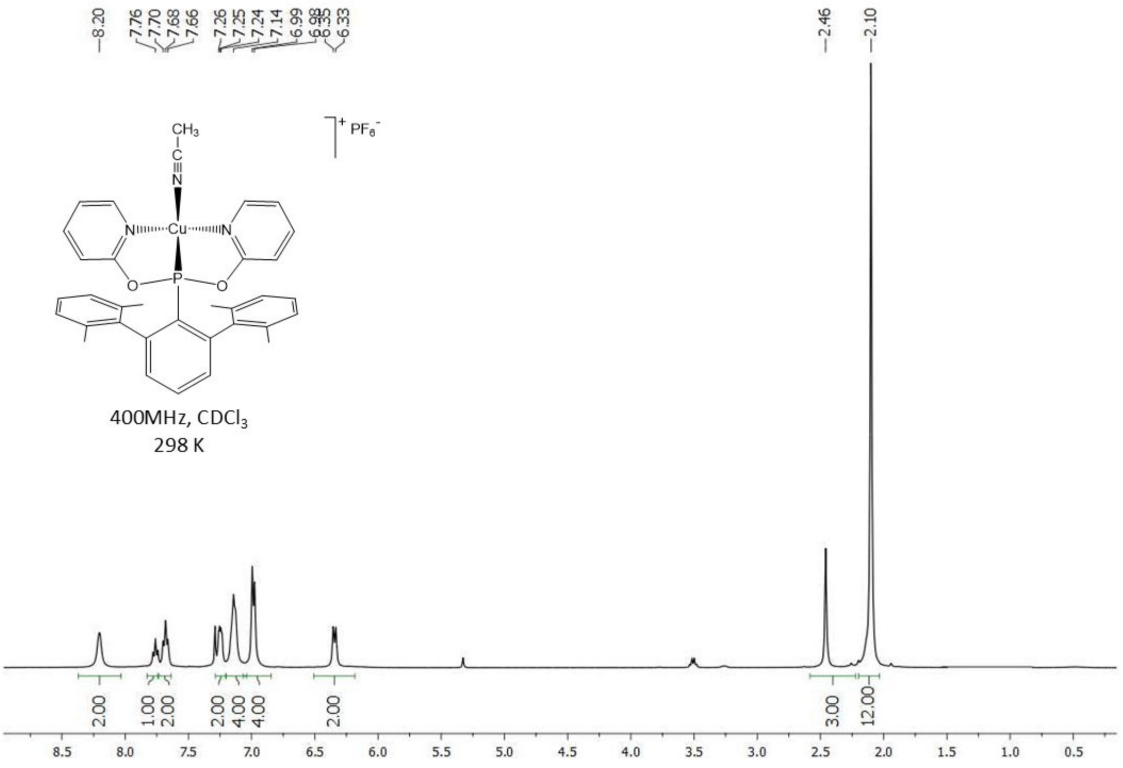

^{13}C{^{1}H} NMR spectrum of 3a

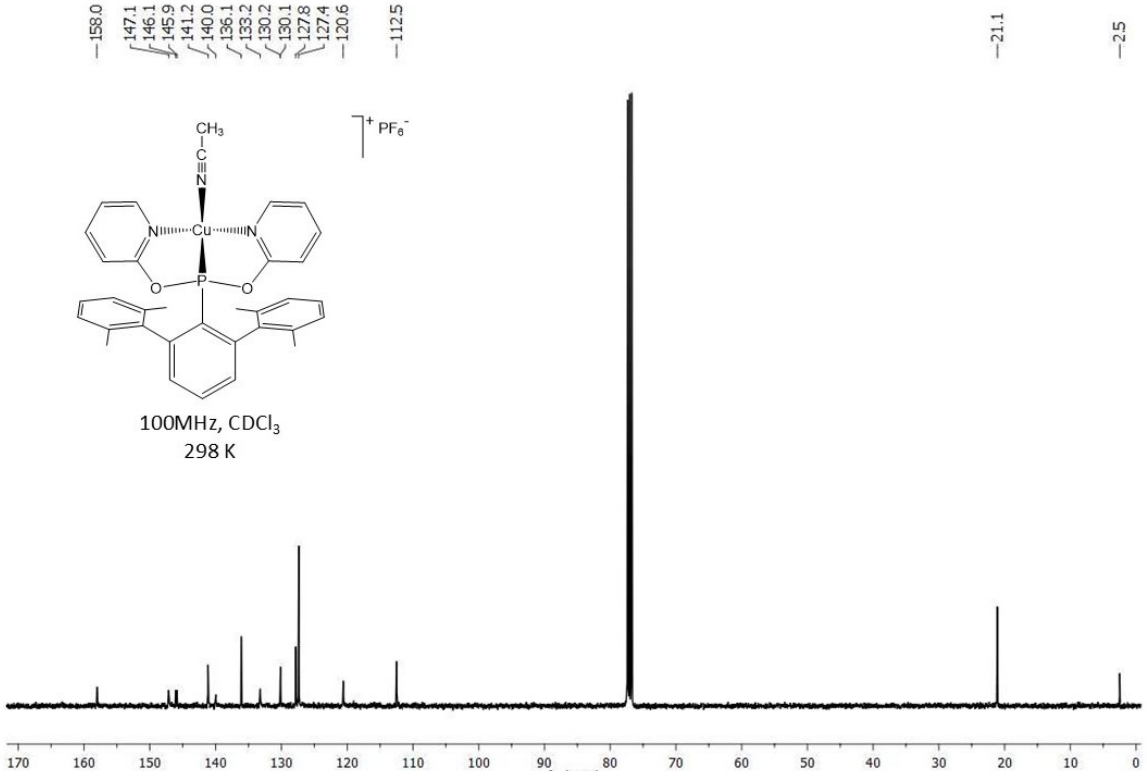

$^{31}P\{^1H\}$ NMR spectrum of 3a

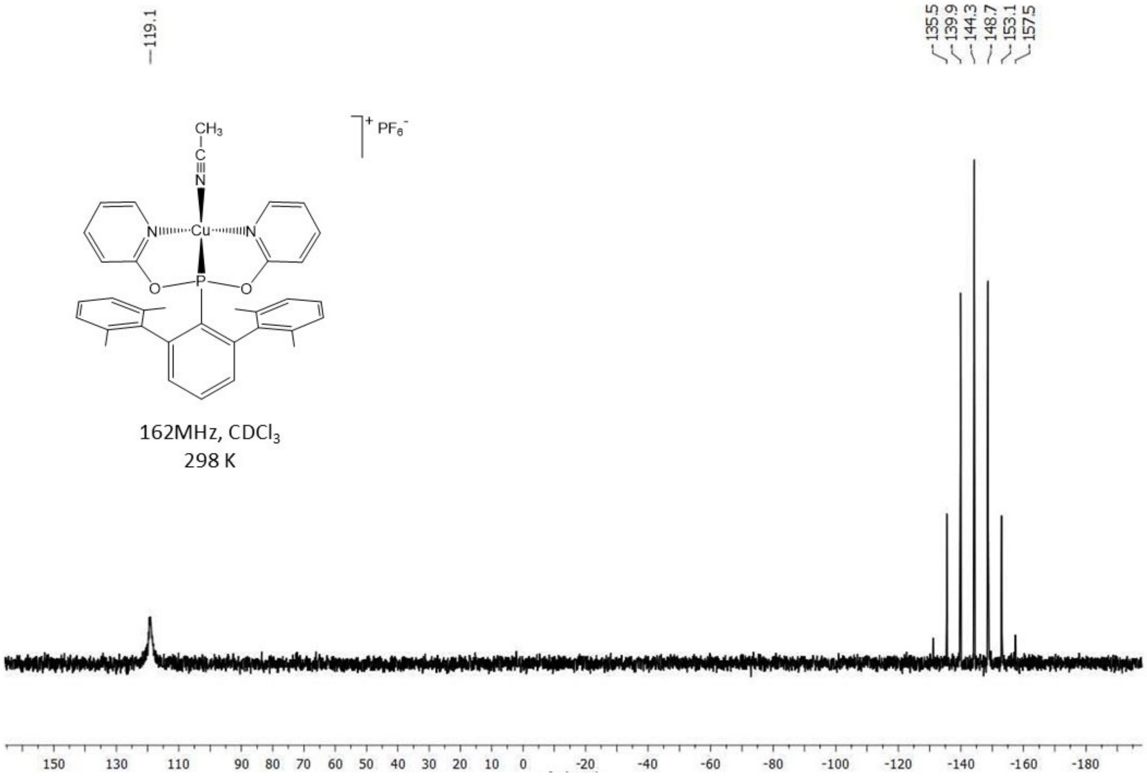

^{1}H NMR spectrum of 3b

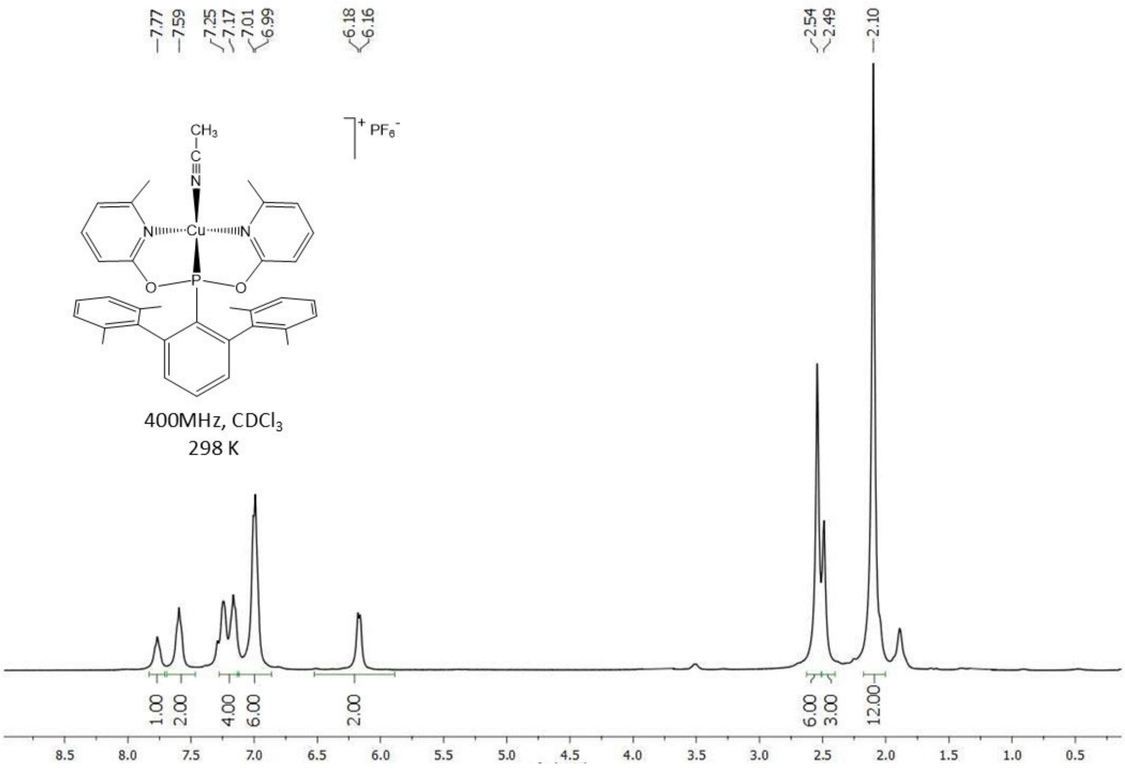

^{13}C{^{1}H} NMR spectrum of 3b

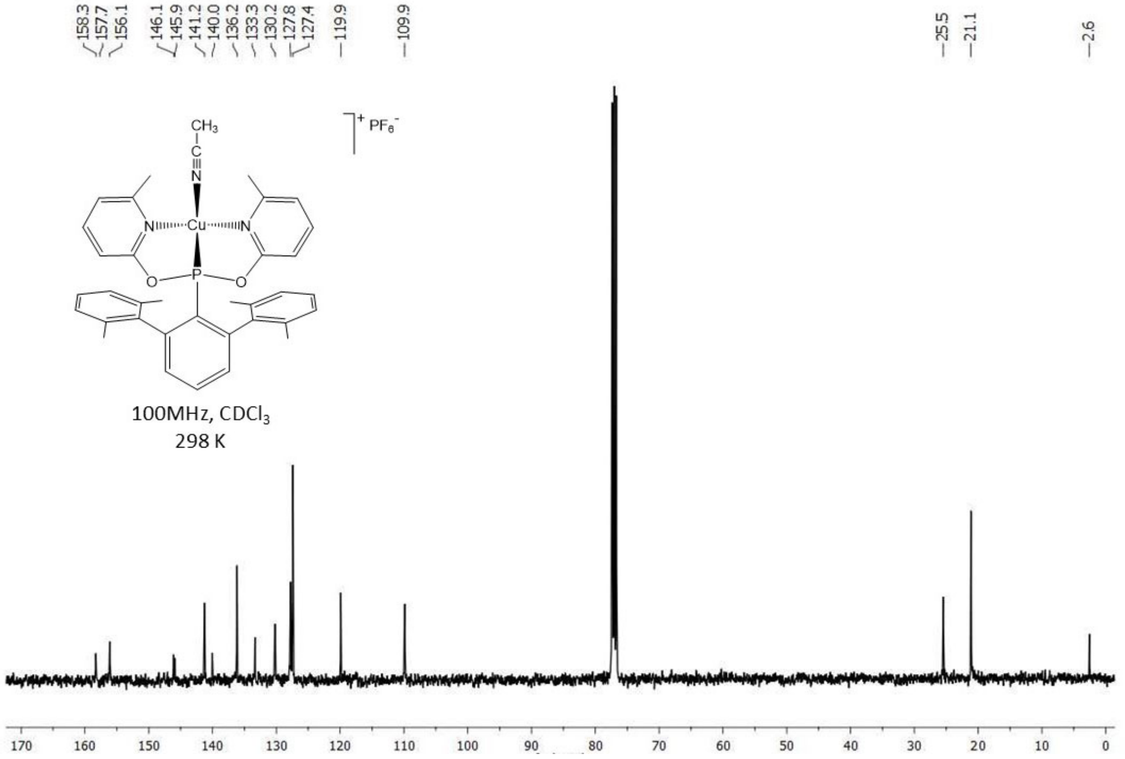

$^{31}P\{^{1}H\}$ NMR spectrum of 3b

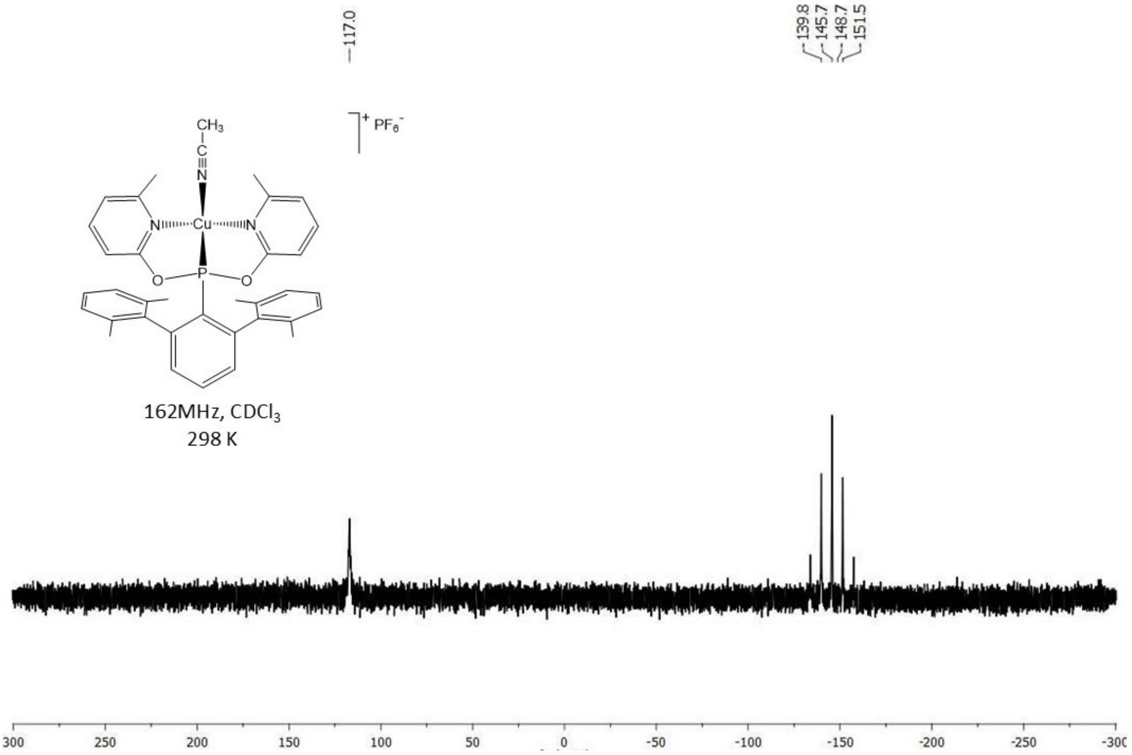

^{1}H NMR spectrum of 3c

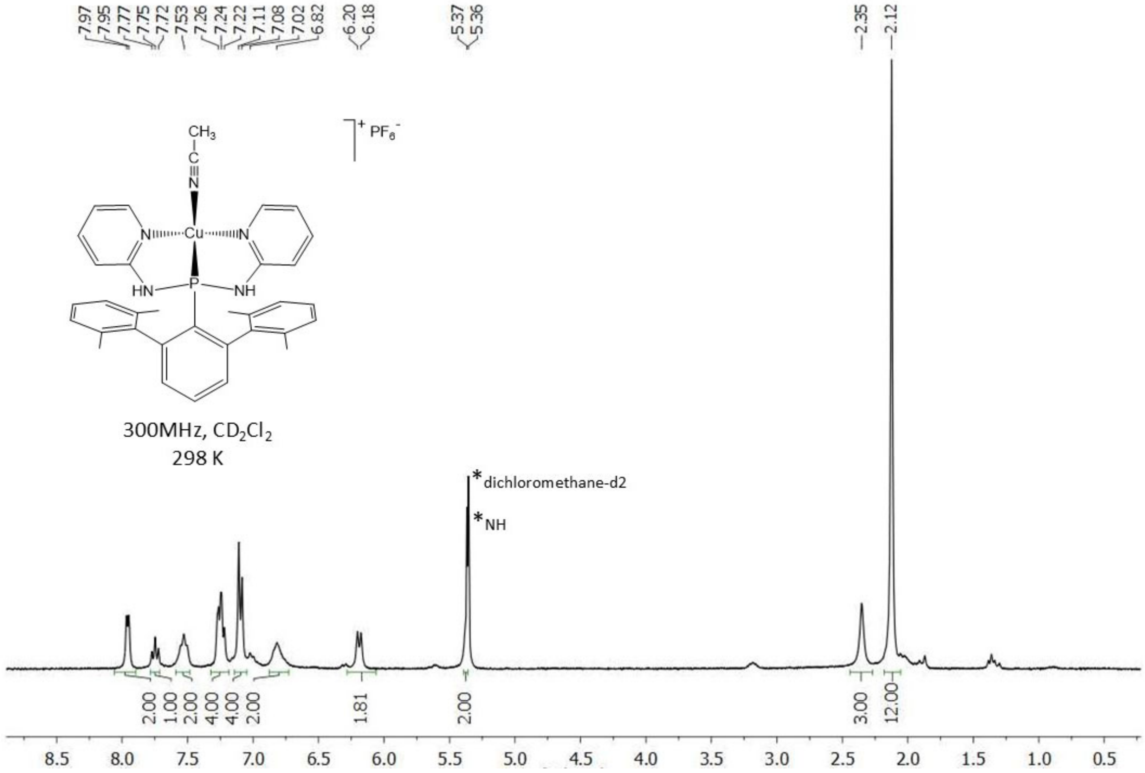

$^{13}C\{^{1}H\}$ NMR spectrum of 3c

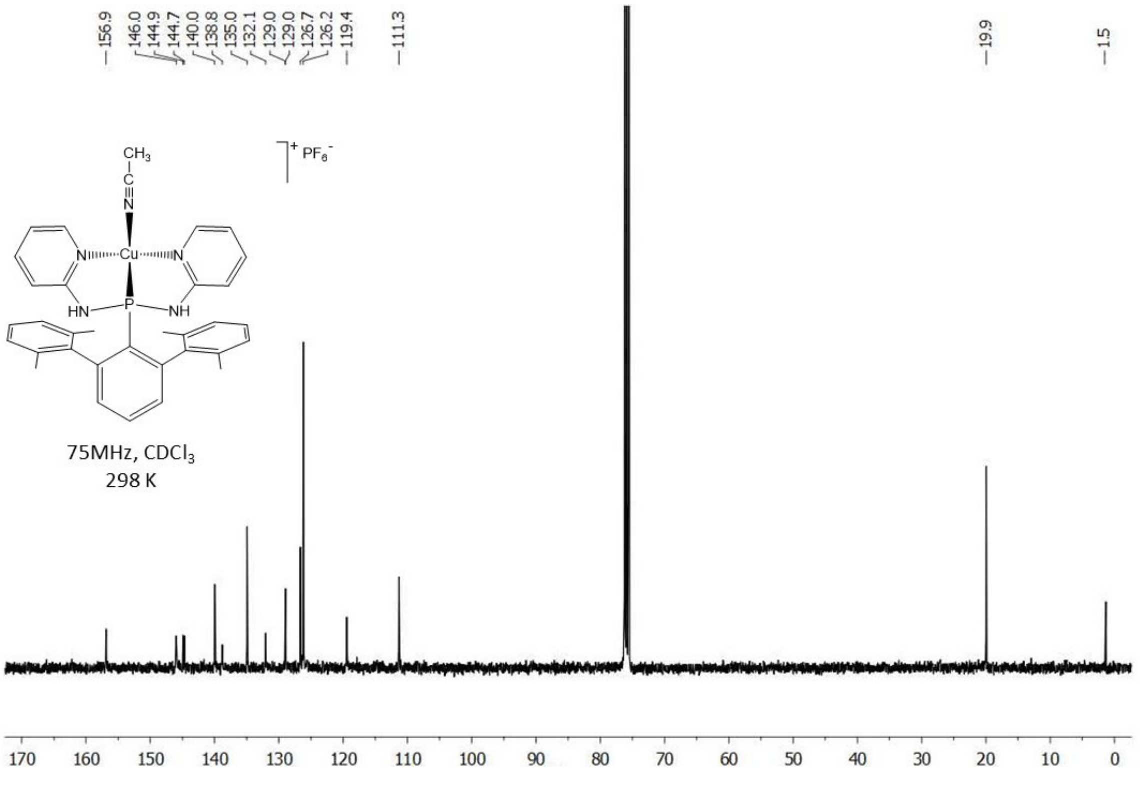

$^{31}P\{^1H\}$ NMR spectrum of 3c

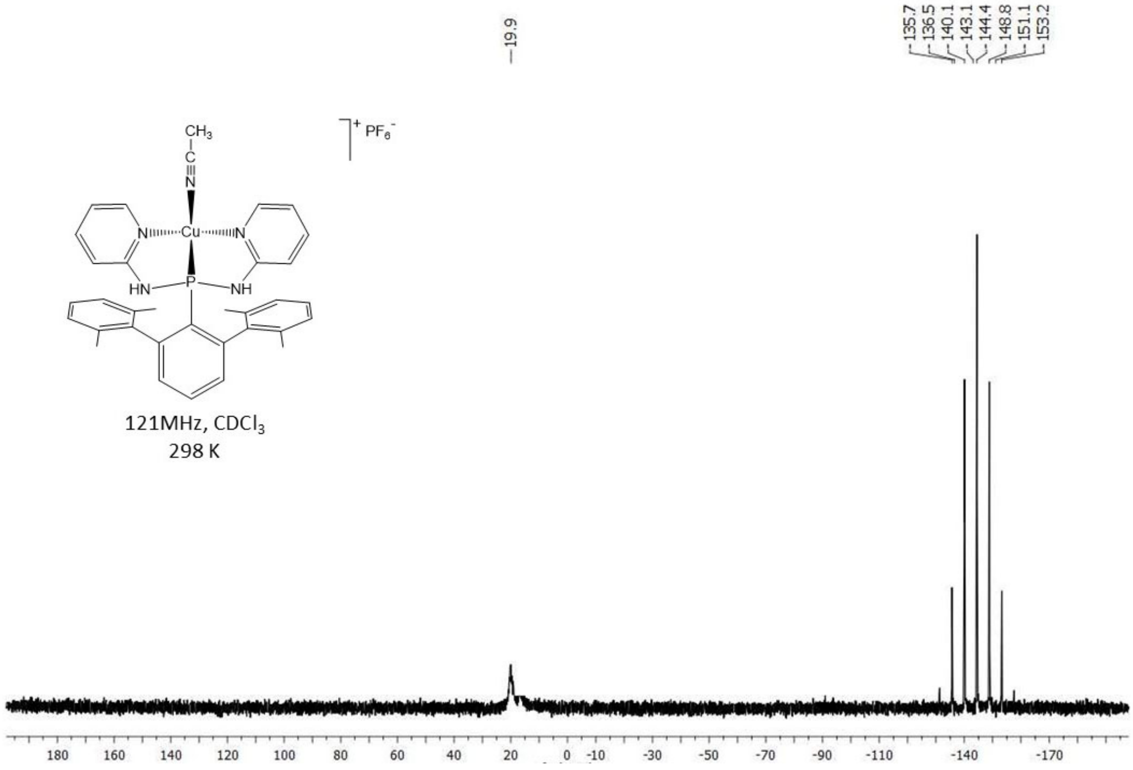

^{1}H NMR spectrum of diphenylsulfane (Scheme 5, a)

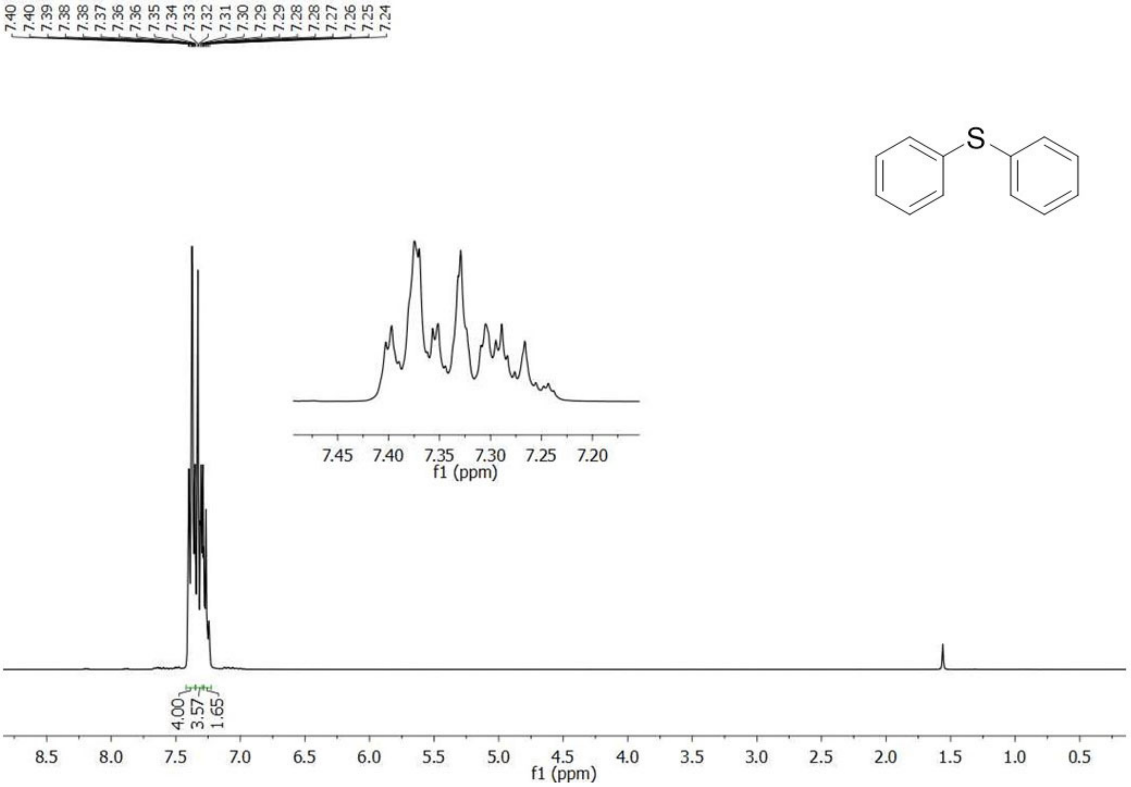

^{1}H NMR spectrum of (3,5-dimethylphenyl)(phenyl)sulfane (Scheme 5, b)

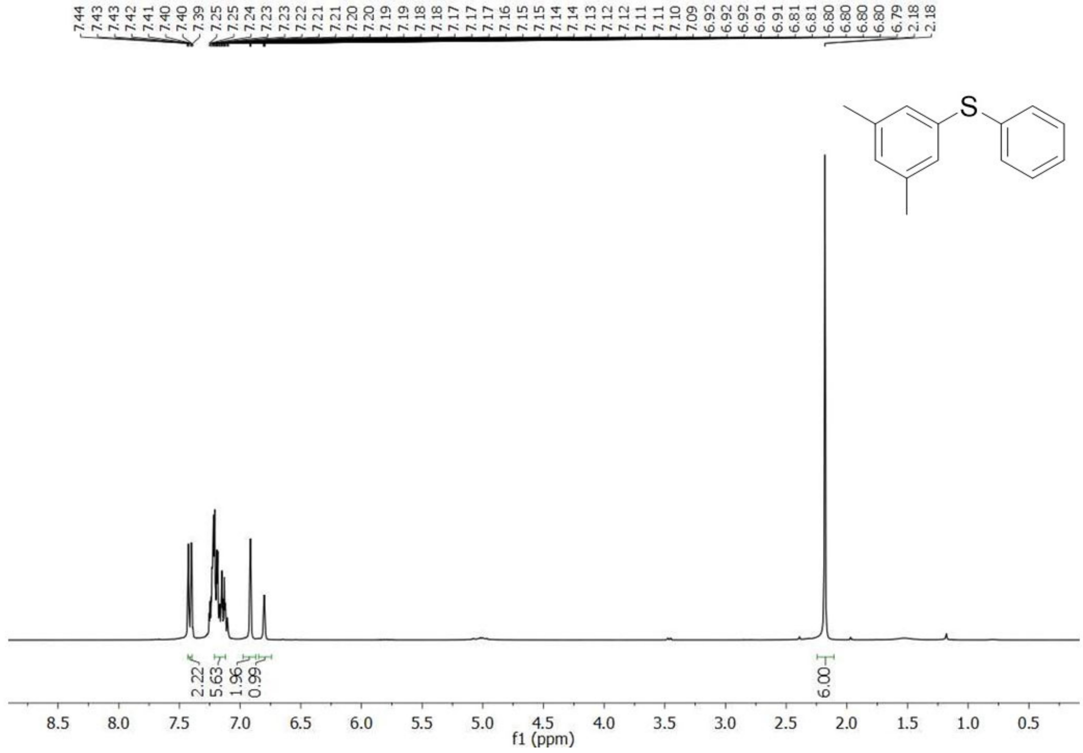

^{1}H NMR spectrum of phenyl(4-methylphenyl)sulfane (Scheme 5, c)

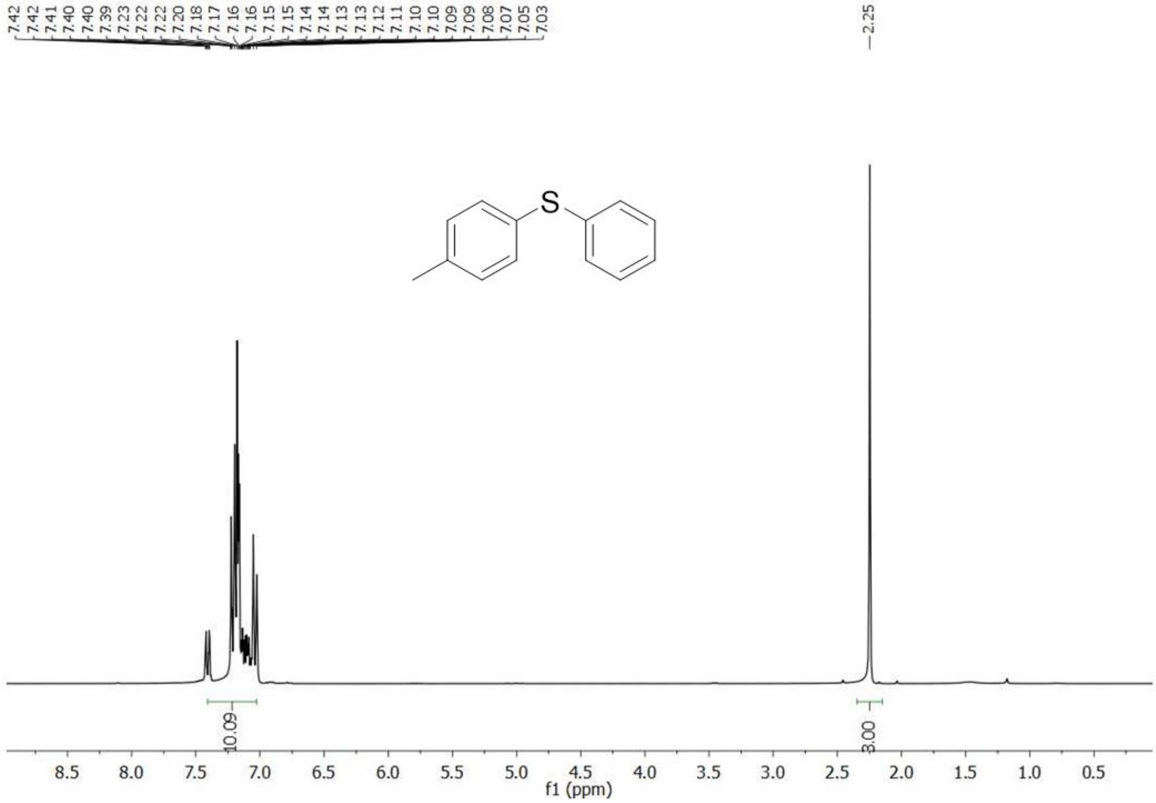

^{1}H NMR spectrum of (4-methoxyphenyl)(phenyl)sulfane (Scheme 5, d)

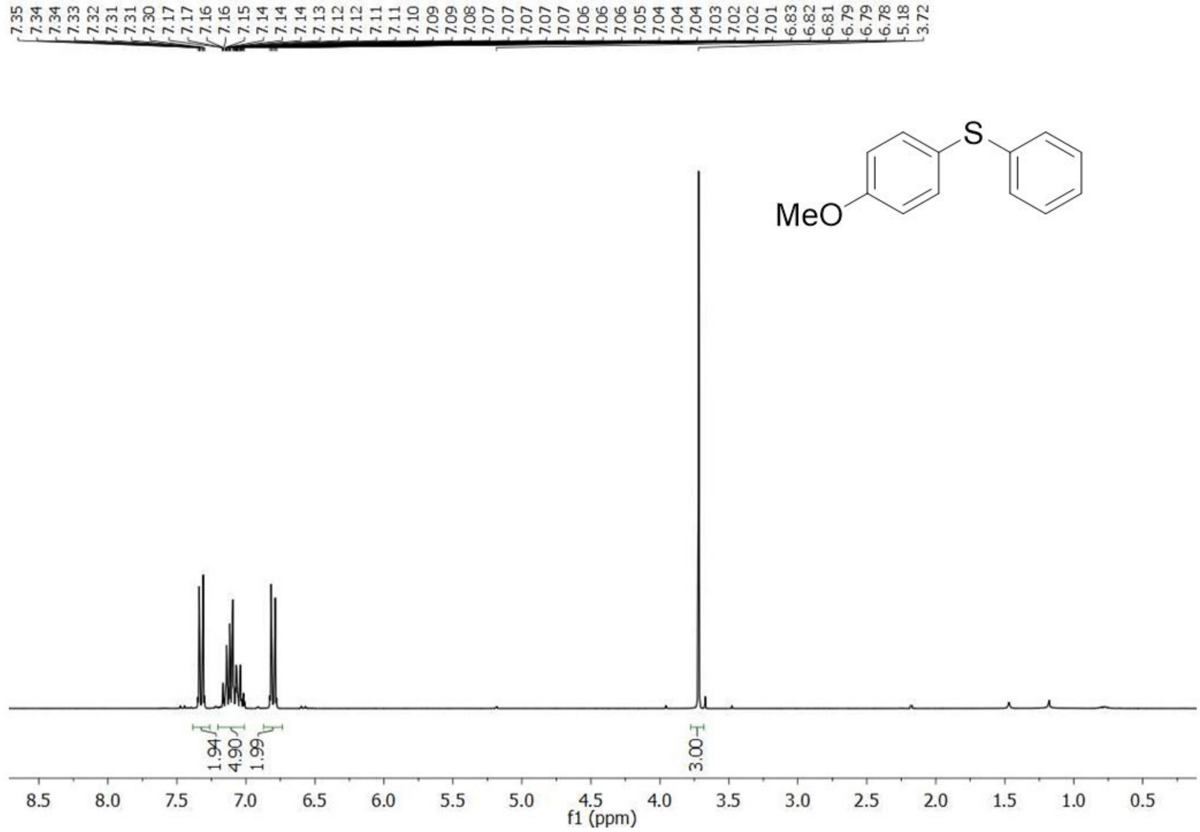

^{1}H NMR spectrum of phenyl(2-methylphenyl)sulfane (Scheme 5, e)

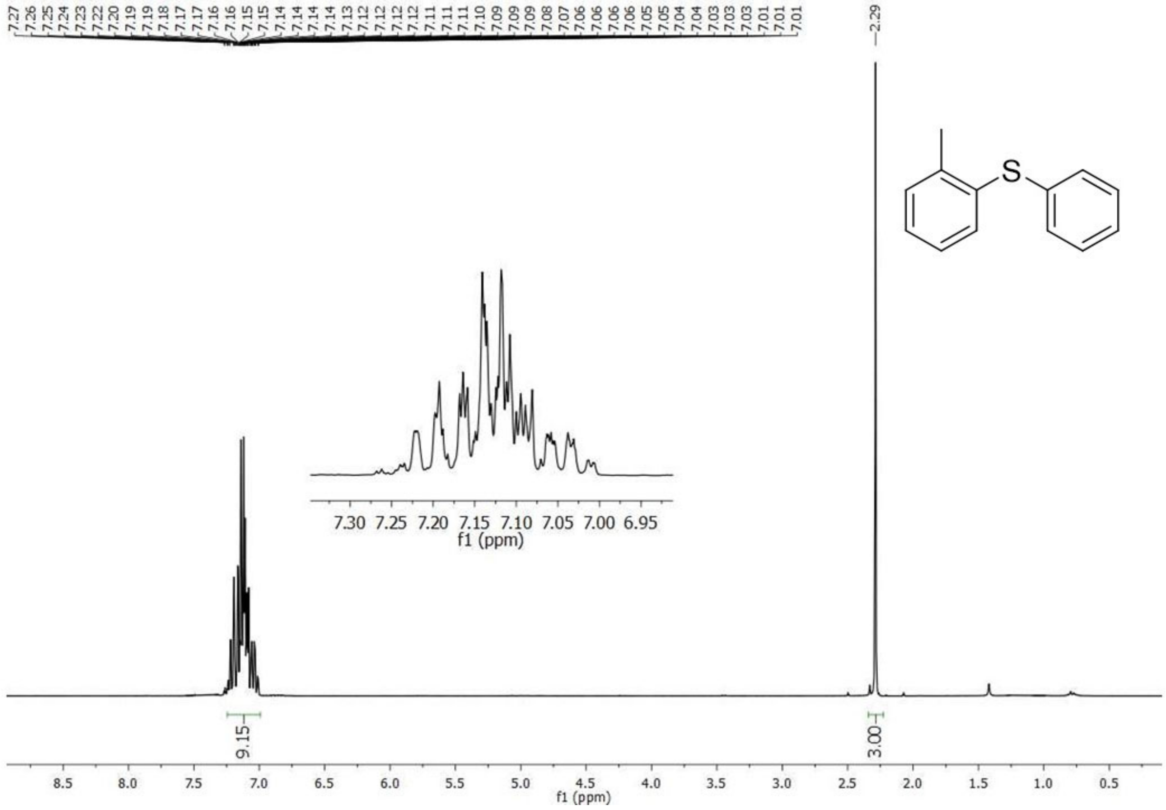

^{1}H NMR spectrum of o-tolyl(p-tolyl)sulfane (Scheme 5, f)

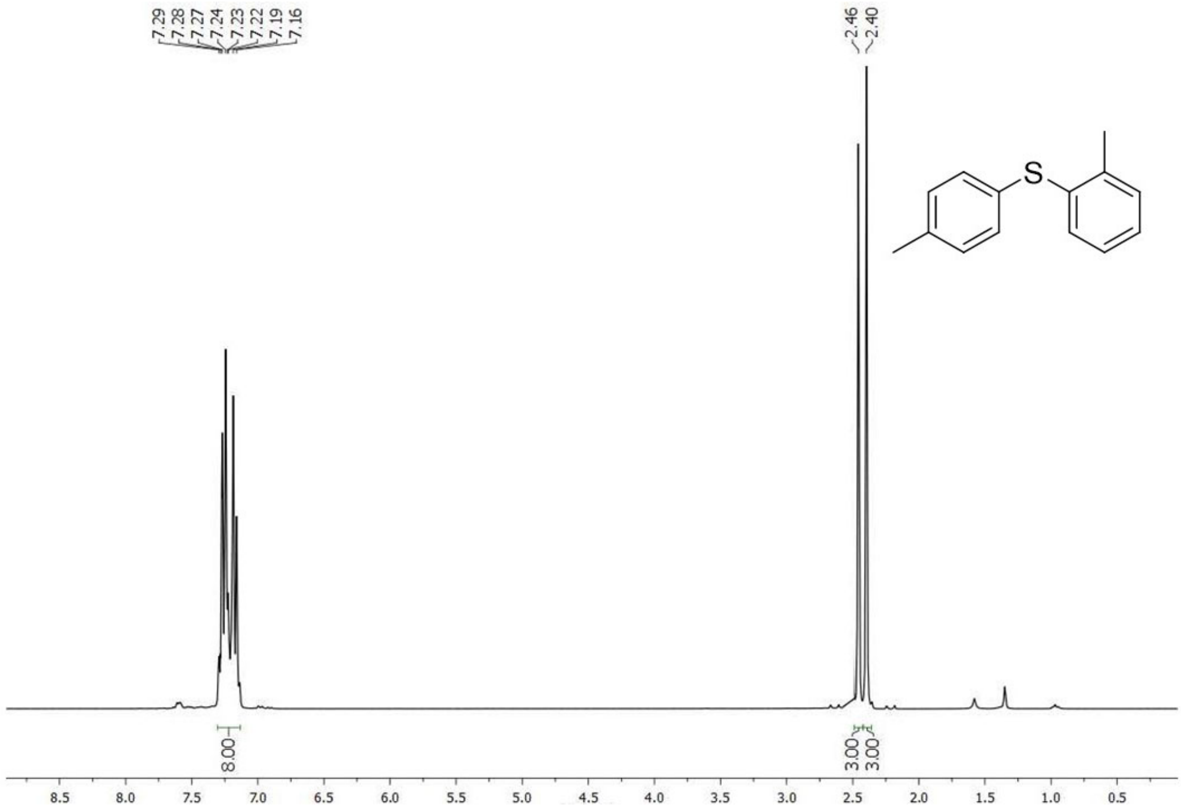

^{1}H NMR spectrum of (4-Methoxyphenyl)(*p*-tolyl)sulfane (Scheme 5, g)

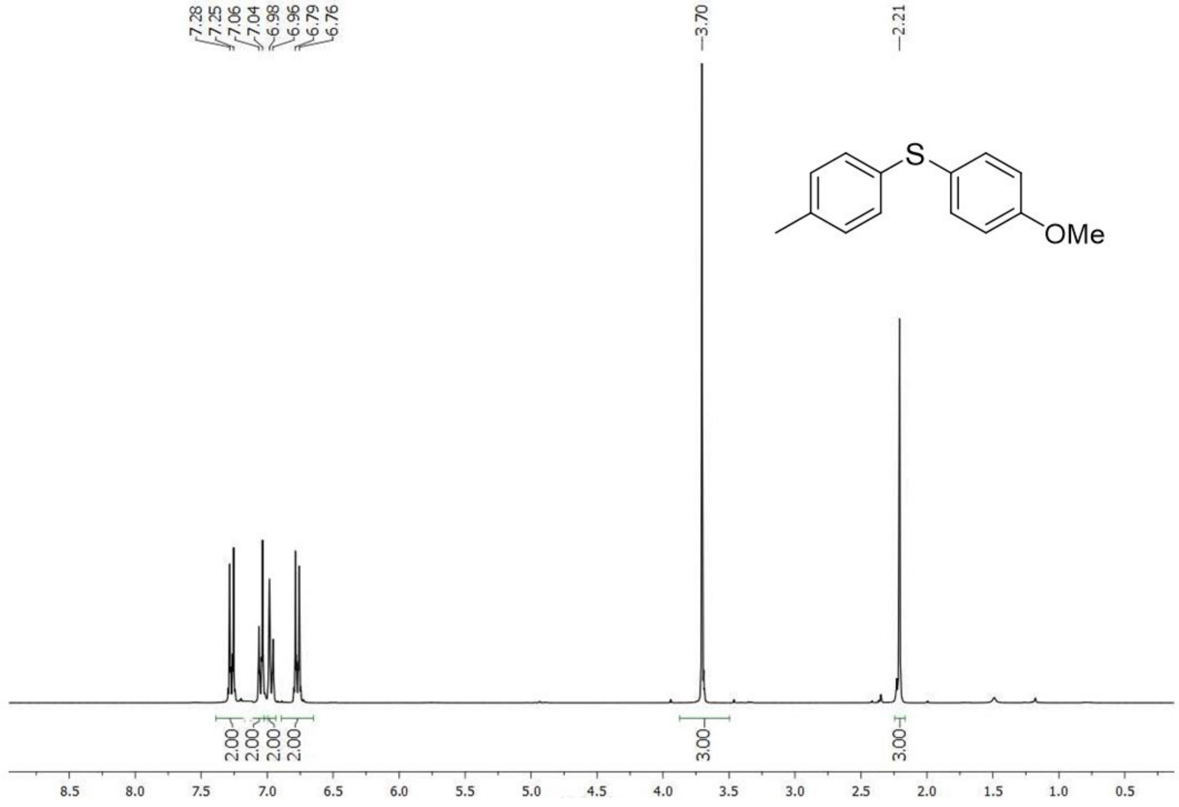

^{1}H NMR spectrum of (4-chlorophenyl)(phenyl)sulfane (Scheme 5, h)

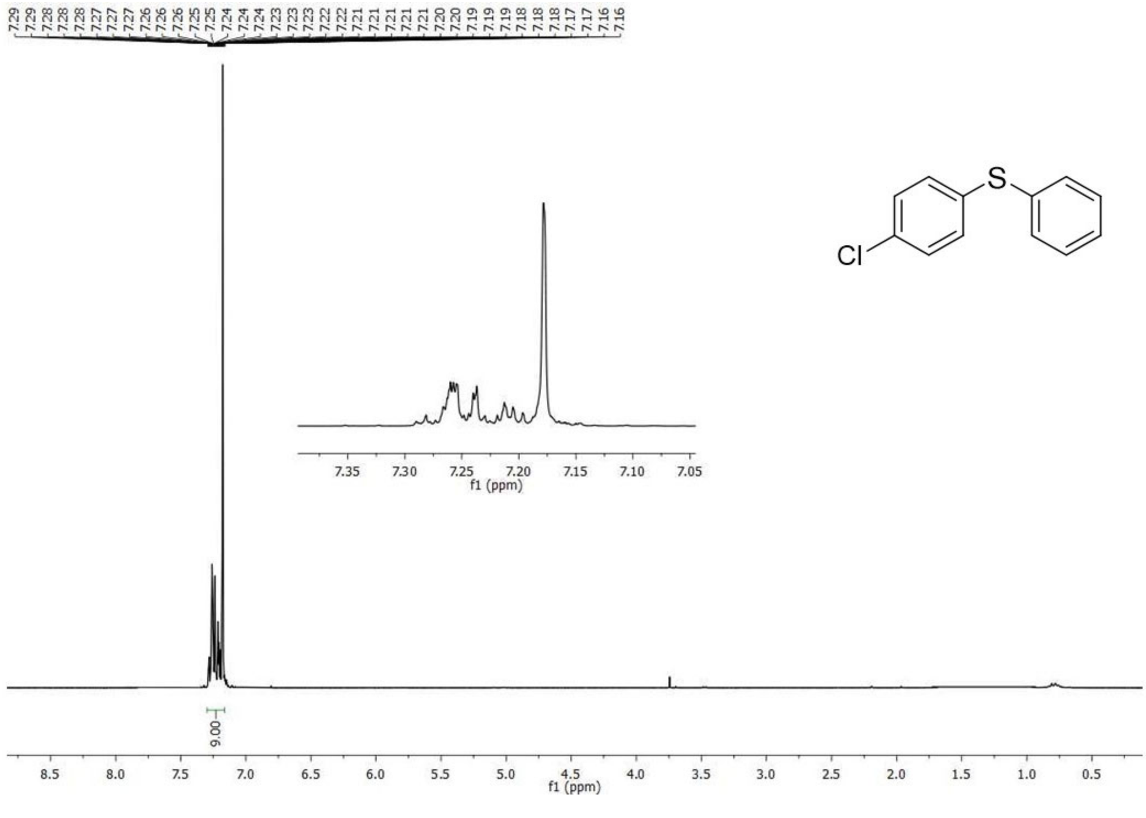

^{1}H NMR spectrum of (4-bromophenyl)phenylsulfane (Scheme 5, i)

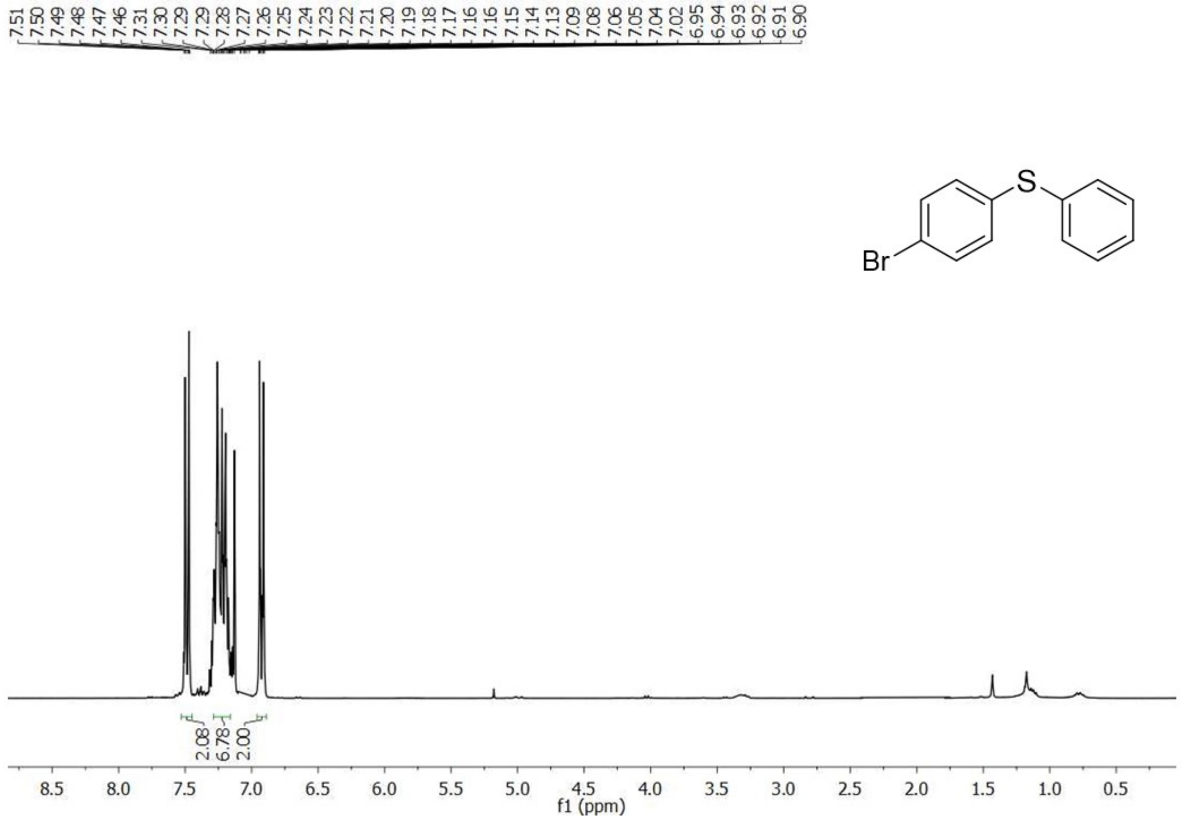

^{1}H NMR spectrum of 4-(phenylthiol)pyridine (Scheme 5, j)

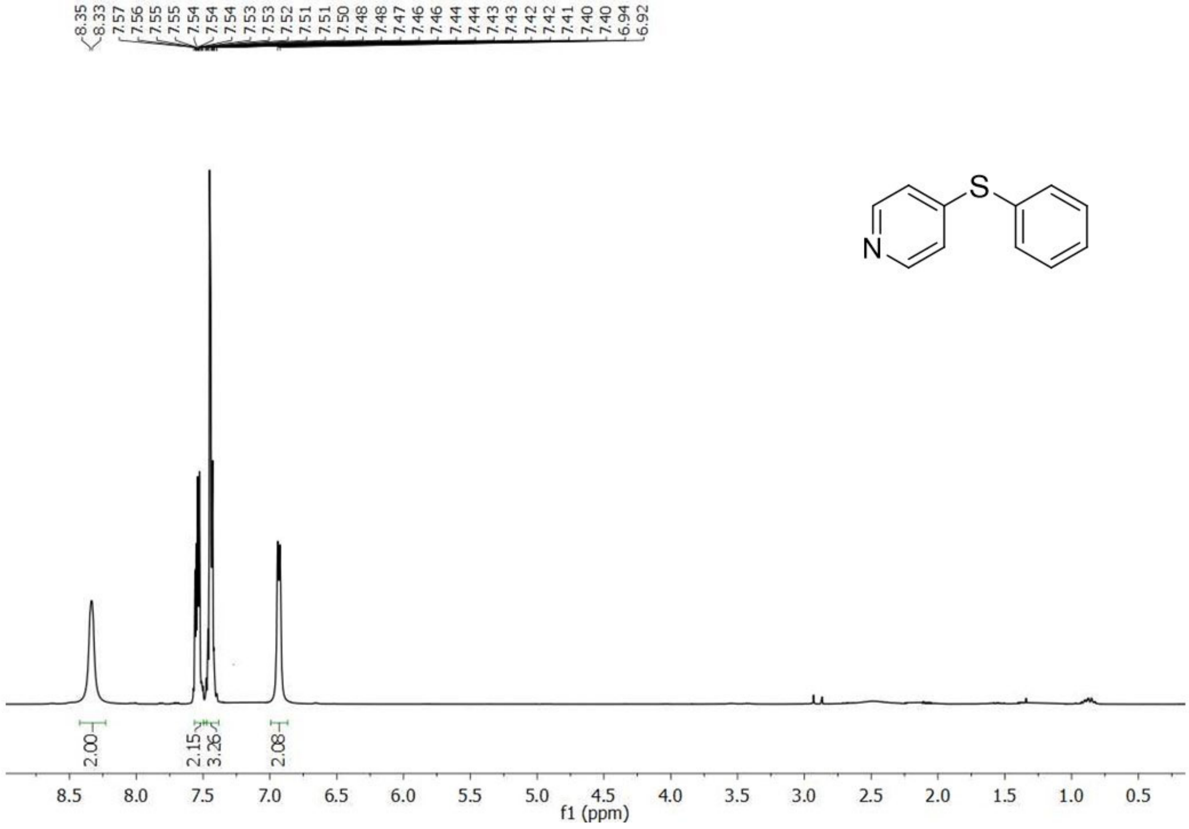

^{1}H NMR spectrum of 2-(4-Methylphenylthio)naphthalene (Scheme 5, k)

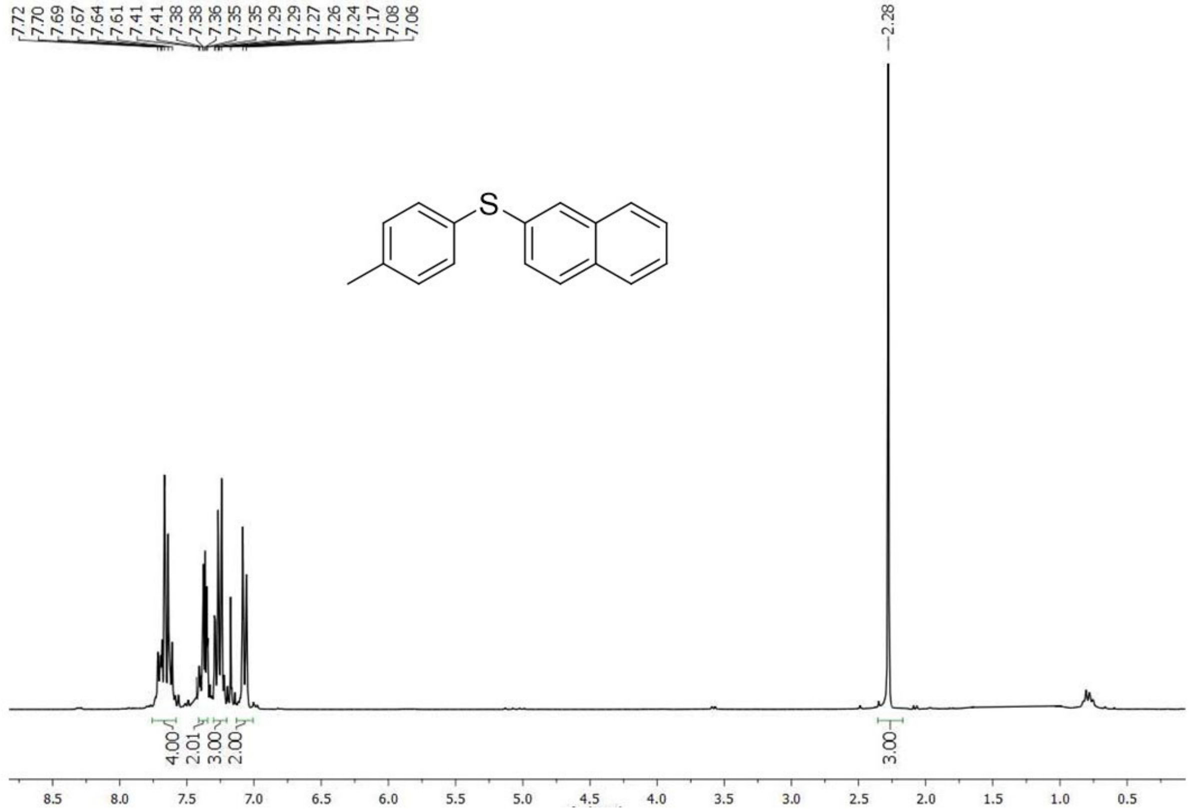

^{1}H NMR spectrum of (4-fluorophenyl)(4-tolyl)sulfane (Scheme 5, l)

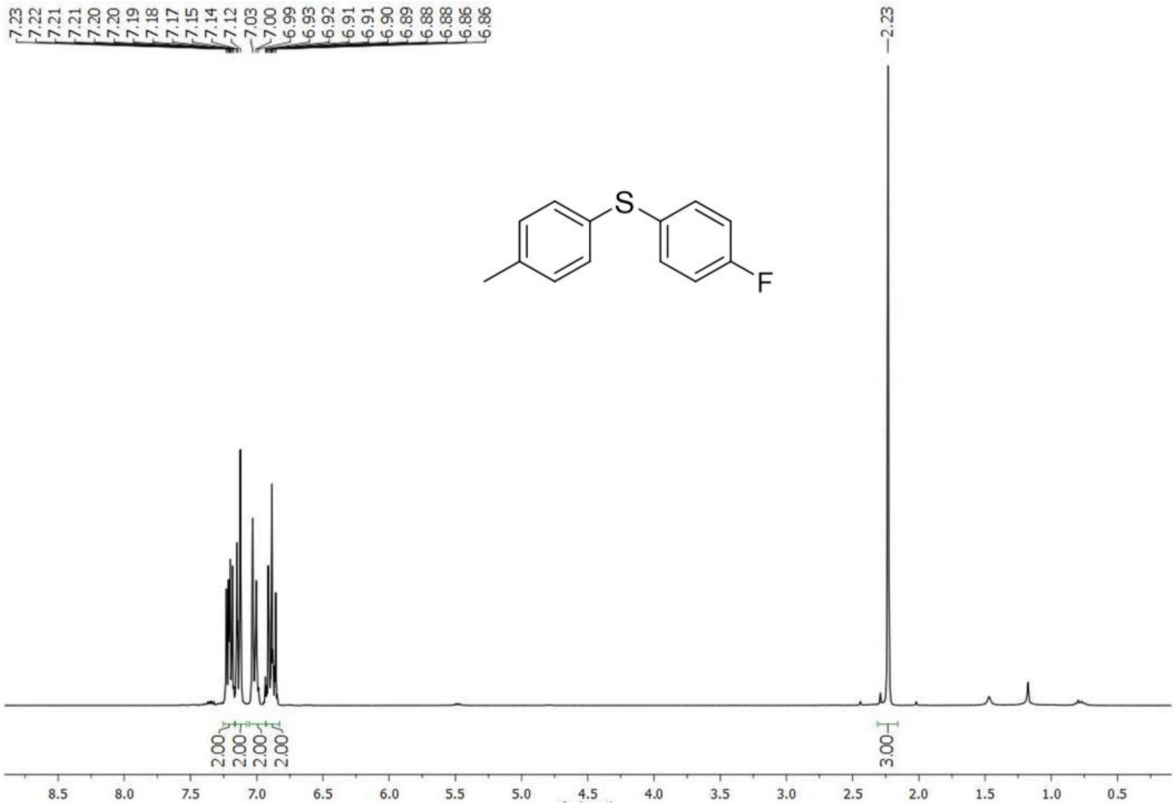

^{1}H NMR spectrum of 4-(4-methylphenylthio)aniline (Scheme 5, m)

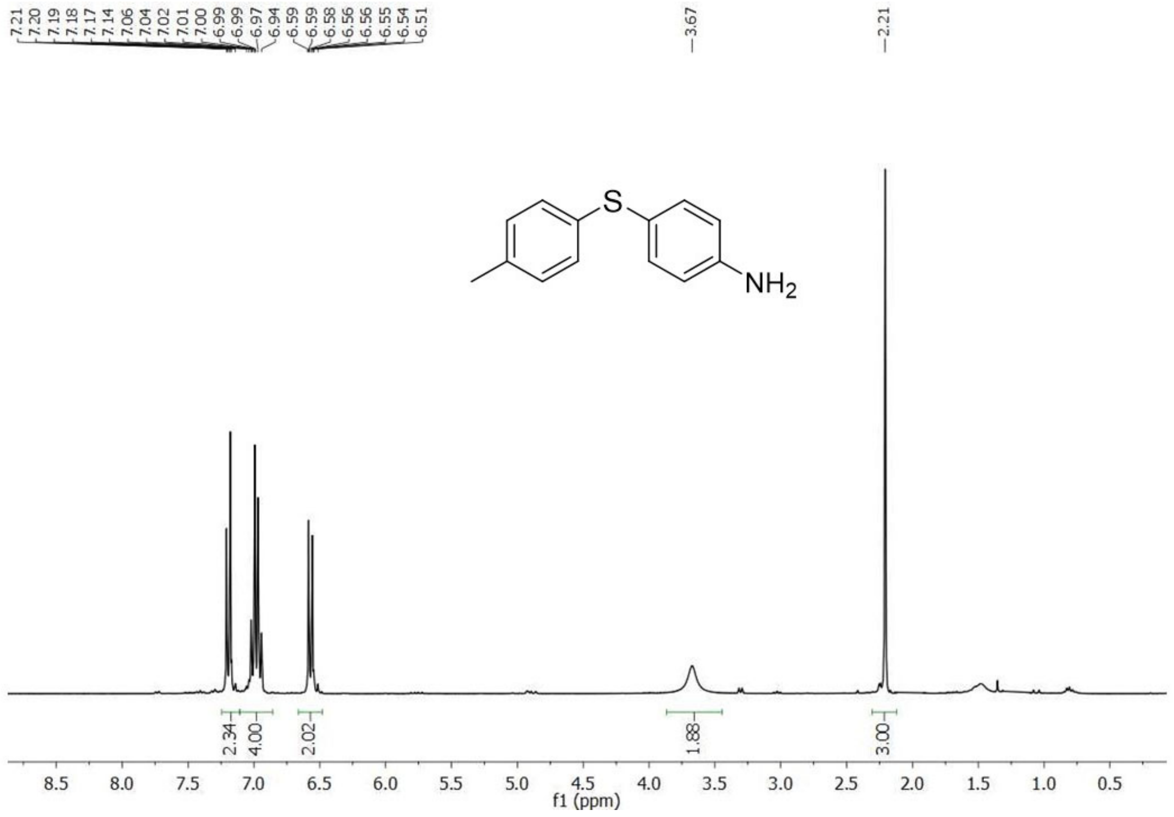

^{1}H NMR spectrum of 4-(p-tolylthio)phenol (Scheme 5, n)

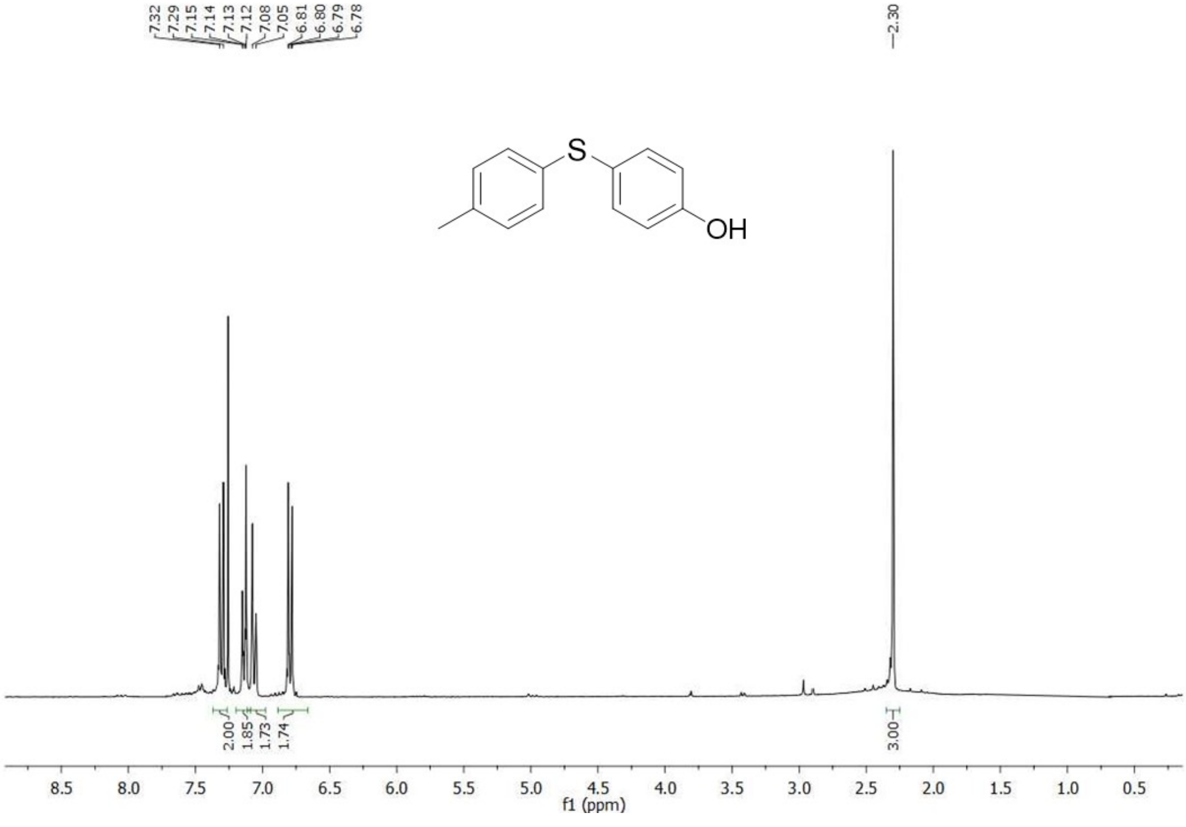

^{1}H NMR spectrum of cyclohexyl(phenyl)sulfane (Scheme 5, o)

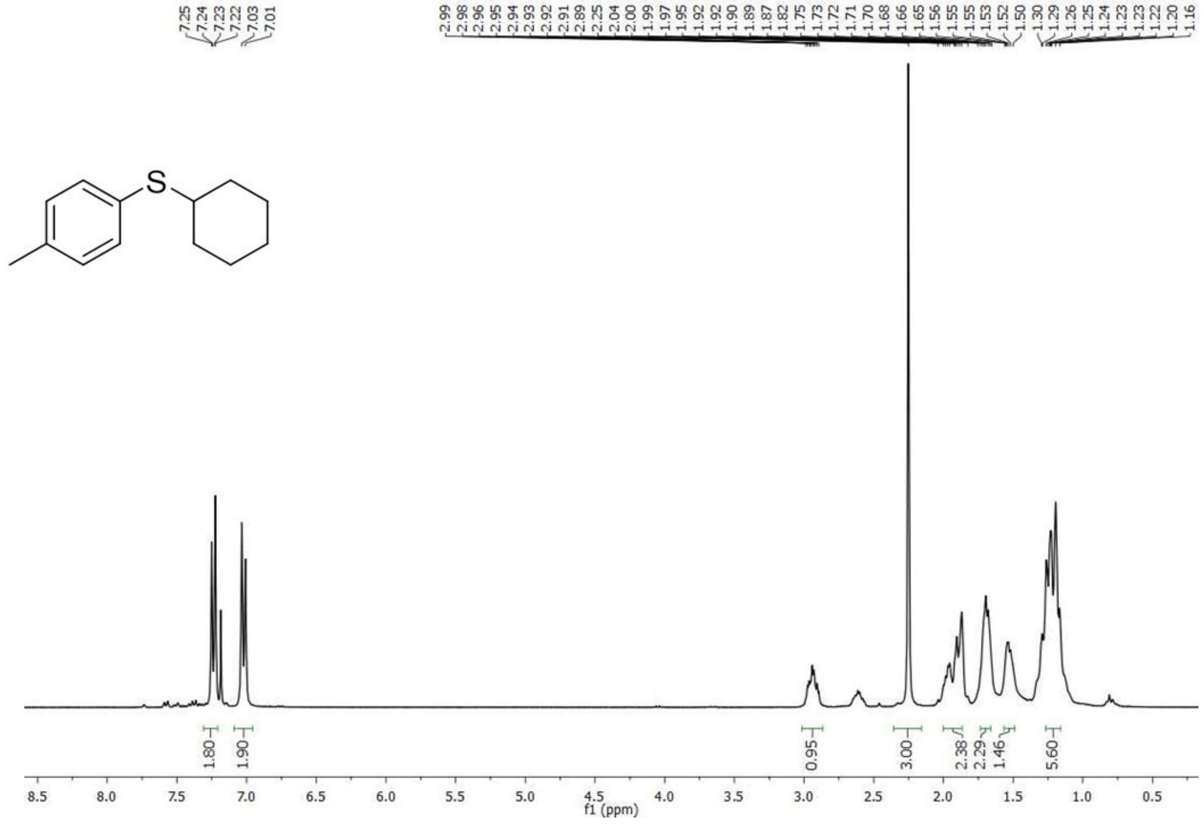

^{1}H NMR spectrum of 4-(cyclohexylthio)pyridine (Scheme 5, p)

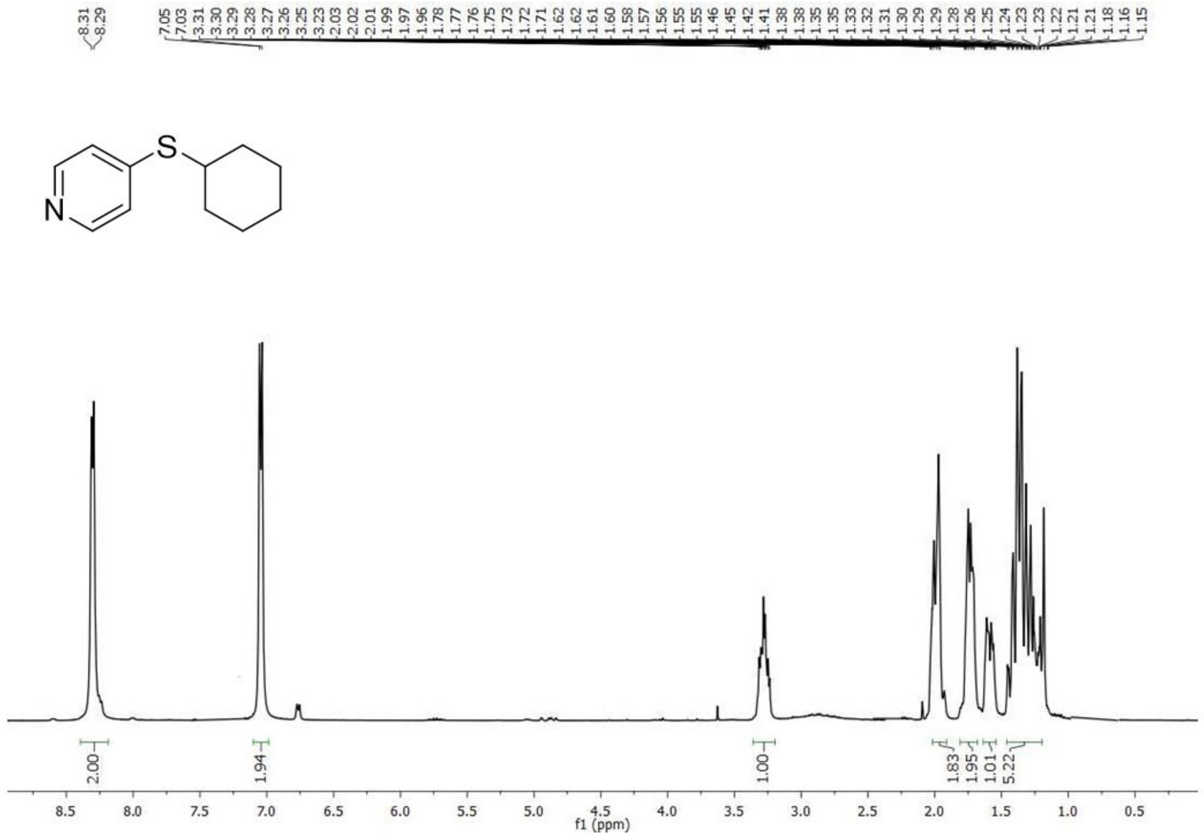

^{1}H NMR spectrum of tert-butyl(p-tolyl)sulfane (Scheme 5, q)

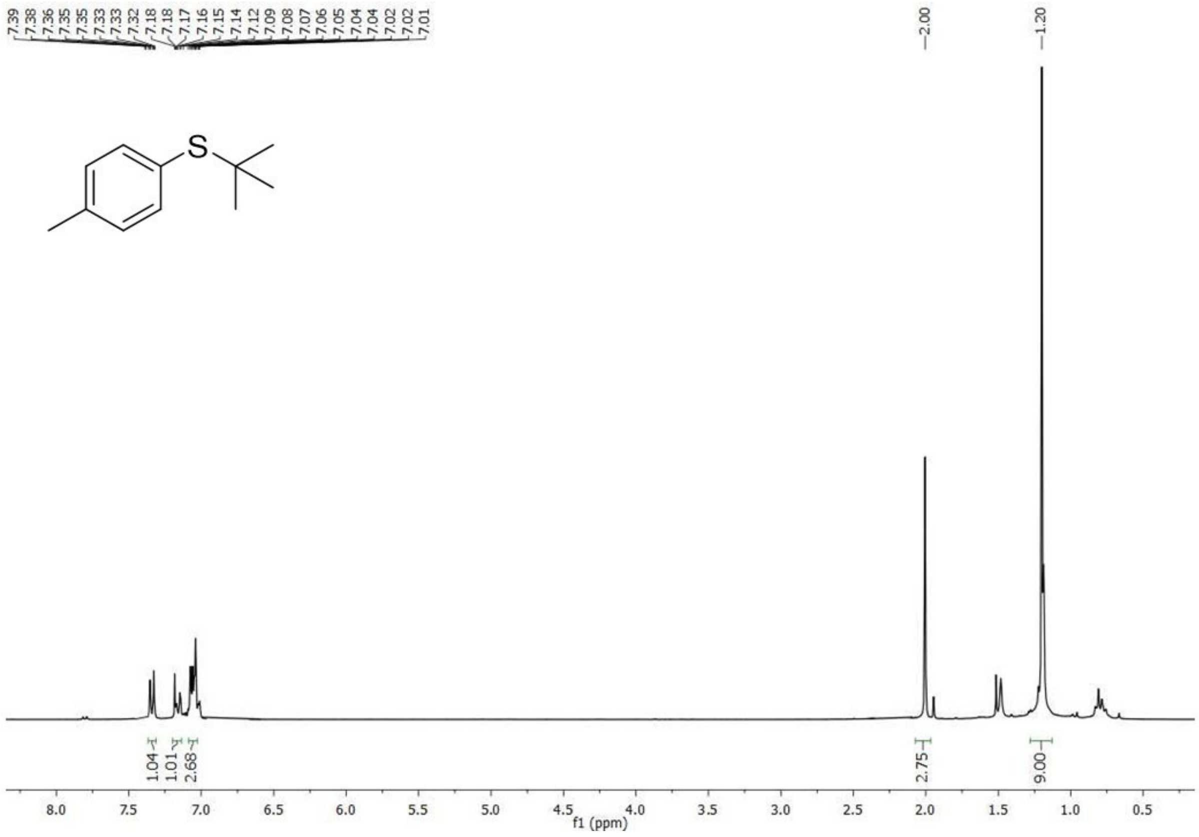

5. Single Crystal X-ray diffraction analyses

Crystals of suitable size for X-Ray diffraction analysis were selected, coated with dry perfluoropolyether (FOMBLIN) oil, mounted on a glass fiber, and fixed in a cold nitrogen stream (T = 173 K or T = 193 K) to the goniometer head. Data collection was performed using either a Bruker-Nonius X8 kappa APEX II CCD area detector or a Bruker-AXS, D8 QUEST ECO, PHOTON II area detector diffractometer, employing monochromatic Mo Kα radiation (λ = 0.71073 Å) and ω and ϕ scans with a step size of 0.50°. Data were reduced using SAINT[16] and corrected for absorption effects using the multi-scan method SADABS.[17] Structures were solved by intrinsic phasing modification of direct methods (SHELXT)[18] and refined against all F^2 data using full-matrix least-squares methods (SHELXL-2018/3),[19] minimizing $w[F_o^2-F_c^2]^2$. All non-hydrogen atoms were refined anisotropically. Hydrogen atoms were placed in geometrically calculated positions and refined using the riding model, with isotropic displacement parameters. Geometric restraints (DFIX), anisotropic displacement parameter (ADP) restrain (SIMU) and the rigid-bond restraints (DELU and RIGU) were applied to achieved more reasonable geometry and ADP values of the atoms on moderately librating groups. The ISOR command was also used to restraint the anisotropic displacement parameters of certain atoms, promoting more isotropic behavior.

A summary of the fundamental crystallographic and refinement data of compound **1**, **2b**, **2c**, **3b** and **4** is provided in **Tables S1 - S5**. Crystallographic data for the structures reported in this article have been deposited in the Cambridge Crystallographic Data Centre (CCDC) under the deposition number 2467385 (**1**), 2467386 (**2b**), 2467387 (**2c**), 2467388 (**3b**), and 2467389 (**4**) and can be obtained free of charge from the CCDC via https://www.ccdc.cam.ac.uk/structures/.

[16] Bruker. *SAINT+* **2007**, Bruker AXS Inc., Madison, Wisconsin, USA.

[17] Sheldrick, G. M. SADABS, Programs for Scaling and Absorption Correction of Area Detector Data **1997**, University of Göttingen: Göttingen, Germany.

[18] Sheldrick, G. M. SHELXT - Integrated Space-Group and Crystal-Structure Determination. *Acta Crystallogr. Sect. A* **2015**, *71*, 3.

[19] Sheldrick, G. M. Crystal Structure Refinement with SHELXL. *Acta Crystallogr. Sect. C* **2015**, *71*, 3.

Table S1. Crystal data and structure refinement for 1

Identification code	1_cm00316-def
Empirical formula	$C_{27}H_{28}NO_3P$
Formula weight	445.47
Temperature/K	173(2)
Crystal system	orthorhombic
Space group	Fdd2
a/Å	13.1751(3)
b/Å	86.924(2)
c/Å	8.3448(3)
α/°	90
β/°	90
γ/°	90
Volume/Å^3	9556.7(5)
Z	16
ρ_{calc}g/cm^3	1.238
μ/mm^{-1}	0.143
F(000)	3776.0
Crystal size/mm^3	0.34 × 0.28 × 0.25
Radiation	MoKα (λ = 0.71073)
2Θ range for data collection/°	3.748 to 61.374
Index ranges	-17 ≤ h ≤ 18, -124 ≤ k ≤ 115, -11 ≤ l ≤ 11
Reflections collected	43710
Independent reflections	7317 [R_{int} = 0.0257, R_{sigma} = 0.0185]
Data/restraints/parameters	7317/1/305
Goodness-of-fit on F^2	1.170
Final R indexes [I>=2σ (I)]	R_1 = 0.0473, wR_2 = 0.1252
Final R indexes [all data]	R_1 = 0.0521, wR_2 = 0.1309
Largest diff. peak/hole / e Å^{-3}	0.36/-0.42
Flack parameter	0.027(19)

Table S2. Crystal data and structure refinement for 2b.

Identification code	2b_tr00420a-def
Empirical formula	$C_{34}H_{33}BrCuN_2O_2P$
Formula weight	676.04
Temperature/K	193.00
Crystal system	monoclinic
Space group	$P2_1/n$
a/Å	11.8730(4)
b/Å	14.6922(5)
c/Å	18.6907(6)
α/°	90
β/°	106.4303(13)
γ/°	90
Volume/Å^3	3127.27(18)
Z	4
ρ_{calc}g/cm^3	1.436
μ/mm^{-1}	2.060
F(000)	1384.0
Crystal size/mm^3	0.28 × 0.22 × 0.16
Radiation	MoKα (λ = 0.71073)
2Θ range for data collection/°	4.526 to 56.596
Index ranges	$-15 \leq h \leq 15$, $-19 \leq k \leq 19$, $-24 \leq l \leq 24$
Reflections collected	193080
Independent reflections	7765 [R_{int} = 0.0527, R_{sigma} = 0.0150]
Data/restraints/parameters	7765/0/376
Goodness-of-fit on F^2	1.102
Final R indexes [I>=2σ (I)]	R_1 = 0.0274, wR_2 = 0.0644
Final R indexes [all data]	R_1 = 0.0419, wR_2 = 0.0743
Largest diff. peak/hole / e Å^{-3}	0.43/-0.64

Table S3. Crystal data and structure refinement for 2c.

Identification code	2c_ec00718a
Empirical formula	$C_{32}H_{31}N_4PCuBr$
Formula weight	646.03
Temperature/K	193(2)
Crystal system	triclinic
Space group	P-1
a/Å	8.5332(4)
b/Å	11.4089(6)
c/Å	16.0520(9)
α/°	84.663(3)
β/°	85.321(3)
γ/°	72.587(3)
Volume/Å^3	1482.28(14)
Z	2
ρ_{calc}g/cm^3	1.447
μ/mm^{-1}	2.166
F(000)	660.0
Crystal size/mm^3	0.2 × 0.1 × 0.05
Radiation	MoKα (λ = 0.71073)
2Θ range for data collection/°	4.688 to 50.498
Index ranges	$-10 \le h \le 9$, $-12 \le k \le 13$, $-19 \le l \le 19$
Reflections collected	12365
Independent reflections	5341 [R_{int} = 0.0333, R_{sigma} = 0.0558]
Data/restraints/parameters	5341/2/362
Goodness-of-fit on F^2	0.957
Final R indexes [I>=2σ (I)]	R_1 = 0.0418, wR_2 = 0.1025
Final R indexes [all data]	R_1 = 0.0647, wR_2 = 0.1096
Largest diff. peak/hole / e Å^{-3}	0.53/-0.47

Table S4. Crystal data and structure refinement for 3b.

Identification code	3b_ec00418a
Empirical formula	$C_{147}H_{148}N_{12}O_8F_{24}P_8Cl_8Cu_4$
Formula weight	3452.29
Temperature/K	193(2)
Crystal system	monoclinic
Space group	$P2_1/n$
a/Å	13.9213(14)
b/Å	15.2466(13)
c/Å	37.608(3)
α/°	90
β/°	98.088(5)
γ/°	90
Volume/Å^3	7903.1(12)
Z	2
ρ_{calc}g/cm^3	1.451
μ/mm^{-1}	0.834
F(000)	3532.0
Crystal size/mm^3	0.1 × 0.05 × 0.03
Radiation	MoKα (λ = 0.71073)
2Θ range for data collection/°	3.004 to 52.622
Index ranges	$-17 \leq h \leq 7$, $-16 \leq k \leq 18$, $-41 \leq l \leq 46$
Reflections collected	67980
Independent reflections	14503 [R_{int} = 0.1747, R_{sigma} = 0.2086]
Data/restraints/parameters	14503/183/979
Goodness-of-fit on F^2	1.078
Final R indexes [I>=2σ (I)]	R_1 = 0.1179, wR_2 = 0.2872
Final R indexes [all data]	R_1 = 0.2532, wR_2 = 0.3508
Largest diff. peak/hole / e Å^{-3}	1.08/-0.62

Table S5. Crystal data and structure refinement for 4.

Identification code	4_ec00818a
Empirical formula	$C_{65}H_{63}BN_8F_4P_2Cl_4Cu_2$
Formula weight	1373.86
Temperature/K	193(2)
Crystal system	monoclinic
Space group	$P2_1/c$
a/Å	17.7749(10)
b/Å	15.9796(9)
c/Å	22.5680(14)
α/°	90
β/°	96.691(2)
γ/°	90
Volume/Å^3	6366.5(6)
Z	4
ρ_{calc}g/cm^3	1.433
μ/mm^{-1}	0.945
F(000)	2824.0
Crystal size/mm^3	0.45 × 0.25 × 0.2
Radiation	MoKα (λ = 0.71073)
2Θ range for data collection/°	4.804 to 50.498
Index ranges	$-17 \le h \le 21$, $-19 \le k \le 14$, $-27 \le l \le 21$
Reflections collected	63136
Independent reflections	11515 [R_{int} = 0.0383, R_{sigma} = 0.0277]
Data/restraints/parameters	11515/152/820
Goodness-of-fit on F^2	1.039
Final R indexes [I>=2σ (I)]	R_1 = 0.0359, wR_2 = 0.0881
Final R indexes [all data]	R_1 = 0.0520, wR_2 = 0.0949
Largest diff. peak/hole / e Å^{-3}	0.57/-0.53
